# When less is more: harnessing optimal quencher concentrations to maximize quantum yields in TADF photoredox reactions

**DOI:** 10.1039/d6sc06836g

**Published:** 2026-09-28

**Authors:** Federica Fina, Matthias Schmitz, Maria-Sophie Bertrams, Caterina Bellatreccia, Chris Burdenski, Max M. Hansmann, Paola Ceroni, Christoph Kerzig

**Affiliations:** a Department of Chemistry, Johannes Gutenberg University Mainz 55128 Mainz Germany ckerzig@uni-mainz.de; b Alma Mater Studiorum-Dipartimento di Chimica “G. Ciamician”, Università di Bologna Via Gobetti 85 Bologna 40129 Italy paola.ceroni@unibo.it; c Fakultät für Chemie und Chemische Biologie, Technische Universität Dortmund Otto-Hahn-Str. 6 44227 Dortmund Germany

## Abstract

Here, we report a counterintuitive “less is more” effect, in which lower quencher concentrations improve overall quantum yields in photoinduced electron transfer – key reaction steps in organic photoredox catalysis. This behavior arises because lower quencher concentrations favor quenching of the triplet rather than the singlet excited state. In the latter undesired case, lower efficiencies for free radical or radical ion formation following electron transfer are generally expected, due to reduced cage escape (*i.e.*, separation of geminate radical pairs before recombination). This less productive pathway kinetically competes with the more productive triplet channel. Using nanosecond transient absorption spectroscopy, we directly quantified state-specific cage escape efficiencies and incorporated them into a model to predict radical production quantum yields across different quencher concentration regimes. Additionally, we assessed how the TADF-specific photophysical properties contribute to the observed behavior and examined how these processes translate into catalytic efficiency under continuous irradiation. In contrast to expectations derived from conventional emission quenching experiments, our results clearly demonstrate – across four photocatalyst-quencher systems and three solvents spanning a wide polarity range – that dilution of a reaction system or avoiding a quencher excess can greatly improve the achievable reaction quantum yields in photoredox catalysis. This behavior is not only demonstrated by quantitative transient absorption spectroscopy but also by an initial lab-scale photocatalysis experiment. These findings have important implications in the context of maximizing quantum yields of photoreactions, which is essential for enabling competitive applications on a larger scale.

## Abstract 2

Lower quencher concentrations favor triplet over singlet quenching, enhancing radical yield *via* higher cage escape efficiency. This “less is more” strategy suits TADF and conventional triplet-populating photocatalysts, boosting photoredox efficiency.

## Graphical abstract

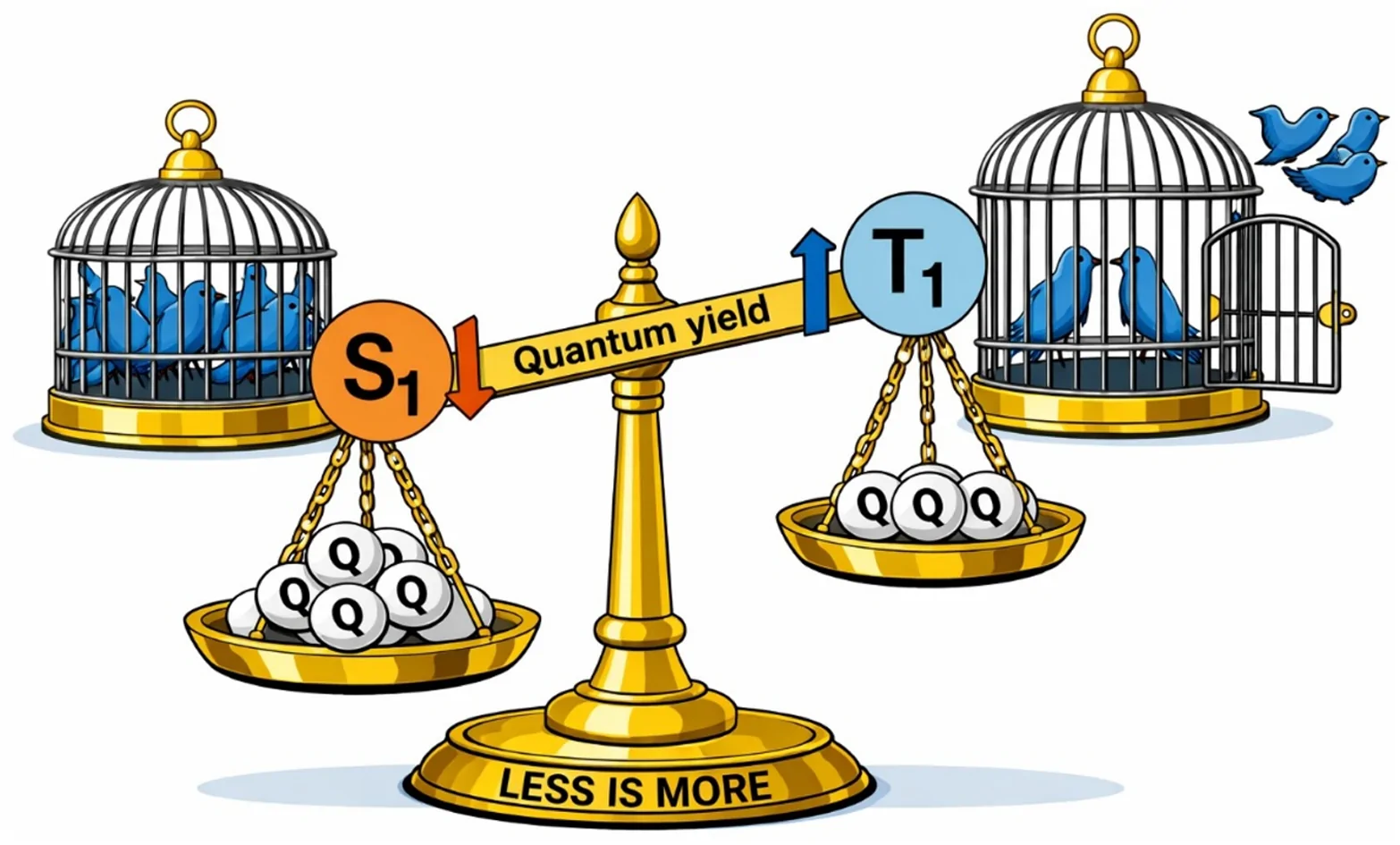

## Introduction

Photoredox catalysis has become a powerful tool in contemporary chemistry, unlocking complex molecular transformations and enabling new synthetic pathways through excited-state reactivity.^1–9^ The photoredox catalytic cycle typically begins with the excitation of a photocatalyst by light, subsequently undergoing a bimolecular interaction with a redox-active sacrificial quencher or directly with a substrate. The most common method to evaluate the feasibility of this initial key step is Stern–Volmer analysis monitoring the bimolecular quenching of the photocatalyst's excited state emission by the quencher.^10–14^ Since this step enables subsequent reactivity, it is intuitive to seek high quenching efficiency, *i.e.* using high quencher concentrations, under the assumption that maximizing this term will proportionally enhance the overall catalytic yield.^15–17^ However, it has been increasingly observed that a high quenching efficiency does not necessarily mirror productive reaction pathways: luminescence quenching establishes the rate and primary efficiency of electron-transfer processes, but it is unable to capture the subsequent processes that lead to the formation of the truly active species, namely free radicals or radical ions in solution.^18–22^ After photoinduced electron transfer, the newly formed radicals are not yet free, but exist as geminate radical pairs trapped within a so-called solvent cage, from which they must escape to unlock their reactivity.^23^ The primary competitive deactivation pathway is geminate charge recombination, a back-electron transfer process that restores both the photocatalyst and the quencher to their respective ground states (process labelled with CR in Fig. 1A). Consequently, the photoinduced electron transfer event, although observed through luminescence quenching experiments, may prove non-productive for the intended chemical transformation. This results in an overall loss of quantum efficiency, as the energy utilized to populate the photocatalyst's excited state is dissipated rather than contributing to the generation of the free radicals required for the catalytic cycle to proceed. Alternatively, the desired productive cage escape pathway (process labeled CE in Fig. 1A) produces the reactive one-electron activated intermediates.

**Fig. 1:**
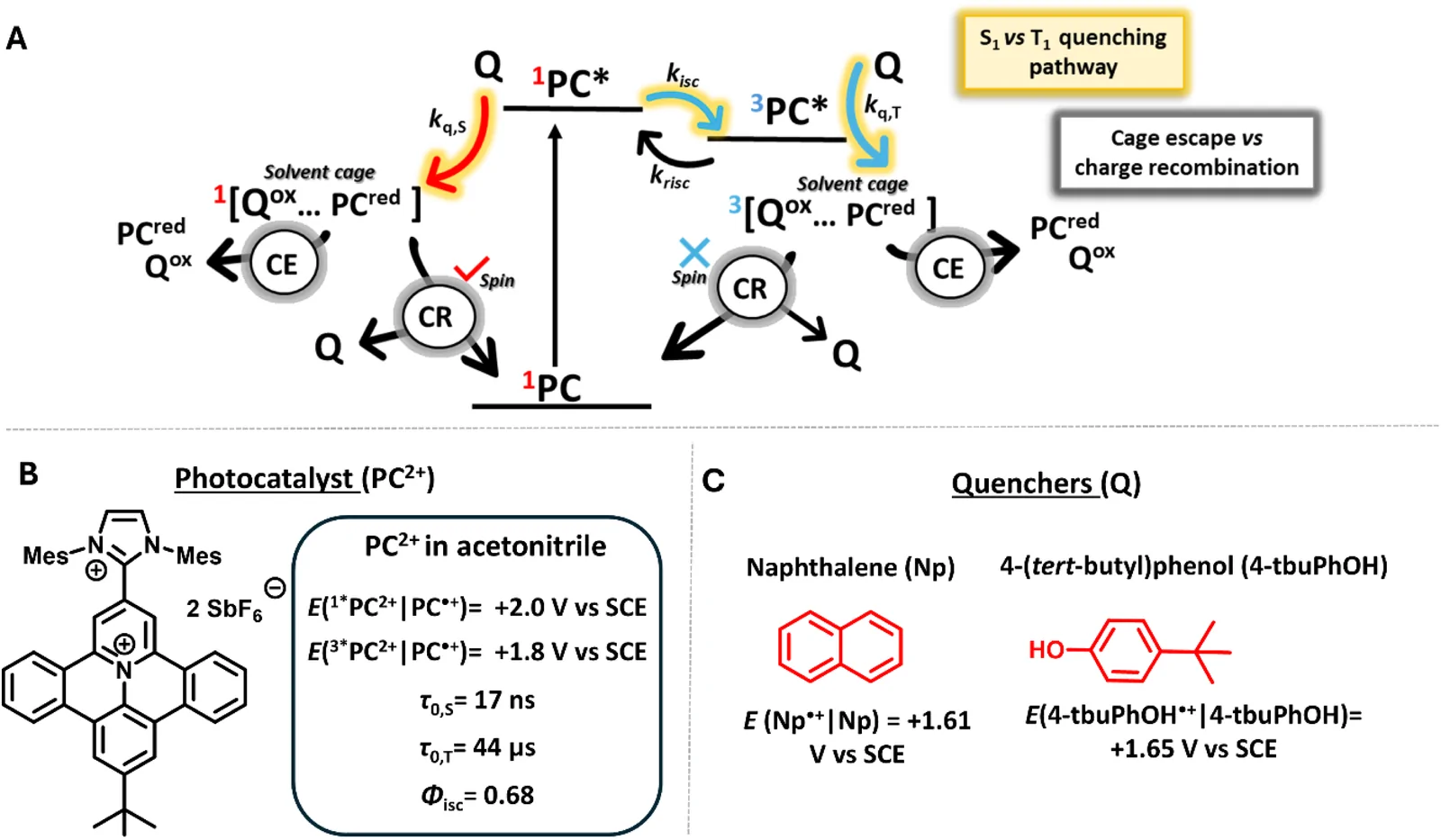
(A) Spin-dependent pathways following excitation of a general TADF photocatalyst (PC), showing competition between S_1_ and T_1_ quenching and, after electron transfer, between cage escape (CE) and geminate charge recombination (CR). (B) Selected TADF photocatalyst (PC^2+^) and relevant photophysical characteristics in acetonitrile solution. (C) Quenchers used as electron donors and their oxidation potentials.

Over the past decades, considerable effort has been directed toward elucidating the factors controlling cage escape, including solvent properties, ionic strength, temperature, and the electrostatic characteristics of the reacting species, and there is currently a renewed surge of interest in this topic.^17,18,23–36^ Within this framework, the objective of the present work is to focus specifically on the spin state of the excited photocatalyst as a critical parameter for subsequent cage escape. In our work, we target to quantify this productivity discrepancy by employing photocatalysts exhibiting thermally activated delayed fluorescence (TADF), widely used in photoredox catalysis, as their unique singlet–triplet interplay offers an ideal platform to explore and control spin-dependent processes, by pairing them with quencher concentration effects.^37–43^ Among their peculiar photophysical properties, they indeed exhibit, due to an appropriately designed structure, first excited singlet (S_1_) and triplet (T_1_) states in close energetic proximity (Δ*E*_S–T_ < 0.3 eV).^44,45^ Accordingly, a biexponential fluorescence decay is observed: prompt fluorescence occurring on the nanosecond timescale from direct S_1_ emission, and delayed fluorescence generally occurring on the microsecond timescale from S_1_ repopulation *via* T_1_ through reverse intersystem crossing (risc). The interplay between these excited states, their lifetimes, and their close energetic proximity, leading to similar redox potentials, allows both states to participate effectively in photoredox catalysis providing similar excited state redox potentials.^46,47^ Owing to the longer lifetime of T_1_, low quencher concentrations are typically sufficient to achieve efficient quenching. In contrast, quenching of the much shorter-lived S_1_ necessitates significantly higher quencher concentrations to achieve high quenching efficiencies. Importantly, isc to the T_1_ state competes with quenching of the S_1_ state, reducing the amount of T_1_ state formed with increasing S_1_ quenching efficiency. It seems natural to assume that the factor critically differentiating the outcome of the quenching event from either state is the spin state of the photogenerated radical pair. As these pairs are initially generated with spin retention, photoinduced electron transfer from S_1_ yields a geminate pair in a singlet state, whereas T_1_ quenching results in a triplet-state geminate radical pair.^48^ Charge recombination is expected to be more relevant for radical pairs originating from S_1_ quenching, since the ground-state reactants are in a singlet state and recombination therefore proceeds *via* a spin-allowed pathway. In contrast, triplet radical pairs confined within the solvent cage are expected to display higher cage escape yields, as the competing recombination process is spin-forbidden (see Fig. 1A).^23,26,29,49,50^ While the fact that cage escape yields for triplet chromophores are in general higher than those of chromophores with photoactive singlet state is well established in the literature, experimental determination of relative cage escape yields for chromophores with dual reactivity from both S_1_ and T_1_ are still scarce.^50,51^ This is surprising at first glance as several widely used photocatalysts with such a dual reactivity are commonly employed in photocatalysis.^1,37,38^

The expected different cage escape behavior is supported by a recent work, in which Ishitani *et al.* identified a negative correlation between the concentration of the sacrificial electron donor and the overall catalytic efficiency, using the benchmark TADF photocatalysts 4DPAIPN and 3DPAFIPN for CO_2_ reduction.^36^ This is attributed to the fact that at lower quencher concentrations the triplet quenching pathway is dominant, showing a higher cage escape yield. While being foundational, these findings rely on some fundamental approximations, as the cage escape efficiency was not directly measured, but deduced from kinetic modelling and fitting procedures based on donor concentration-dependent reaction yields. Most notably, this top-down approach cannot experimentally distinguish between primary geminate in-cage and bulk (out-of-cage) recombination, that may obscure the true kinetic efficiency of the initial cage escape. Additionally, the mathematical treatment does not account for repopulation of the singlet state *via* reverse intersystem crossing in a TADF emitter, and the potential impact of this approximation was not further evaluated. To address these limitations, our work utilizes transient absorption spectroscopy to provide a direct quantification of spin-related cage escape.^31,35,52,53^ We first examine a recently synthesized TADF photocatalyst^54^ paired with two different reductive quenchers (Fig. 1B and C). By monitoring free radicals' formation in real-time, we bypass the inherent blind spots of an end-of-reaction analysis. Successively, we put these results in the context of exact TADF kinetic equations that explicitly account for the repopulation of the singlet state *via* reverse intersystem crossing, to verify how S_1_–T_1_ coupling influences the radical yield and confirm the robustness of the concentration dependent paradigm even under a rigorous TADF treatment. This comprehensive framework is finally extended to build a bottom-up prediction for reactions under continuous irradiation, effectively bridging the gap between fundamental time-resolved data and operative catalytic conditions. As we will show in the last part of our manuscript, the “less is more” concept can be successfully applied to the reductive quenching of the well-known and widely used TADF emitter 4CzIPN and to the non-TADF emitter photocatalyst SO_2_TDS^2−^ in an aqueous environment, for which an enhanced reaction performance can be observed for lower quencher concentrations in lab-scale irradiation experiments.

## Results and discussion

The system identified for the quantification of radicals produced upon S_1_ or T_1_ quenching is depicted in Fig. 1B and C. The dicationic TADF-photocatalyst PC^2+^, consisting of a π-extended heterocycle/carbene hybrid, has recently demonstrated suitable for a variety of energy and electron transfer photocatalytic transformations with a wide scope of quenchers or substrates that can be activated *via* one-electron oxidation. This is due to its highly oxidizing singlet and triplet excited states in energetic proximity and a long-lived T_1_ state,^54^ while offering the additional advantage of superior photostability compared to benchmark cyanoarenes that can be susceptible to degradative substitution at the cyano groups.^39,55,56^

The marked lifetime difference between the S_1_ (<20 ns) and T_1_ state (>40 µs) enabled us a reliable assessment of the relative contributions of the individual quenching pathways and, consequently, the quantification of the radicals generated over a wide quencher concentration range spanning 0.1–200 mM. The first two quenchers identified for this study, naphthalene (Np) and 4-*tert*-butylphenol (4-*t*buPhOH), possess oxidation potentials (+1.61 V and +1.65 V *vs.* SCE, respectively)^57,58^ that thermodynamically enable quenching of S_1_ and T_1_ and, according to literature, their ground states or oxidized forms should not interfere with our spectroscopic measurements on the excited state quenching of PC^2+^.^59–63^ The reduced photocatalyst PC˙^+^ shows clear (transient) absorption bands across our most sensitive detection range (350–700 nm, Fig. S4 in the SI) and remains stable under our experimental conditions, allowing us to directly follow its formation by relying on its strong transient absorption signal at 450 nm.

### Spectroscopic evidence of the “less is more” effect

Before quantifying and describing the individual contributions to the overall radical yield in detail, we intent to present direct spectroscopic evidence that lower quencher concentrations indeed lead to more efficient production of free radical species.

For the first quencher employed, 4-*tert*-butylphenol, at low concentration (0.3 mM), the production of radicals arises from triplet-state quenching (*Φ*_q,T_ = 0.65), as the singlet-state contribution (*Φ*_q,S_ = 0.03) remains minimal (quenching experiments in Section 2.3 of the SI). This results in a pronounced transient absorption signal at 450 nm, consistent with efficient formation of the reduced species PC˙^+^ as shown in Fig. 2 (blue trace). Concomitant absorption by the oxidized phenolic quencher at that wavelength was ruled out (for details see SI, Section S2.6).

**Fig. 2:**
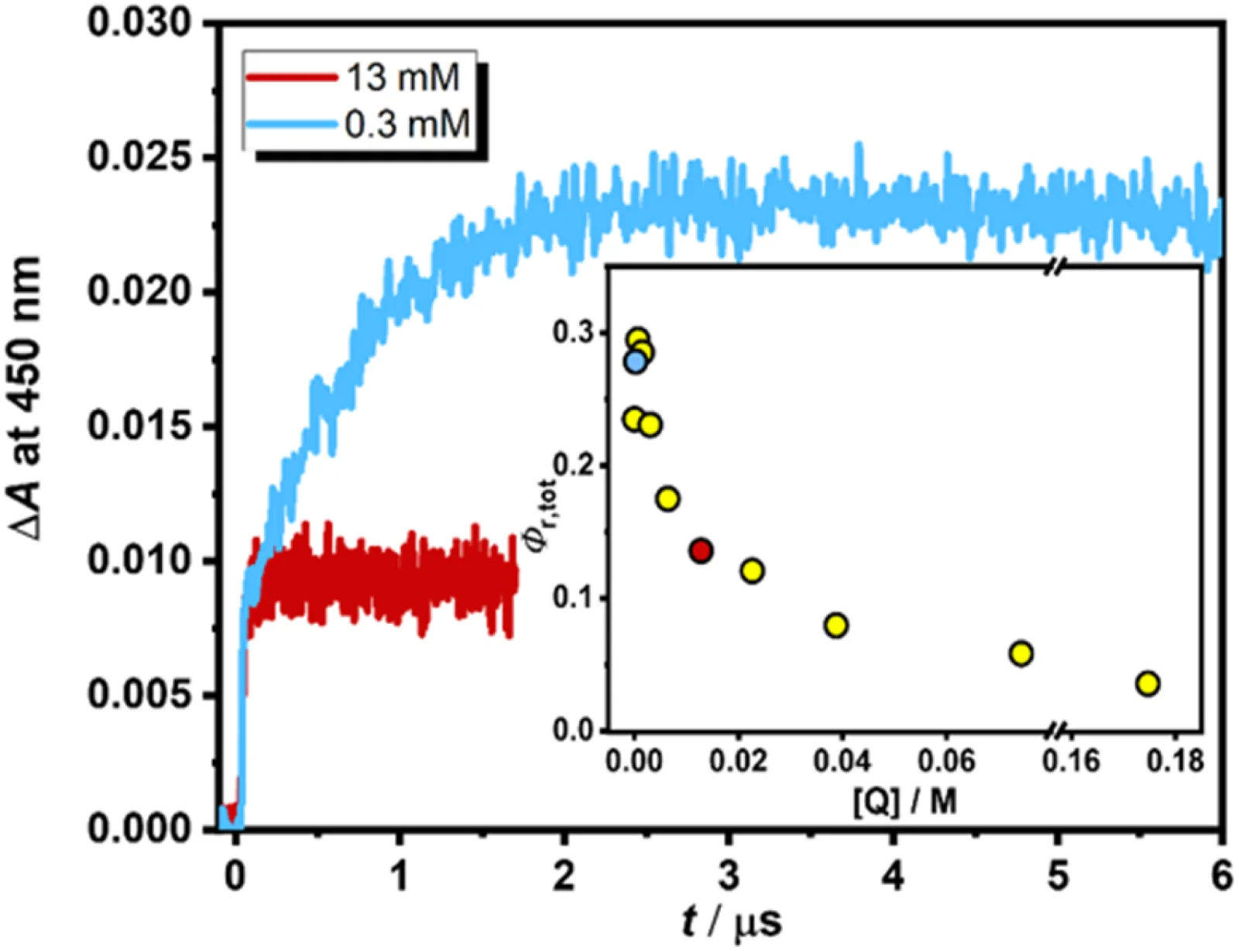
Time-resolved absorption traces recorded at *λ* = 450 nm, corresponding to PC˙^+^ generated *via* photoinduced electron transfer, upon 355 nm laser excitation (5.5 mJ per pulse) of two iso-absorbing acetonitrile solutions containing 5 µM PC^2+^ and different concentrations of 4-*t*buPhOH. Inset: measured quantum yields for free radical production as function of quencher concentration. Further explanations are given in the text.

Conversely, at a higher quencher concentration (13 mM, red trace), the contribution of the triplet quenching is reduced (*Φ*_q,T_ = 0.30), while that of the singlet is concomitantly increased (*Φ*_q,S_ = 0.54). A direct comparison of the signal intensity of these time-resolved traces – while keeping all other parameters unmodified – provides first clear spectroscopic evidence of the “less is more” phenomenon: despite the higher total quenching efficiency at 13 mM, the overall quantum yield for the production of free radicals is markedly lower. This parameter, *Φ*_r,tot_, represents the concentration of free radical generated per number of excited states produced upon photon absorption, according to eqn (1). Given the 1 : 1 stoichiometry of the process, the numerator in the fraction may either refer to the reduced photocatalyst (PC˙^+^) or the oxidized quencher (Q˙^+^).1
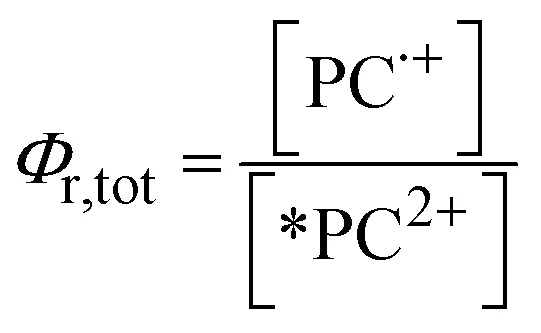


The inset of Fig. 2 provides a comprehensive exploration of the concentration dependent free radical production quantum yield across a wider concentration range for the phenol quencher. For each data point, *Φ*_r,tot_ was determined according to eqn (1), with the concentration of PC˙^+^ calculated by the Beer–Lambert law using the Δ*ε* (procedure for the determination in the SI, Section S2.1) and the averaged transient absorption signal (Δ*A*) at 450 nm within a specific time window. The time window, typically a few microseconds in duration, was selected to capture the signal of PC˙^+^ immediately following cage escape but prior to any bulk-phase back electron transfer (an example is given in Fig. 3B), which occurs on a much slower timescale of hundreds of microseconds (Fig. S16 of the SI). The pertinent catalyst-derived spectra, whose knowledge is essential for quantitative measurements, are shown in Fig. 3A (see SI, Sections S2.1 and S2.2 for further details).

**Fig. 3:**
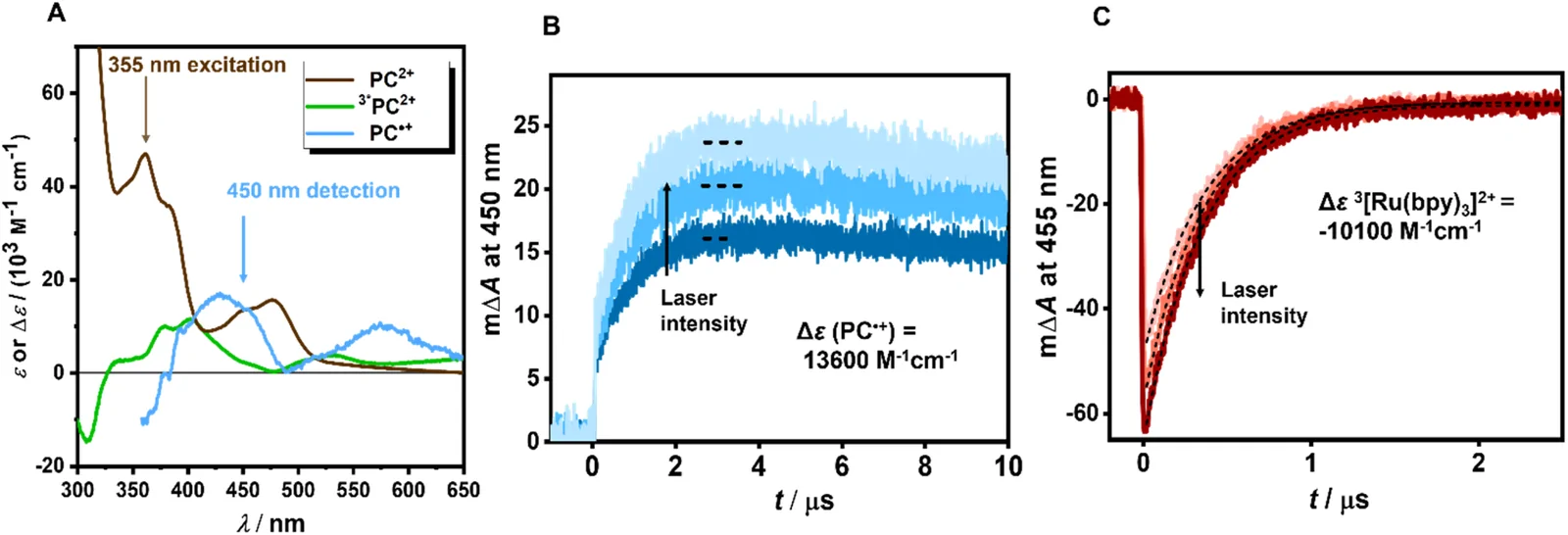
(A) Calibrated steady-state absorption spectrum of PC^2+^ in solution (brown trace), together with transient absorption spectra of PC^2+^-derived species in MeCN solution upon photoexcitation, showing the triplet ^3*^PC^2+^ (green trace) and the radical species PC˙^+^ formed after electron transfer when in presence of a reductive quencher (blue trace). Excitation and detection wavelength selected during measurements are indicated. (B) Time-resolved absorption traces at 450 nm under different laser excitation intensities (5–8 mJ per pulse) of a ∼5 µM PC^2+^ solution containing a low concentration (0.22 mM) of 4-*t*buPhOH, to selectively quench the triplet excited state. The black dashed lines indicate the microsecond time window used for Δ*A* averaging to quantify the radical concentration after the cage escape event. (C) Time-resolved ground state bleach traces at 455 nm of an iso-absorbing aqueous solution of [Ru(bpy)_3_]Cl_2_ recorded under identical experimental conditions as actinometric reference.

The number of excited states (*PC^2+^) produced upon laser excitation was quantified *via* relative actinometry (Fig. 3C) using [Ru(bpy)_3_]^2+^ as a reference (see SI, Section S2.4).^64,65^ The resulting quantum yield for the production of free radical across the full concentration range clearly shows that progressively increasing the quencher concentration leads to a lower quantum yield of free radical production.

Analogous results were obtained for the second case study, using naphthalene as the quencher. Shifting concentration from 54 mM, where the singlet excited state dominates quenching (*Φ*_q,T_ = 0.18 and *Φ*_q,S_ = 0.75), down to 0.04 mM, where the triplet state prevails (*Φ*_q,T_ = 0.67 and *Φ*_q,S_ = 0.02), resulted in a two-fold increase in the overall transient absorption signal of PC˙^+^ (Fig. S14a of the SI). Similarly, the trend observed across a concentration range of 0.1–200 mM consistently demonstrated a decrease in efficiency at higher concentrations, further reinforcing the robustness of the spin-selective quenching paradigm (Fig. S14b in the SI).

### Determination of singlet and triplet cage escape efficiency

Given the experimental evidence that the triplet channel is intrinsically more productive for radical generation, we sought to elucidate the individual processes in more detail by quantification of the discrepancy in cage escape efficiencies (*η*_ce_) from each excited state. While the transient absorption traces displayed in Fig. 2 provide a direct visual comparison of radical formation, a simple proportionality between the total signal amplitude and individual state efficiencies cannot be assumed. Considering that the triplet state is populated *via* intersystem crossing from the singlet state, triplet population, and consequently its quenching quantum yield, is inherently dependent on the preceding singlet-state kinetics and on the efficiency of the isc step. To properly disentangle these contributions and account for the individual cage escape efficiencies for singlet (*η*_ce,S_) and triplet excited states (*η*_ce,T_), we applied a mathematical treatment based on the following equations. We define the total quantum yield of free radical formation (*Φ*_r,tot_) consisting of the sum of the singlet (*Φ*_r,S_) and the triplet (*Φ*_r,T_) state contributions as follows:

2 *Φ*_r,tot_ = *Φ*_r,S_ + *Φ*_r,T_

3
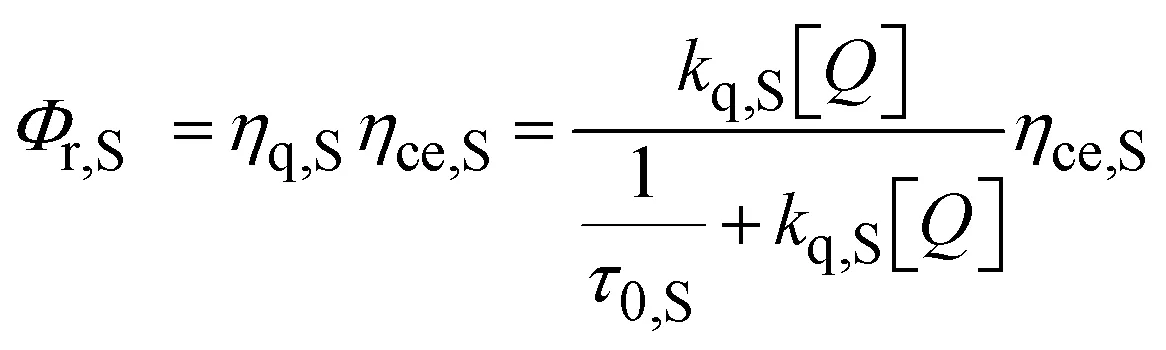
4
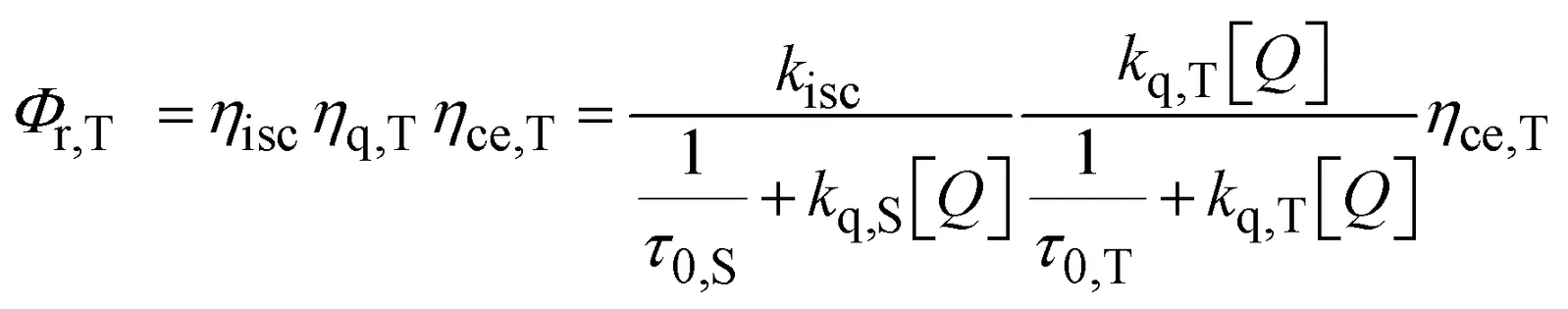


The determination of state-specific cage escape efficiency values was conducted through two distinct experimental regimes, each designed to rely on the preferential quenching of one state (Table 1).

**Table 1:** Quencher concentrations, related efficiencies and quenching quantum yields used to determine cage escape efficiencies in experiment series 1a and 1b. Quenching efficiencies and quantum yields were calculated using experimentally determined quenching constants as described in Section S2.3 of the SI

Exp.	[4-*t*buPhOH]/mM	*η*_q,S_	*η*_isc_	*Φ*_q,T_ (= *η*_isc_ × *η*_q,T_)
1a	0.22	0.02	0.66	0.65
1b	132	0.94	0.05	0.05

Experiment series 1a was performed at a sufficiently low phenol concentration (0.22 mM) to favor the triplet-state quenching pathway. Under these conditions, the singlet-state quenching efficiency *η*_q,S_ is as low as 0.02 and thus, essentially all detected radicals can be considered as those that escaped the geminate radical pair from a triplet precursor. In this low-concentration regime, the singlet state is converted into the triplet state *via* intersystem crossing with an efficiency of 0.66. This value is very close to the one measured in the absence of a quencher (0.68, see Fig. 1).^54^ Consequently, the triplet population approaches a value near its theoretical maximum, and its long lifetime allows for efficient bimolecular quenching. The transient absorption spectrum recorded with a 5 µs delay (Fig. S18 in the SI) exhibits the characteristic signatures of both the reduced photocatalyst (PC˙^+^) and the phenoxyl radical, with a characteristic peak near 405 nm.^66^ Contributions to the detected transient absorption signal from oxidized phenolic species at 450 nm were ruled out based on literature reports.^59,60^ Therefore the signal intensity at this wavelength was used to quantify the photocatalyst reduced form PC˙^+^ (further discussion in the SI, Section S2.6). Time-resolved absorption traces were accordingly recorded following 355 nm laser excitation across three pulse energies (ranging from 5 to 8 mJ per pulse), as depicted in Fig. 3B. The observed linear correlation between laser power and the radical-associated signal (Fig. S15 in the SI) confirms the absence of product photodegradation and excludes any two-photon processes in the system.^67^ This allows us to average the outcome from the three independent measurements to get more reliable results. The concentration of excited states was quantified *via* relative actinometry, as shown in Fig. 3C, following the methodology used in Sections 2.1 and 2.2 of the SI. This approach enabled the determination of the quantum yield of free radical formation from the triplet state (*Φ*_r,T_). By incorporating the known state-specific efficiencies, *η*_ce,T_ = 0.42 ± 0.02 was calculated by eqn (4). From a physical perspective, this indicates that only 42% of the geminate radical pairs resulting from a triplet quenching event successfully escape the solvent cage to participate in subsequent catalytic transformations.

An analogous experimental methodology was employed in experiment series 1b to determine the cage escape efficiency of the radical pair generated *via* singlet quenching (*η*_ce,S_). Unlike experiment series 1a, where the total radical production was attributed entirely to the triplet manifold, the experimental conditions used to probe the singlet state do not allow for exclusive S_1_ quenching, due to a residual, non-negligible triplet population (*η*_isc_ = 0.05), and the quencher concentration could not be increased further owing to solubility issues. This aspect is particularly relevant as the triplet manifold is expected to be intrinsically more productive than the singlet state. For this reason, we subtracted the triplet contribution to the total free radical production quantum yield according to eqn (2). From the resulting singlet-derived quantum yield of free radical production (*Φ*_r,S_), the cage escape efficiency was obtained, according to eqn (3), yielding a value of *η*_ce,S_ = 0.028 ± 0.005 for the phenol system. The inherent efficiency of the triplet pathway is thus higher by more than one order of magnitude.

Comparable results were obtained with the second case study using naphthalene as quencher (experiment series 2a and 2b in Section S2.7 of the SI). At a low naphthalene concentration of 0.12 mM, the quenching process is dominated by the triplet manifold (*Φ*_q,T_ = 0.62, *Φ*_q,S_ = 0.01) allowing for the determination of *η*_ce,T_ = 0.46 ± 0.03. Conversely, working at 120 mM, when the singlet pathway becomes the primary deactivation channel (*Φ*_q,T_ = 0.07, *Φ*_q,S_ = 0.89), a value of *η*_ce,S_ = 0.07 ± 0.01 was obtained.

Importantly, these values provide the first quantitative determination of the expected singlet–triplet cage escape difference in a TADF photocatalyst, achieved through the direct and quantitative spectroscopic observation of the radical intermediates, highlighting the superior productivity of the triplet manifold.

### Predicting concentration-dependent radical yield

Following the quantification of these state-specific cage escape efficiencies, we evaluated the implications of the singlet–triplet difference on the overall predicted quantum yield across a broad range of donor concentrations. Utilizing the established kinetic parameters of the photocatalyst-phenol system (*k*_q,S_, *k*_q,T_, *k*_isc_), the unquenched singlet and triplet state lifetimes (*τ*_0,S_, *τ*_0,T_) and the experimentally determined cage escape efficiencies, the predictive plots for *Φ*_q_ depicted in Fig. 4 were generated. The red and blue traces isolate the contributions from the singlet and triplet states, respectively, while the black trace represents the total cumulative quenching yield (Fig. 4, left). This “quenching-only” model does not account for the possibility that free radical generation upon quenching may occur with non-unit efficiency. This model predicts a monotonic increase in radical yield with increasing donor concentrations, as the system approaches near-complete quenching. Accordingly, it would be reasonable to maximize the quencher concentration to fully harness the incident photon energy, which is opposite to our previous observations (Fig. 2). The right plot of Fig. 4 illustrates the theoretical quantum yield for the generation of free radicals, taking into account the cage escape efficiencies determined in the preceding section. At concentrations below 0.1 mM, the overall radical yield is kinetically limited by the inefficient quenching of the triplet state due to too low quencher concentrations. In this regime, the triplet manifold is populated with its maximum intrinsic efficiency, a parameter governed by the photocatalyst's unimolecular rate constants under the given experimental conditions. As demonstrated in the quenching-only model, the triplet quenching quantum yield increases monotonically within this range as the system approaches a more favorable bimolecular interaction frequency. The global maximum of the productivity curve (Fig. 4, right) is achieved at the concentration representing the optimal kinetic tradeoff: where triplet quenching is near quantitative, but singlet quenching has not yet become significant enough to hamper triplet formation.

**Fig. 4:**
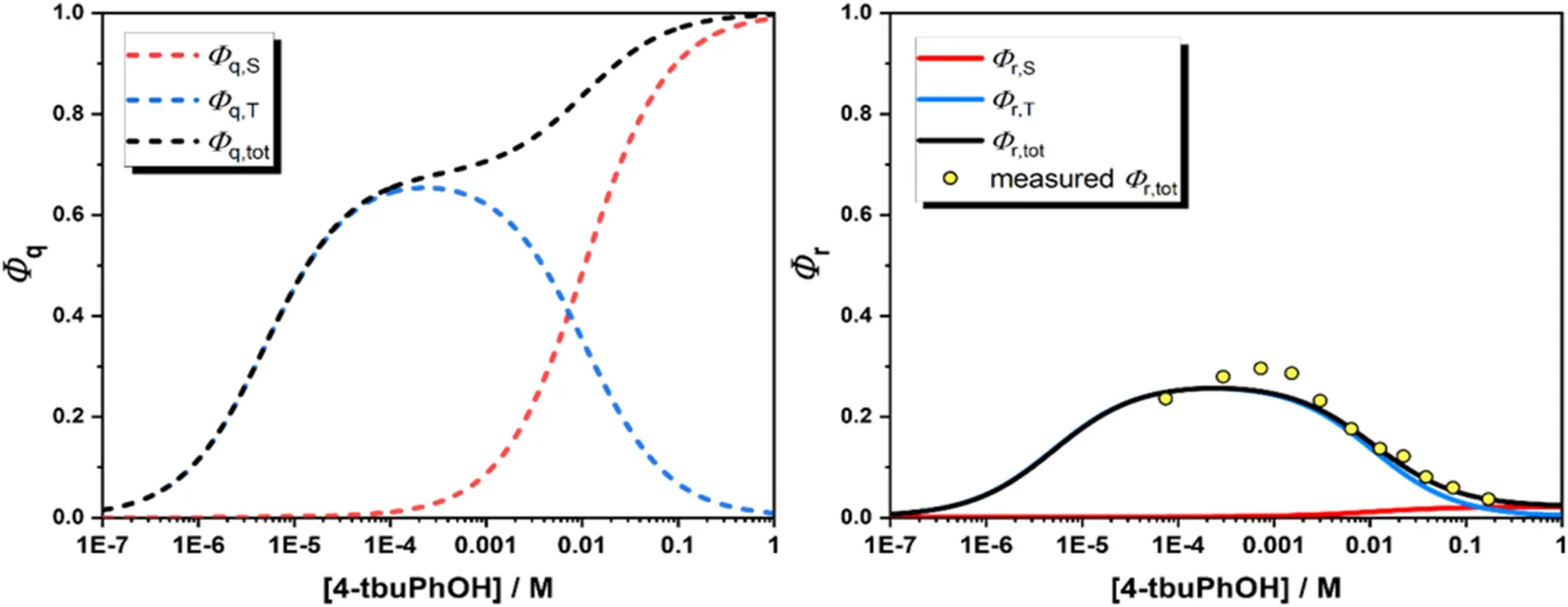
(left) Theoretical prediction of radical formation yields based solely on quenching efficiency. The red and blue lines represent the individual contributions from singlet and triplet quenching, respectively, while the black line shows the sum of the two contributions. (right) Free radical formation quantum yield, accounting for cage escape efficiencies. The red and blue lines correspond to the singlet and triplet channels, respectively, and the black line represents the total free radical formation efficiency. Yellow dots indicate experimentally measured quantum yields of free radical formation over a concentration range of 0.1 mM to 200 mM.

It should be noted that a maximum is expected to be observable only when the intersystem crossing efficiency is sufficiently high. Considering the one order of magnitude difference between singlet and triplet cage escape efficiency observed here, we estimate a minimum isc quantum yield on the order of 10% to be required for the effect to be operative when using photocatalysts with a dual singlet–triplet reactivity. The subsequent downward trend in radical yield is triggered only when singlet quenching becomes competitive with intersystem crossing (quencher concentrations >1 mM). At this stage, the deactivation of the singlet channel depletes the population of the more productive triplet manifold, which benefits from spin-forbidden charge recombination, and redirects the reaction toward a singlet-derived radical pair characterized by an inherently poor cage escape efficiency. Finally, the high-concentration region exhibits an asymptotic plateau. In this regime, the triplet manifold is virtually bypassed as the singlet state is fully intercepted by the donor prior to isc. Consequently, the electron transfer events are dominated entirely by the singlet state, and the total radical yield reflects its strikingly low cage escape efficiency. The robustness of this predictive model is corroborated by the experimental data (reproducing the yellow points from the inset of Fig. 2), which represent measured total radical formation quantum yields. This proves that the observed “less is more” effect is a predictable consequence of the competition between efficient spin-allowed recombination (for the singlet) and inefficient spin-forbidden recombination (for the triplet) during the radical generation step, when considered within the overall quenching kinetics. To further illustrate the sensitivity of *Φ*_r,tot_ to the individual photophysical parameters in a TADF system, particularly at quencher concentrations relevant to catalysis (with a focus on 50 and 100 mM), representative examples with their corresponding kinetic plots are shown in Section S4 of the SI. While the interplay of the kinetic constants and parameters is quite complex, it can be concluded that a sufficiently high isc quantum efficiency, achieved by making the singlet lifetime sufficiently short (*e.g.*, <10 ns) through a fast *k*_isc_ rate, to avoid efficient and unproductive quenching of the S_1_ state, is beneficial for maximizing the yields of free radicals (see also discussion below).

### Transferring spectroscopic predictions to continuous irradiation

A critical challenge in photoredox catalysis is translating fundamental kinetic data, mostly obtained *via* pulsed excitation (in this case quenching and cage escape measurements), into the operative conditions of continuous irradiation. While the mechanistic experiments carried out with pulsed laser excitation assume that the quencher concentration is constant during the experimental timeframe, real synthetic applications involve the progressive consumption of quenchers, leading to a dynamic reaction environment. To validate the guidelines of optimal concentration and predicted quantum yield under operative conditions, we employed a Python-based modeling framework developed in a prior study by one of our groups.^68^ We modelled the photoinduced electron transfer process of our case-study systems as the initial step of a photoreaction employing a TADF photocatalyst, where the quencher acts as a sacrificial electron donor.^40,69,70^ The quenching event results in the irreversible oxidation of one quencher molecule, phenol as sacrificial electron donor in our case, and the generation of one molecule of reduced photocatalyst. The latter can then further react with a substrate through a subsequent thermal electron transfer step, yielding the product and regenerating the original photocatalyst (PC^2+^), thus closing the catalytic cycle (mechanistic model a, shown in Fig. S26 of the SI). Our approach isolates the initial electron transfer event, the primary focus of this study, as a fundamental determinant of the catalytic outcome. Results obtained by applying a defined set of reaction conditions to the simulation code ([substrate] = 10 mM; *ε* = 1 × 10^4^ M^−1^ cm^−1^, the photocatalyst's molar absorption coefficient at the frequently used LED emission wavelength 440 nm; incident photon flux = 1 × 10^−3^ einstein s^−1^; [PC^2+^] = 1 mM), together with the experimentally determined kinetic constants and efficiencies of the 4-*t*buPhOH-PC^2+^ system, demonstrate that the optimal quencher concentrations predicted by pulsed laser flash photolysis accurately forecast reaction quantum yields under continuous irradiation (Fig. S27 of the SI). Although the highest quantum yield is predicted at quencher concentrations on the order of 1 mM, at least stoichiometric concentrations (*i.e.*, 10 mM) have to be considered in our simulation for being able to drive the reaction to full conversion. Interestingly, the analysis of the reaction progression reveals an intriguing interplay between quantum yield and substrate conversion. In a real reaction environment, the quencher is progressively consumed as a sacrificial donor, meaning its concentration decreases as the reaction progresses. Consequently, the system does not remain at a single point on the efficiency curve; instead, it “slides” toward lower quencher concentrations on the *x*-axis of the kinetic plots displayed in Fig. 4 throughout the course of the irradiation. This dynamic behavior implies that the reaction quantum yield is not constant over time, leading us to investigate how the quantum yield evolves based on the initial starting point on the curve.

To explore this, we modelled the photoreaction under continuous irradiation using the same conditions as in our previous calculations, specifically, a 10 mM substrate concentration, while comparing three initial quencher concentrations: the optimal 10 mM (stoichiometric to the substrate), and concentrations two and ten times higher, resulting in two main trends, as depicted Fig. 5:

**Fig. 5:**
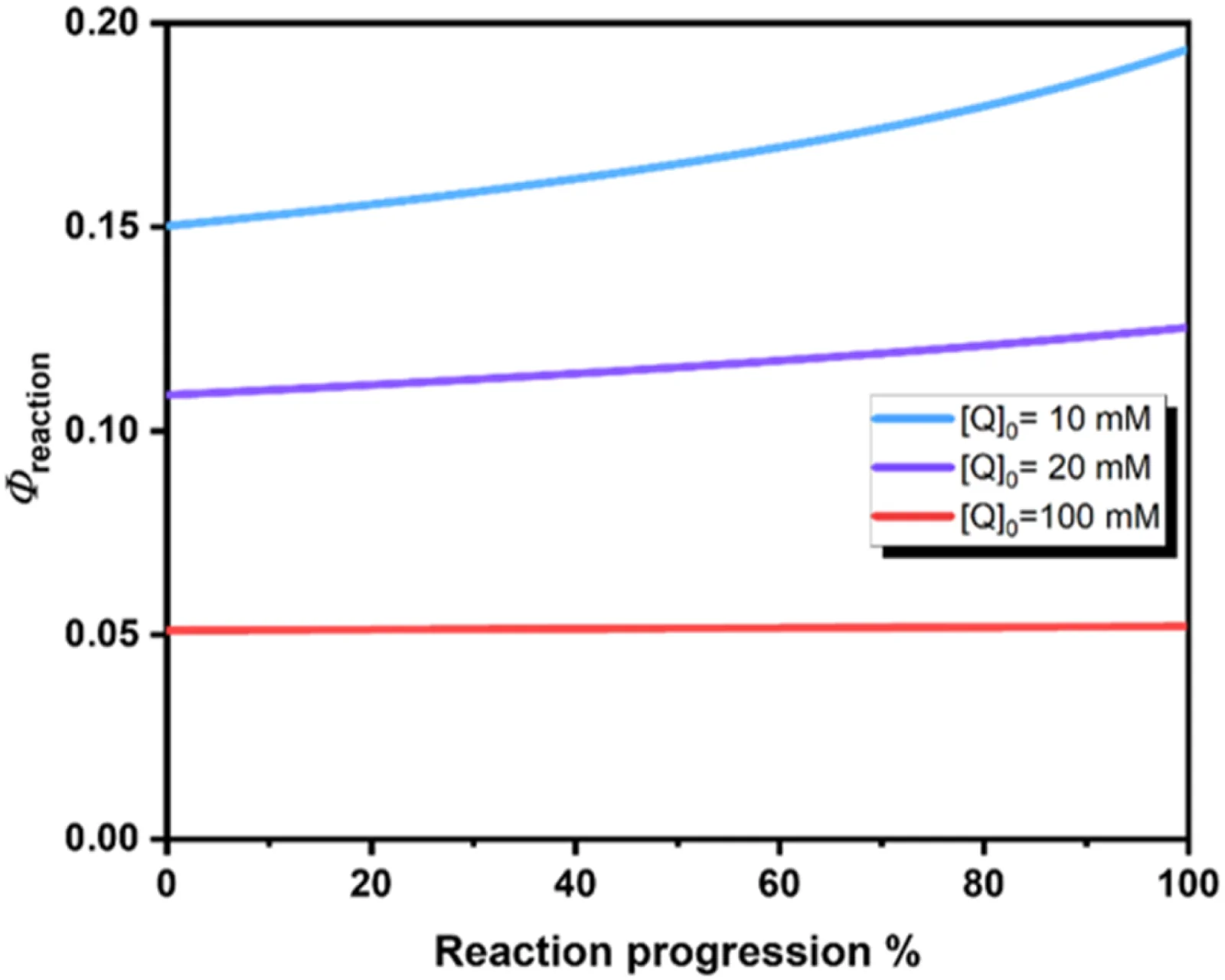
Predicted maximum photoreaction quantum yield (based on the initial radical yield following quenching of excited photocatalyst) *vs.* reaction progress with three initial quencher concentrations. The *x*-axis is normalized to the total reaction time to allow direct comparison.

• Optimal case (10 mM): the reaction initially proceeds within an intermediate regime between the singlet and triplet states reactivity. As the quencher is progressively depleted, the system increasingly populates the “triplet domain”, accompanied by an increase in quantum yield, reflecting the system's approach to the region of maximal efficiency.

• Higher-concentration cases (20 and 100 mM): these reactions start in a regime dominated by the unproductive singlet quenching channel, resulting in low initial efficiency. As the quencher is gradually consumed and its concentration decreases, the system begins to shift toward the more productive triplet manifold. However, the increase in reaction quantum yield is less pronounced than in the low-quencher-concentration case, because the (relative) quencher depletion is minimal and the system remains in a region where singlet quenching is dominant.

Noteworthily, the *x*-axis in Fig. 5 was normalized by replacing absolute time with reaction progress, expressed as the percentage of substrate conversion (0–100%). This approach was necessary as different initial quencher concentrations, generating different quantum yields result in drastically different times to reach 99% substrate conversion. Importantly, these two aspects are intimately connected: higher reaction efficiency accelerates substrate consumption, effectively shortening the reaction time, reflecting the system's ability to utilize photon energy more effectively. Conversely, when the reaction is less efficient, a significant fraction of excitation is unproductive, and the photocatalyst must undergo multiple re-excitation cycles to drive the reaction to completion. This not only prolongs the effective reaction time but also increases the likelihood of side reactions or degradation processes, particularly if the system or catalyst becomes unstable over extended irradiation. Thus, the mutual relationship between efficiency and reaction duration may influence both the productivity and the overall robustness of the photoreaction.

Similar considerations regarding the effect of quencher concentration also apply to systems in which the reaction substrate itself acts as the quencher in the initial electron transfer step,^71^ where operating under dilute substrate conditions would provide an efficiency advantage for the reaction. The corresponding simulation plot is provided in Section 2.9 of the SI (mechanistic model b).

### Mathematical treatment of TADF kinetics and validity of the simplified non-TADF approximation

It should be noted that eqn (2) and (3) employ a simplified kinetic framework that approximates the TADF photocatalyst as a conventional non-TADF one. However, this approximation fails to accurately describe the bidirectional population flux between S_1_ and T_1_ through risc and isc, respectively.^72,73^ In this perspective, quenching interactions may occur not only during the initial population, but also after the excited state has transitorily been repopulated through isc–risc cycles. To ensure that this approximation does not compromise the quantitative accuracy of our study, we validated the results against exact TADF kinetic expressions. This formalism includes a specific recursion factor to account for the continuous repopulation of the excited states. As detailed in Section 2.8 of the SI, the state-specific cage escape efficiencies (*η*_ce,S_ and *η*_ce,T_) derived from both treatments are the same. This demonstrates that the dominant radical-forming quenching events occur immediately following the first population of each excited state, which might usually be the case under application-related conditions, *i.e.* at much higher quencher and substrate concentrations compared to frequently used diluted solutions for spectroscopy. Consequently, the simplified model applied to the treatment of data from transient absorption spectroscopy remains a robust and precise methodology for predicting catalytic performance in these systems. Secondary repopulation effects, which are indeed the peculiar characteristic of TADF photocatalysts, do not play a decisive role in the initial step of the catalytic cycle (at least for the systems we investigated).

### “Less is more” effect with additional photocatalysts possessing dual singlet and triplet photochemistry

In addition to the TADF emitter that was employed for our initial experiments showing the “less is more” concept, many organic photocatalysts^8,37,74^ commonly used for photoinduced electron transfer reactions have a photoactive singlet and a triplet excited state. For those chromophores, triplet formation is often incomplete (*Φ*_isc_ ≪ 1) and occurs on a timescale of a few nanoseconds such that both excited states, S_1_ and T_1_, can contribute to the overall reaction progress.^1^ In principle, the concept should therefore be relevant for a variety of photocatalysts with dual singlet and triplet reactivity in electron transfer reactions. To test the broader applicability of our concept, we investigated two additional systems.

Firstly, we selected an additional TADF compound, the well-known 4CzIPN in toluene, as a photocatalyst and the aromatic amine DMA as a reversible reductive quencher (see Fig. 6A for structures and key properties). In toluene, *Φ*_isc_ for 4CzIPN is much higher compared to MeCN,^75^ theoretically enabling more pronounced concentration-dependent effects. The quencher was chosen to prohibit unwanted side reactions known for aliphatic amines,^39,55,56,76,77^ allowing us to quantify the radical yields in a reliable manner. Secondly, a photocatalyst that is soluble in water, SO_2_TDS^2−^, was combined with the ascorbate monoanion as a reductive quencher (see Fig. 6B for structures and key properties).^74^ The spectral signatures of the radical species after electron transfer are known for both photocatalysts^74,77^ and we were able to reproduce those spectra in our measurements (see Section S3 for details). The relative intensities of the radical species found in transient absorption spectroscopy at different concentrations of the respective reductive quencher are directly connected to the corresponding cage escape quantum yields (Fig. 6C). In good agreement with the detailed investigation of PC^2+^ shown above, we observed for both systems clearly a “less is more” effect. Instead of the laborious determination of absolute cage escape efficiencies for singlet and triplet quenching that rely on carefully calibrated transient spectra in the respective solvent, the (relative) ratio of both cage escape efficiencies can be extracted from the plot shown in Fig. 6C by applying eqn (2)–(4). This plot also demonstrates that the “less is more” effect is most pronounced for a higher *η*_ce,T_/*η*_ce,S_ ratio in combination with a high initial isc efficiency (11.5/1 for SO_2_TDS^2−^ and 125/1 for 4CzIPN), since the relative radical concentration drops almost to zero for 4CzIPN at high concentrations of DMA. Consequently, these measurements would predict a decrease of the overall reaction quantum yield by one order of magnitude when the quencher (DMA in this case) concentration is increased from 10 mM to 200 mM. Circumventing too high quencher concentrations by either dilution of the whole system or by avoiding the usage of a large quencher excess – which would be predicted by exclusively considering conventional emission-based Stern–Volmer experiments – is regarded to be highly important for maximizing the reaction quantum yields. Ultimately, the “less is more” effect was observed for the pinacol coupling of acetophenone (15.7 mM) using SO_2_TDS^2−^ as photocatalyst and HAsc^−^ as reductive quencher under 390 nm LED irradiation (see Section S3.2 in the SI). The performance of the reaction was ∼50% higher using lower HAsc^−^ concentrations (19.2 mM) compared to a larger excess of the reductive quencher (96.9 mM), which is in very good agreement with the relative SO_2_TDS˙^3−^ yield determined spectroscopically (see Fig. 6C).

**Fig. 6:**
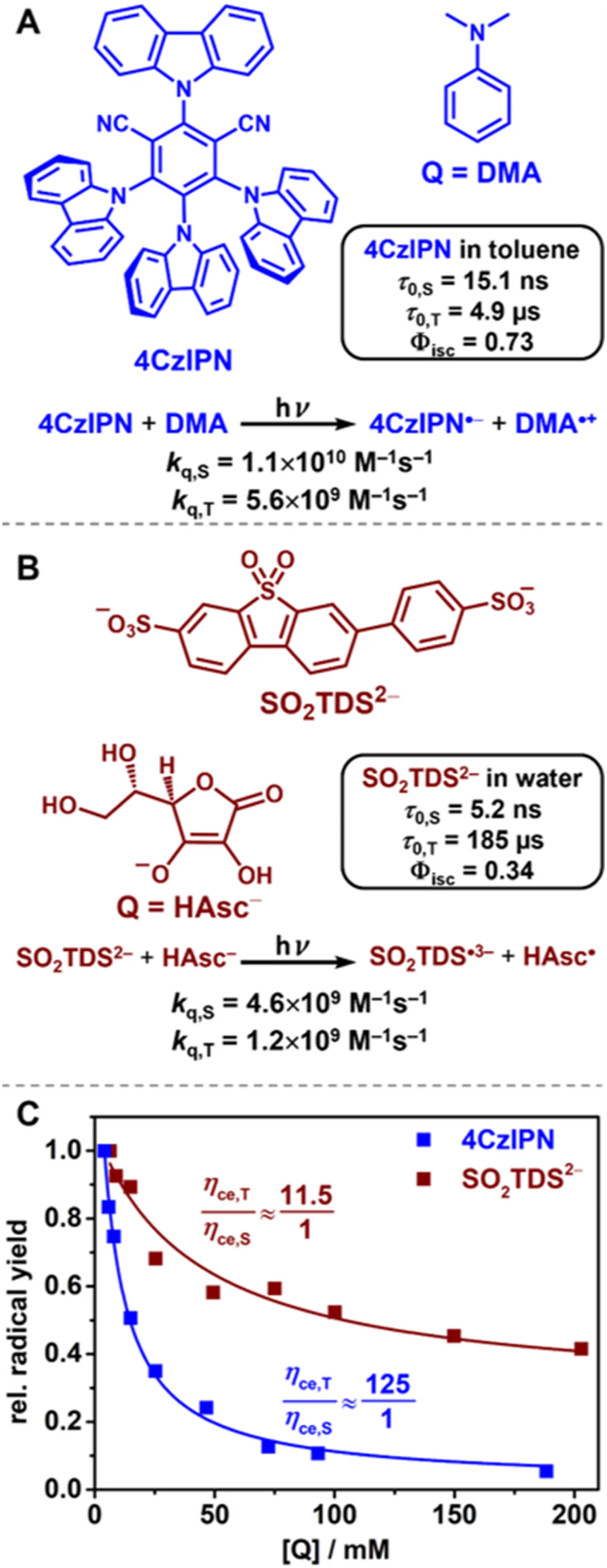
(A) Chemical structures of the TADF photocatalyst 4CzIPN and the reductive quencher DMA as well as key photophysical properties of 4CzIPN in toluene (*Φ*_isc_ from ref. 75) and quenching rate constants for S_1_ and T_1_ quenching by DMA. (B) Chemical structures of the non-TADF organic photocatalyst SO_2_TDS^2−^ and the reductive quencher HAsc^−^ as well as key photophysical properties of SO_2_TDS^2−^ in water (*Φ*_isc_ from ref. 74) and quenching rate constants for S_1_ and T_1_ quenching by HAsc^−^. (C) Quencher concentration-dependent relative radical yields measured *via* transient absorption spectroscopy of the systems introduced in (A) and (B) using the same color code along with a fitting function combining eqn (2)–(4) to determine the relative cage escape efficiencies of T_1_ and S_1_ quenching (see text and SI for further information).

## Conclusions

This study provides unambiguous spectroscopic proof for the “less is more” effect in organic photoredox reactions employing catalysts with dual singlet and triplet reactivity. Our results demonstrate that a higher quenching efficiency is often an unreliable proxy for catalytic productivity, as cage escape efficiency is the actual bottleneck for improvement. While previous cage escape efficiencies comparing singlet and triplet reactivity were deduced through the indirect kinetic fitting of final reduction products,^36^ our approach utilizes transient absorption spectroscopy to achieve direct quantification of radical intermediates formed upon quenching in such systems. In the TADF photocatalysts and the example for a conventional organic catalyst that we selected, the triplet manifold proved to be significantly more productive than the singlet channel for four different photocatalyst-quencher couples, a phenomenon dictated by the spin-forbidden nature for charge recombination from the radical pair in the triplet manifold. Lower quencher concentrations therefore prevent the quenching of the singlet state, allowing efficient intersystem crossing to the more productive triplet manifold. Furthermore, our validation of the “non-TADF approximation” establishes a theoretical bridge between TADF and conventional catalysts. By demonstrating that simplified models remain robust even when compared to exact kinetic treatments, incorporating S_1_–T_1_ cycling factors, we prove that the triplet manifold's superiority is an immediate consequence of spin-forbidden charge recombination rather than a consequence of excited-state recycling. Consequently, these results unify TADF and non-TADF photocatalysis under a single operational framework, identifying the optimization of quencher concentrations to favor the triplet pathway as a universal strategy for maximizing photon utilization efficiency. This universal nature is further reinforced by the parallel trends recently observed in non-TADF catalysts,^78^ where triplet-driven cage escape similarly dominates the catalytic outcome. Finally, by translating these fundamental time-resolved data into operative continuous irradiation modeling, we physically demonstrate that low quencher concentrations are sufficient to maximize yields while minimizing irradiation times, energy wasting and, simultaneously, degradation risks.

## Author contributions

F. F., M. S. and M.-S. B. conducted the photophysical measurements; F. F. and C. Be. performed the time-resolved simulations; C. Bu. synthesized the photocatalyst under the supervision of M. M. H.; C. K. conceived the project. The manuscript was written through contributions of all authors under the supervision of P. C. and C. K.; all authors have given approval to the final version of the manuscript.

## Conflicts of interest

There are no conflicts to declare.

## Supplementary Material

SC-OLF-D6SC06836G-s001

## Data Availability

The data that support the findings of this study are available in the main article and/or the supplementary information (SI). The data sets shown in the main paper are accessible *via* the JGU library (Gutenberg Open Science) and the homepage of the corresponding author (after final publication of the paper). Supplementary information: materials and methods, synthetic procedures and characterization data, details on photophysical measurements, kinetic modelling. See DOI: https://doi.org/10.1039/d6sc06836g.
