# Supplementary material for: When less is more: harnessing optimal quencher concentrations to maximize quantum yields in TADF photoredox reactions
Source: Chem Sci. 2026 Sep 28. Online ahead of print. doi: 10.1039/d6sc06836g (PMC13643228; doi:10.1039/d6sc06836g)
Supplement: Supplementary file 1 [file SC-OLF-D6SC06836G-s001.pdf]

Supplementary Information (SI) for Chemical Science.
This journal is © The Royal Society of Chemistry 2026
Supplementary Information (SI) for Chemical Science.
This journal is © The Royal Society of Chemistry

Electronic Supplementary Information

When less is more: harnessing optimal quencher concentrations to maximize quantum yields in TADF photoredox reactions

Federica Fina [a, b], Matthias Schmitz [a], Maria-Sophie Bertrams [a], Caterina Bellatreccia [b], Chris Burdenski [c], Max M. Hansmann [c], Paola Ceroni [b*] and Christoph Kerzig [a*]

[a] Department of Chemistry, Johannes Gutenberg University Mainz, 55128 Mainz, Germany.
[b] Alma Mater Studiorum-Dipartimento di Chimica "G. Ciamician", Università di Bologna, Via Gobetti 85, Bologna 40129, Italy.
[c] Fakultät für Chemie und Chemische Biologie, Technische Universität Dortmund, Otto-Hahn-Str. 6, 44227 Dortmund, Germany.

*Email: ckerzig@uni-mainz.de; paola.ceroni@unibo.it

Table of contents

S1 Materials and Methods ... 2
Photophysical Studies with PC^{2+}, 4CzIPN and SO_2TDS^{2-} as well as irradiation experiments (University of Mainz) ... 2
Synthesis and Characterizations of PC^{2+} (University of Dortmund) ... 3
S2 Photophysical Studies of PC^{2+} ... 4
S2.1 Determination of $\Delta\varepsilon$ of $PC^{\bullet+}$ ... 4
S2.2 Determination of Transient Absorption Spectrum of $^{3*}PC^{2+}$ ... 7
S2.3 Quenching Experiments ... 9
S2.4 Determination of Quantum Yield of Free Radical Formation $\Phi_{r,tot}$ ... 12
Quencher Concentration – Dependent $\Phi_{r,tot}$: 4-*tert*-butylphenol ... 12
Quencher Concentration – Dependent $\Phi_{r,tot}$: naphthalene ... 15
S2.5 Determination of Cage Escape Efficiency, η_{ce} ... 17
General Procedure: Cage Escape Efficiency upon Quenching of T_1, $\eta_{ce,T}$ ... 17
General procedure: Cage Escape Efficiency upon Quenching of S_1, $\eta_{ce,S}$ ... 18
S2.6 Experiment Series 1: Quencher 4-*tert*-butylphenol ... 19
Serie 1a: Determination of $\eta_{ce,T}$ ... 19
Series 1b: Determination of $\eta_{ce,S}$ ... 21
S2.7 Experiment Series 2: Quencher Naphthalene ... 23

Series 2a: Determination of $\eta_{ce,T}$ 23

Series 2b: Determination of $\eta_{ce,S}$ 24

S2.8 Kinetic Parameters and η_{ce} with Exact TADF Formalism........ 26

S2.9 Transferring Spectroscopic predictions to continuous irradiation (University of Bologna)........ 29

Mechanistic Model *a* 29

Mechanistic Model *b* 32

S3. “Less is More” Effect for Further Photocatalysts 34

S3.1 4CzIPN 34

S3.2 SO_2TDS^{2-} 36

S4 Assessing the Sensitivity of Kinetic Parameters in Model TADF Photocatalytic Systems... 41

S5 Synthetic Procedures 44

References 56

S1 Materials and Methods

Photophysical Studies with PC^{2+}, 4CzIPN and SO_2TDS^{2-} as well as irradiation experiments (University of Mainz)

Acetonitrile, 99.9%, VWR Chemicals; acetophenone, >99%, Sigma-Aldrich; biphenyl, ≥99%, Sigma-Aldrich; deuterium oxide (D_2O), 99.90%, Deutero; *N*,*N*-dimethylaniline, 99%, Sigma Aldrich; naphthalene, 99.6%, Alfa Aesar; sodium acetate (NaOAc), >99%, Sigma-Aldrich; sodium L-ascorbate, 98%, Sigma-Aldrich; sodium phosphate dibasic, >99%, Sigma Aldrich; sodium phosphate monobasic monohydrate, >99%, Sigma Aldrich; 4-*tert*-butylphenol, 97%, Acros Organics; toluene, ≥99.8%, Fisher scientific. Aqueous solutions were prepared using ultrapure Millipore MilliQ water with a specific resistance of 18.2 MΩ cm (20 °C). 4CzIPN and SO_2TDS^{2-} were available from former projects.[1,2]

Absorption spectra were recorded using a *Perkin Elmer* LAMBDA 365 instrument.

Transient absorption spectra or time-resolved absorption traces were recorded with an LP980KS setup from *Edinburgh Instruments* with an Nd:YAG laser from *Quantel* (Q-smart 450) with frequency-tripled output (355 nm). The laser pulse duration was about 5 ns and the pulse frequency was 10 Hz. The excitation energy was adjusted by modifying the Q-switch delay. Pulse energy measurements were conducted using a pyroelectric detector equipped with a broadband absorber sourced from gentec-eo (QE25LPS-MB QED-D0). Transient absorption spectra were detected using an iCCD camera from *Andor* and were integrated over 100 ns. Single-wavelength kinetics were recorded using a *Hamamatsu*

photomultiplier tube (R928). Spectroscopic experiments were performed using a temperature-controlled cell holder at 20 °C.

TCSPC (time-correlated single photon counting) measurements for PC^{2+} were performed using a Mini-Tau spectrometer from *Edinburgh Instruments*, with an EPL-450 (λ_{exc} = 446 nm, pulse width: 74.5 ps) or an EPL-375 (λ_{exc} = 371 nm, pulse width: 58.3 ps) for 4CzIPN as excitation source from the same manufacturer. The detection window was defined using the instrument's built-in bandpass filter. The internal response function (IRF) was determined by measuring stray light with a diluted ludox solution.

TCSPC measurements for 4CzIPN and SO_2TDS^{2-} were conducted with the FS5 Spectrofluorometer from *Edinburgh Instruments* equipped with a R13456 photomultiplier tube from *Hamamatsu* and a temperature-controlled cuvette holder (SC-25, T = 20 °C). The pulsed laser EPL-375 (λ_{exc} = 371 nm, pulse width: 58.3 ps) from *Edinburgh Instruments* was used as excitation source. The IRF was determined by measuring stray light with a diluted ludox solution.

Irradiation experiments were carried out using SO_2TDS^{2-} as photocatalyst in the solvent D_2O. A RP160L 390 nm Kessil LED was used as the light source. The sample solution was prepared under an Ar atmosphere and transferred to a septum-sealed NMR tube. During irradiation, the NMR tube was connected to an Ar line and placed inside a water bath. Furthermore, the LED was placed as close as possible to the NMR tube (see reference 2 for the experimental setup). The yield was determined by ^{1}H NMR spectroscopy using NaOAc as internal reference.

NMR spectroscopic studies were performed with an Avance III HD 300 MHz or an Avance II 400 MHz multi-nuclear magnetic resonance spectrometer from *Bruker* at 22 °C. The ^{1}H NMR spectra were referenced using the residual non-deuterated solvent. Chemical shifts are given as δ values in ppm.

Synthesis and Characterizations of PC^{2+} (University of Dortmund)

All solvents were purified with a *MBraun* SPS – 800 over molecular sieves and degassed with argon. C_6D_6 was distilled over Na and degassed. Solids were handled and NMR samples were prepared in a nitrogen filled glovebox. High resolution MS (EI): Finnigan MAT 8200 (70 eV), ESIMS: Finnigan MAT 95, accurate mass determinations: *Bruker* APEX III FT-MS (7 T magnet) and LTQ-Orbitrap-XL (Thermo Scientific) equipped with a heated electrospray ionization source (HESI). NMR: NMR spectra were measured on the spectrometers *Bruker* AV 500 Avance NEO, Bruker AV 400 Avance III HD NanoBay, AV 600 Avance III HD and AV 700 Avance III HD and chemical shifts (δ) are referenced to their solvent signals [C_6D_6, 7.16 (^{1}H NMR) 128.06 (^{13}C NMR); CD_3CN, 1.94 (^{1}H NMR) 118.26 (^{13}C NMR), $CDCl_3$ 7.26 (^{1}H NMR) 77.16 (^{13}C NMR), coupling constants (J) in Hz. All spectra were recorded in 5 mm NMR tubes at the temperatures indicated. Flash chromatography was performed with Merck 60 silica gel (40-63 µm). Thin-layer chromatography (TLC) analysis was performed using Merck silica gel 60 F254 TLC plates and visualized by UV irradiation and ceric ammonium molybdate, $KMnO_4$, vanillin or p-anisaldehyde. All commercially available compounds (Acros, abcr, Alfa Aesar, BLD pharm, Carbolution,

ChemPur, Fluorochem, Sigma Aldrich, TCI) were used as received. Melting points were measured with a Büchi M-560 apparatus. IR-ATR measurements (diamond) were performed in reflection mode on a Bruker Alpha II inside a glovebox, wavenumbers in cm^{-1}.

S2 Photophysical Studies of PC^{2+}

S2.1 Determination of Δε of $PC^{•+}$

To determine the difference extinction coefficient of the reduced photocatalyst **$PC^{•+}$**, a photoinduced electron transfer approach was employed using biphenyl (**BP**) as a reference quencher. Upon 355 nm laser excitation, the excited photocatalyst undergoes electron transfer with **BP**, generating an equimolar ratio of **$PC^{•+}$** and the oxidized form of the quencher **$BP^{•+}$**, of which the molar absorption coefficient is known between 350 and 700 nm.[3]

According to a previous study,[4] **$PC^{•+}$** exhibits negligible absorbance at 385 nm; therefore, any change in absorbance at this wavelength is assumed to exclusively stem from **$BP^{•+}$**. The initial transient absorption value ΔA_0, where '0' denotes the initial (time-averaged) signal at each laser intensity that was determined by averaging the signal recorded in the 0.1–1.2 µs time window following the laser pulse (Fig. S1). Within this interval, the concentration of **$BP^{•+}$** is assumed to remain constant due to its slow decay on the microsecond-to-millisecond timescale. The lower bound of 0.1 µs was chosen to exclude the prompt instrument response, including scattered laser light and any residual direct emission, which could otherwise distort the early-time transient absorption signal. The molar absorptivity (ε) of **$BP^{•+}$** is reported in the literature at 383 nm; in this work, the value at 385 nm, which corresponds to the maximum of the absorption band recorded with our setup, was used, as derived from the corresponding absorption spectrum, giving $\Delta\varepsilon_{385\,\mathrm{nm}} = 27000\ M^{-1}\,cm^{-1}$. Based on this, the concentration of **$BP^{•+}$** formed immediately after the photoinduced electron transfer process was calculated at each laser intensity using the Beer–Lambert law for 1 cm pathlength cuvettes:

$$[\mathrm{BP}^{\bullet+}] = \frac{\Delta A_{0,385\ \mathrm{nm},n}}{\Delta\varepsilon_{385\ \mathrm{nm},n}\ \times 1\ cm}$$

For the determination of the difference extinction coefficient of **$PC^{•+}$**, the ΔA_0values at each laser intensity were obtained by averaging the transient absorption signal in the 0.5–2.2 µs time window at 450 nm, a spectral region with no contribution from **$BP^{•+}$** (Fig. S2).

Since identical cuvettes were used and the laser as well es detection pathways were unmodified, the optical path length remained constant. Therefore, $\Delta\varepsilon_{450\,\mathrm{nm}}$ (**$PC^{•+}$**) could be determined by relating the absorbance changes to the concentration of **$PC^{•+}$**, which is equal to that of **$BP^{•+}$**.

$$\Delta\varepsilon_{450\ \mathrm{nm},n}(\mathrm{PC}^{\bullet+}) = \frac{\Delta A_{0,450\ \mathrm{nm}}}{[\mathrm{PC}^{\bullet+}]}$$

with $[\mathrm{PC}^{\bullet+}] = [\mathrm{BP}^{\bullet+}]$

The procedure was repeated on the same solution at increasing laser intensities to obtain an averaged value from five independent measurements.

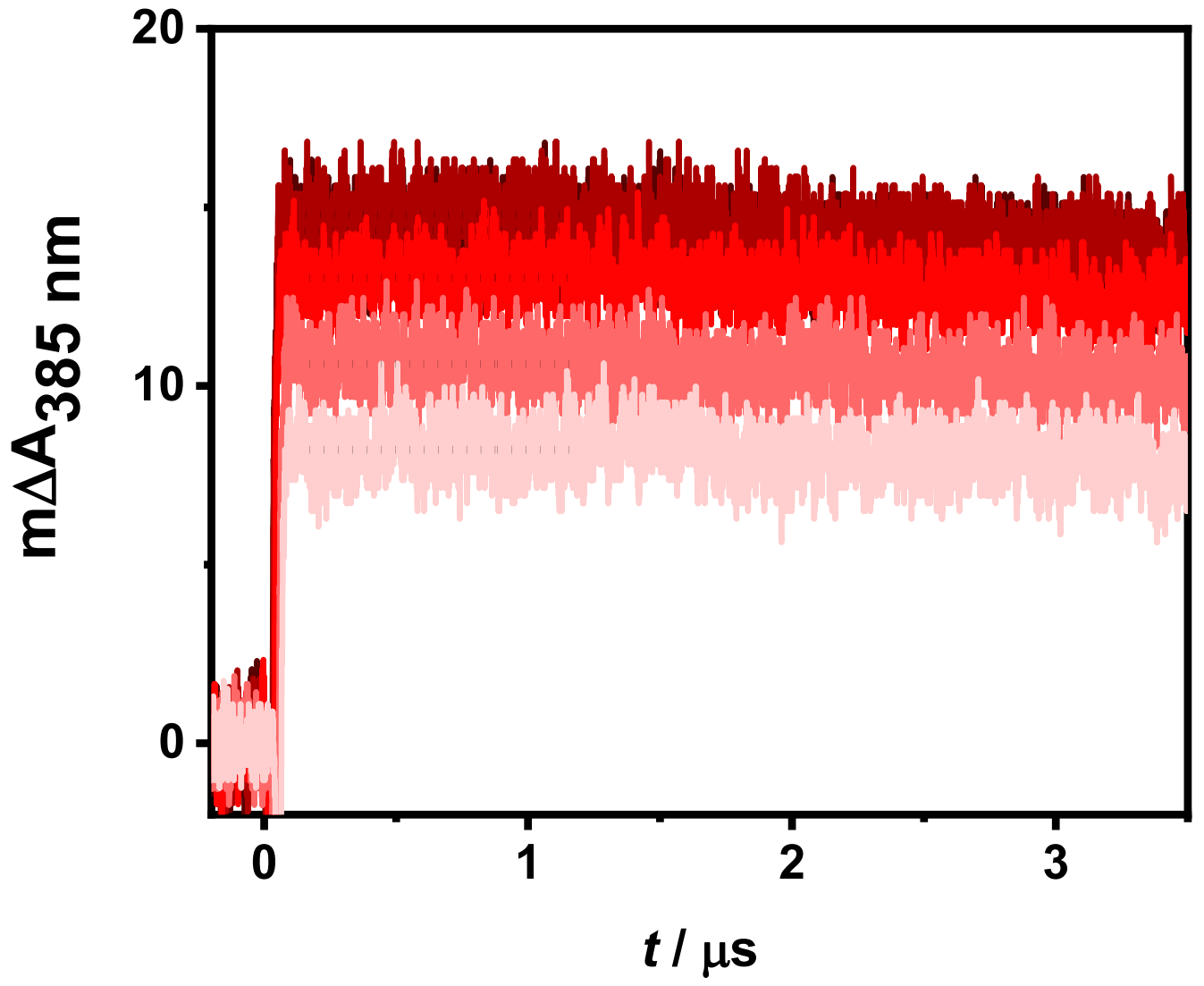

Fig. S1. Time-resolved transient absorption traces at 385 nm after laser excitation of Ar-saturated acetonitrile solutions containing **5 µM** of **PC**$^{2+}$ with **197 mM** of **BP** after laser excitation (λ_{exc} = 355 nm) with increasing laser pulse intensities (5.1-13.7 mJ/pulse). The absorption is attributed to **BP**$^{\bullet+}$ formed upon photoinduced electron transfer. The dotted lines indicate the time interval for data averaging to determine ΔA_0 value**.**

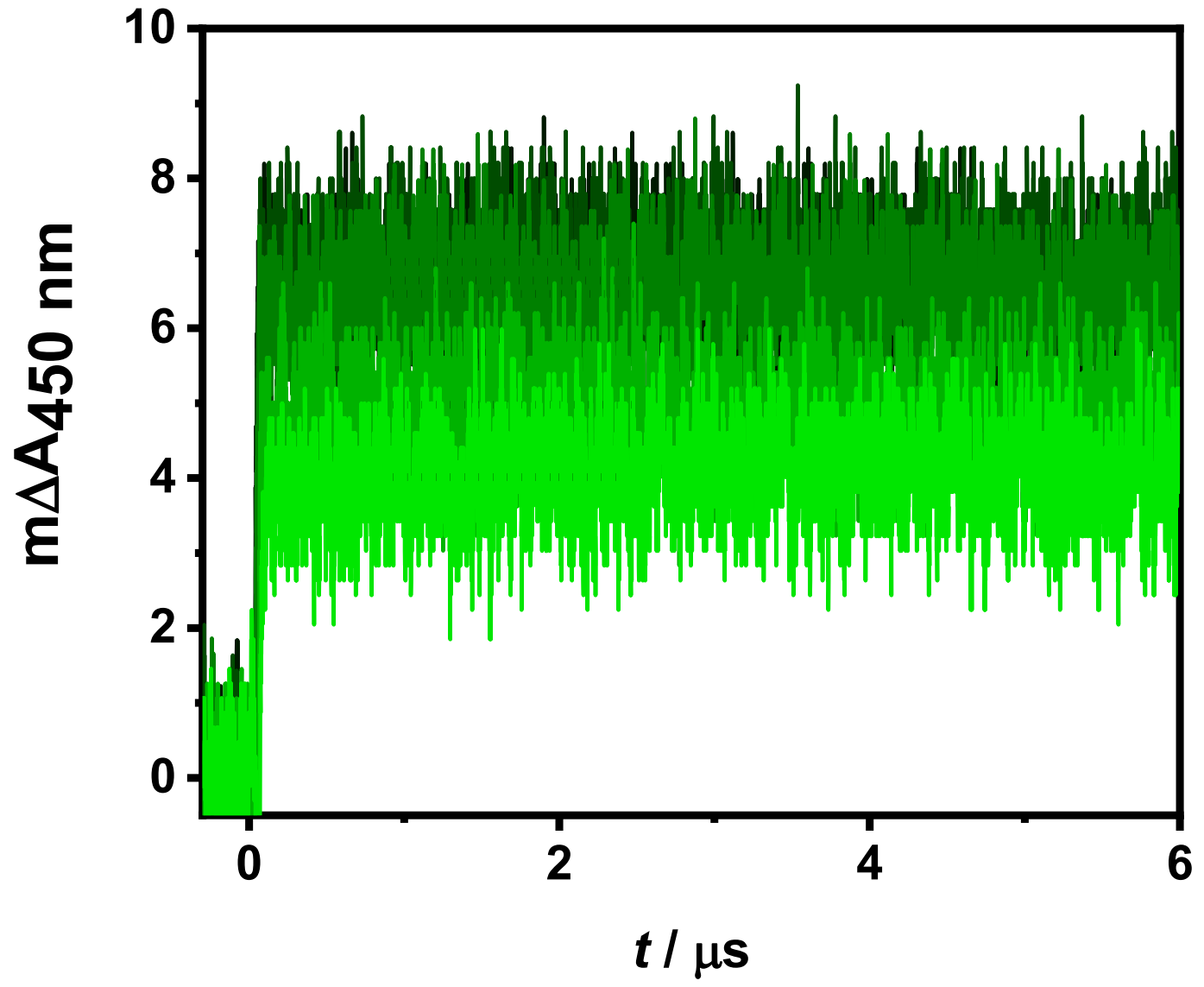

Fig. S2. Time-resolved transient absorption traces at 450 nm after laser excitation of Ar-saturated acetonitrile solutions containing **5 µM** of **PC**$^{2+}$ with **197 mM** of **BP** after laser excitation (λ_{exc} = 355 nm) with increasing laser pulse intensities (5.1-13.7 mJ/pulse). The absorption is attributed to **PC**$^{\bullet+}$ formed upon photoinduced electron transfer. The dotted lines indicate the time interval for data averaging to determine ΔA_0 value.

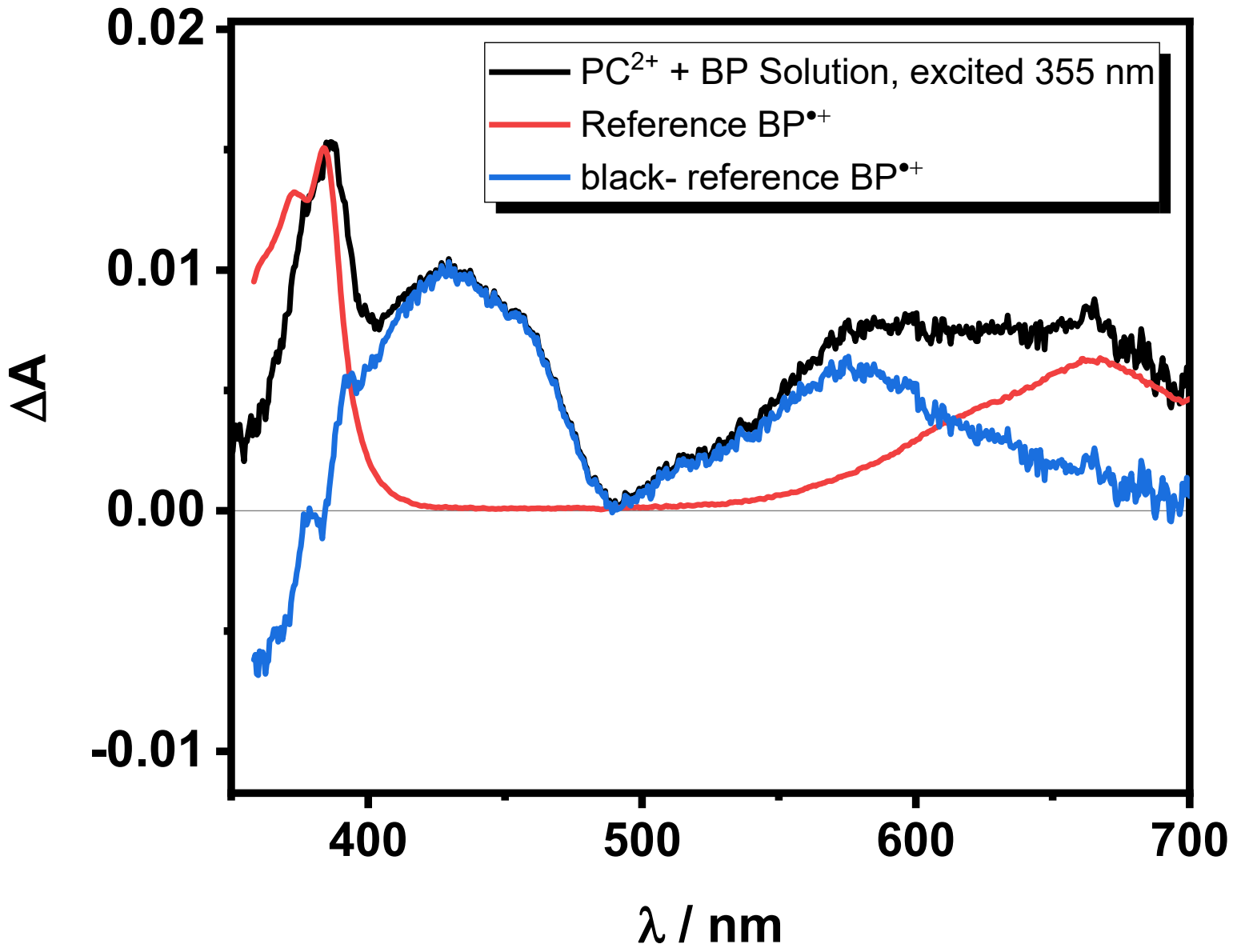

Fig. S3. Black line: transient absorption spectrum of a solution containing **5 μM** of $\mathbf{PC^{2+}}$ and **197 mM** of **BP** recorded with 1 μs delay after 355 nm laser excitation (15 mJ/pulse); red line: reference spectrum for $\mathbf{BP^{\bullet+}}$, scaled at 385 nm; Blue line: difference spectrum obtained by subtracting the red line to the black line, highlighting the contribution of $\mathbf{PC^{\bullet+}}$ to the overall absorption.

The difference molar absorption coefficient of $\mathbf{PC^{\bullet+}}$ at 450 nm was determined by averaging entries 1-5 (Table S1):

$$\Delta\varepsilon_{450\ nm} = 13600 \pm 700\ M^{-1}\ cm^{-1}$$

The absorption spectrum was calculated accordingly.

Table S1. Experimental data for measurements performed to determine the differential molar absorption coefficient of $\mathbf{PC^{\bullet+}}$ at 450 nm.

**Entry / *n***	**Laser intensity/ mJ/pulse**	**m$\Delta A_{0,\ 385\ nm}$ ($BP^{\bullet+}$)**	**[BP^{+}] = [$PC^{\bullet+}$] / M**	**m$\Delta A_{0,\ 450\ nm}$ ($PC^{\bullet+}$)**	**$\Delta\varepsilon_{450\ nm}$ ($PC^{\bullet+}$) / $M^{-1}\ cm^{-1}$**
**1**	5.1	8.21	3.0×10^{-7}	4.01	13281
**2**	7.0	10.30	3.8×10^{-7}	4.98	13141
**3**	9.9	10.63	3.9×10^{-7}	6.46	16524
**4**	12.4	14.35	5.3×10^{-7}	6.64	12581
**5**	13.7	14.80	5.4×10^{-7}	6.88	12640

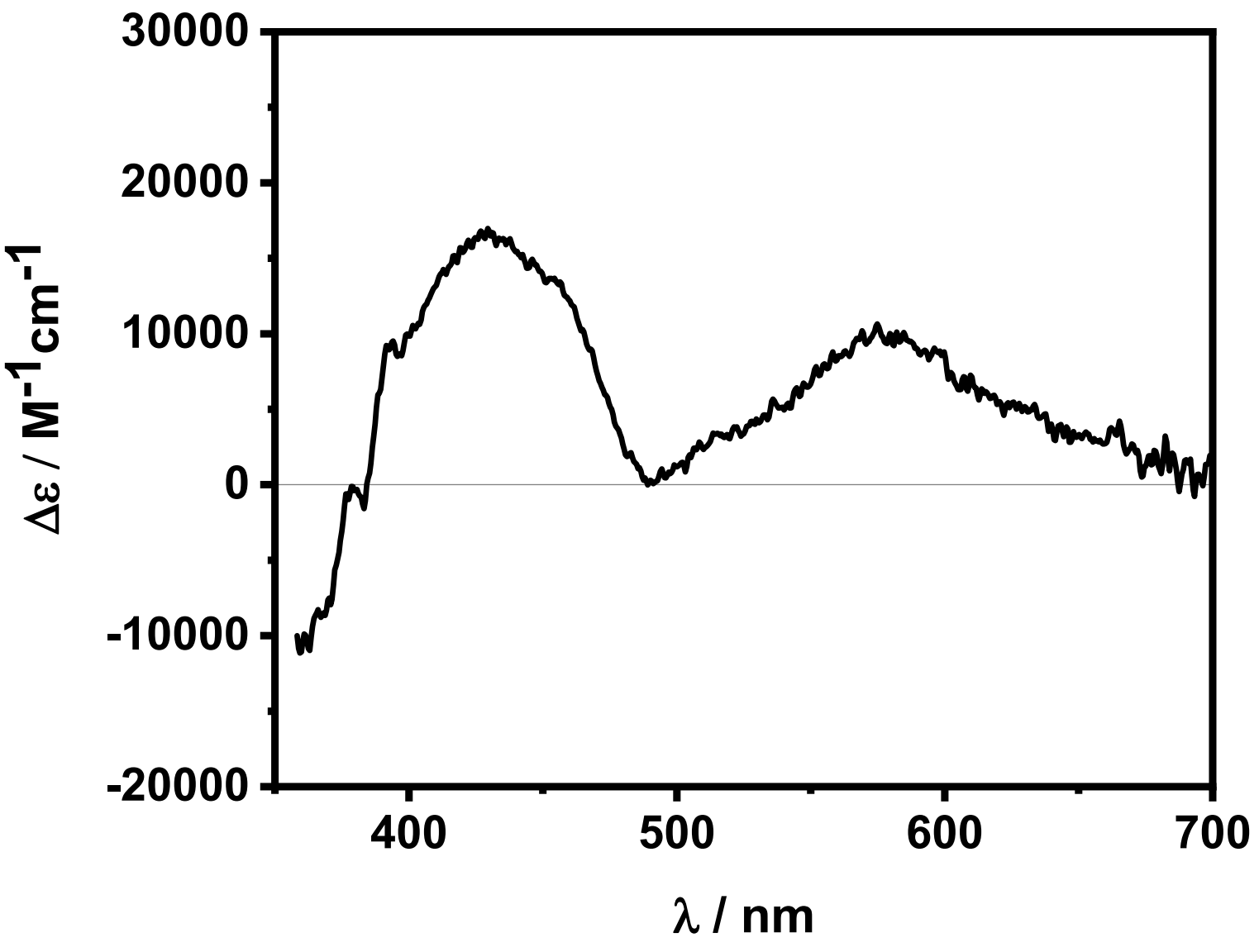

Fig. S4. Calibrated transient absorption spectrum of **$PC^{\bullet+}$** in acetonitrile.

S2.2 Determination of Transient Absorption Spectrum of $^{3*}PC^{2+}$

The difference transient absorption spectrum of the triplet excited state of the photocatalyst, **$^{3*}PC^{2+}$**, was determined from the transient absorption trace at 405 nm (Fig. S5) of a solution containing 5 µM of **PC^{2+}** and 0.22 mM of **4-tbuPhOH** (quenching experiments with 4-tbuPhOH are described in section S2.3). The time resolved absorption trace exhibits a biexponential decay, due to the decay of the triplet state (short component) resulting in the formation of **$PC^{\bullet+}$** (long-lived component) upon photoinduced electron transfer, decaying on a time scale exceeding the detection window. The absorption decay was fitted with a biexponential function and extrapolated to $t = 0$, yielding triplet-state absorption immediately after the laser pulse: ΔA_0 = 0.037. Under the employed concentration conditions, singlet-state quenching was low (≤ 0.02) and the intersystem crossing quantum yield was almost maximal (Φ_{isc}=0.66). The concentration of excited states formed immediately after 355 nm excitation was determined by actinometry using an iso-absorbing (at the excitation wavelength) aqueous solution of $[Ru(bpy)_3]Cl_2$ (Fig. S6), yielding a value of [**$^{*}PC$**] = 4.6×10^{-6} M (detailed explanation of actinometric measurements in section S2.4). This value was corrected by multiplying for Φ_{isc} to obtain [**$^{3*}PC^{2+}$**] = 3.0×10^{-6} M, which was then used to calibrate the differential absorption spectrum of **$^{3*}PC^{2+}$**.

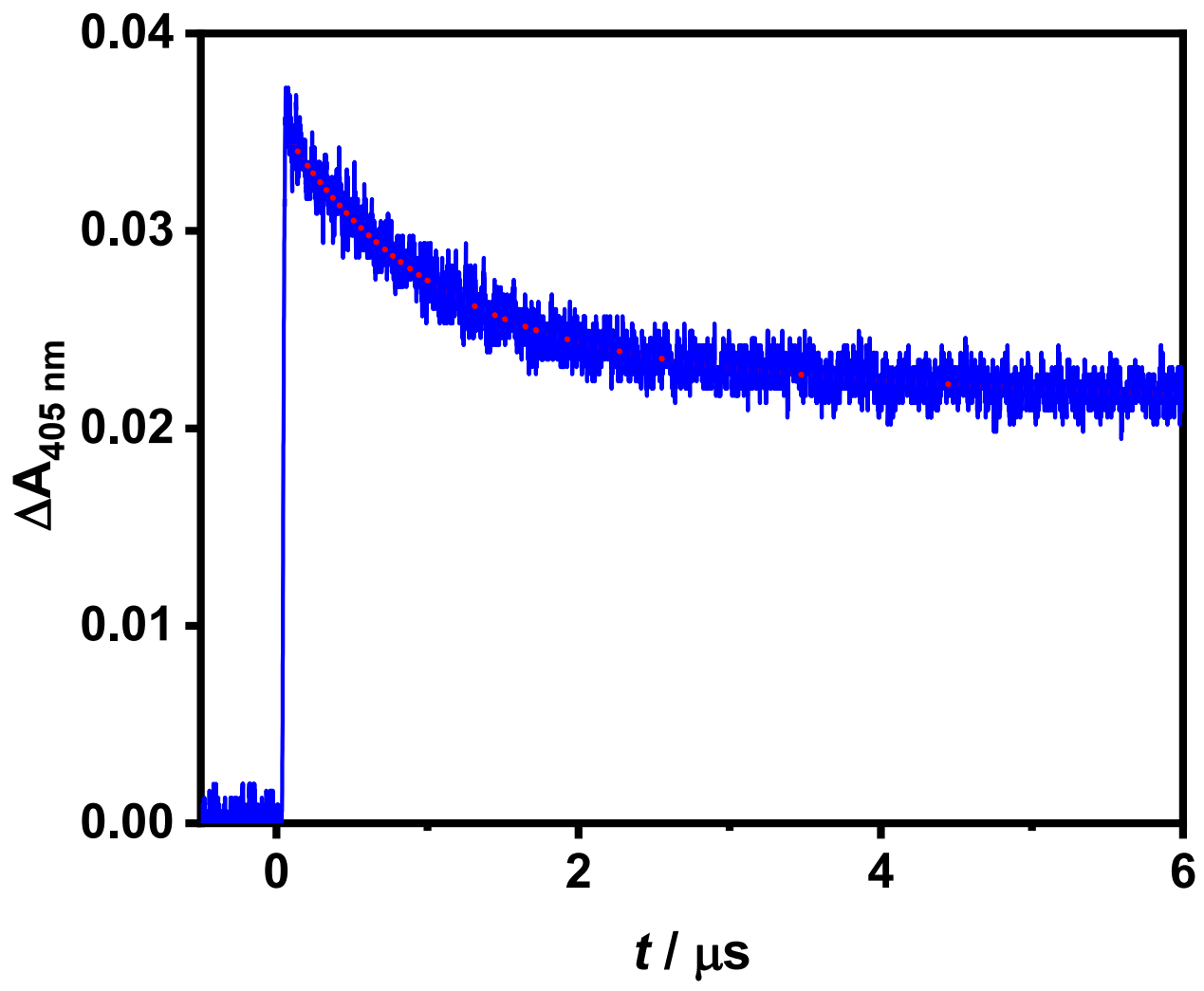

Fig. S5. Time-resolved transient absorption traces after 355 nm laser excitation of Ar-saturated acetonitrile solutions containing **5 µM** of **PC^{2+}** and 0.22 mM of **4-tbuPhOH** after laser excitation (λ_{exc} = 355 nm, 5.6 mJ/pulse, $A_{355\ nm}$ = 0.2). The dotted lines indicate the biexponential fit used to extrapolate ΔA_0.

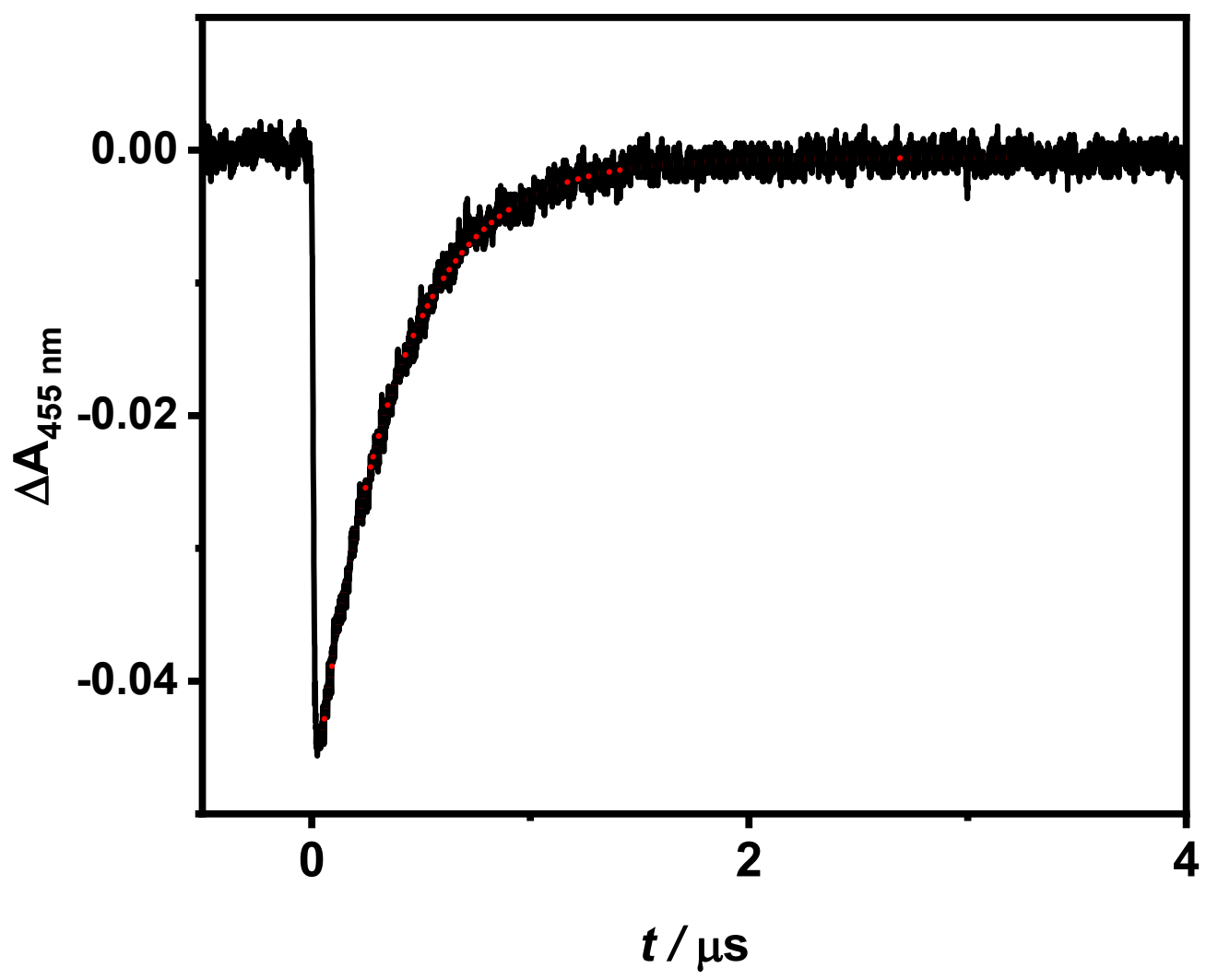

Fig. S6. Time-resolved transient absorption traces following laser excitation (λ_{exc} = 355 nm, 5.6 mJ/ pulse, $A_{355\ nm}$ = 0.2) of air-saturated aqueous solutions of **$[Ru(bpy)_3]Cl_2$**. The concentration was adjusted to match the ground state absorbance of 0.2 at 355 nm of the PC^{2+} / 4-tbuPhOH solution. The signal at 455 nm corresponds to the ground-state bleach and exponential fitting (dotted lines) of the decay traces was used to extrapolate the ΔA_0 in order to calculate the concentration of excited states immediately after laser excitation.

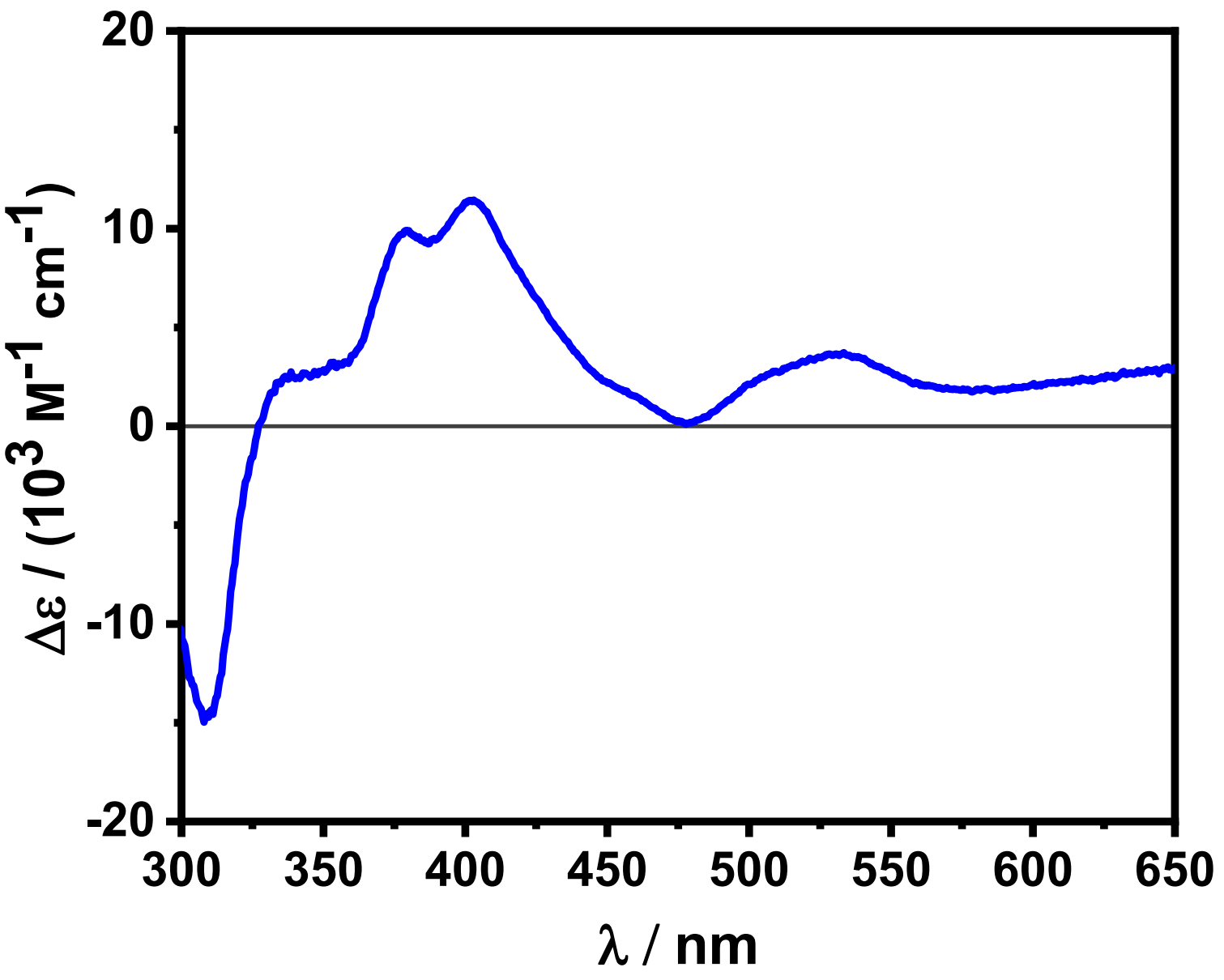

Fig. S7. Calibrated transient absorption spectrum for ^{3*}**PC^{2+}** in acetonitrile.

S2.3 Quenching Experiments

For S_1 state quenching, measurements were carried out by using time-correlated single photon counting (TCSPC), monitoring the time-resolved decay of prompt fluorescence at increasing quencher concentrations in air-equilibrated acetonitrile solutions. The emission spectrum of **PC$^{•+}$** is known from previous work.[4] Excitation was set at λ_{exc} = 446 nm, and emission was collected in the 525–575 nm range, using a laser repetition rate of 200 kHz. The resulting lifetimes were analyzed according to the Stern–Volmer equation to extract the quenching constants.

For T_1 quenching, experiments were conducted in argon-saturated acetonitrile solutions, obtained by bubbling argon for 20 minutes. The decay of the transient signal at 405 nm, attributed to triplet-state absorption, was similarly analyzed using the Stern–Volmer equation to determine the corresponding quenching constants. The long lifetime of ^{3*}**PC^{2+}** in the µs range is highly susceptible to minor changes of the solution or experimental conditions such as laser intensity, traces of oxygen or even the way the cuvettes were cleaned prior to the preparation of fresh samples. This is most likely a result of the interplay of the long lifetime and the extremely high photoredox reactivity. For each set of measurement, the triplet lifetime was determined for the specific conditions to reliably determine k_q. For the simulations shown in the main part, a triplet lifetime on the lower end of what was determined was used. By this, a lower limit of the “less is more” effect is shown, as a higher lifetime of the triplet state results in better quenching even at very low quencher concentrations.

However, the overall effects presented in this study are hardly influenced by a change in lifetime in a span of 40–60 μs, especially for synthetically relevant concentrations (>1 mM).

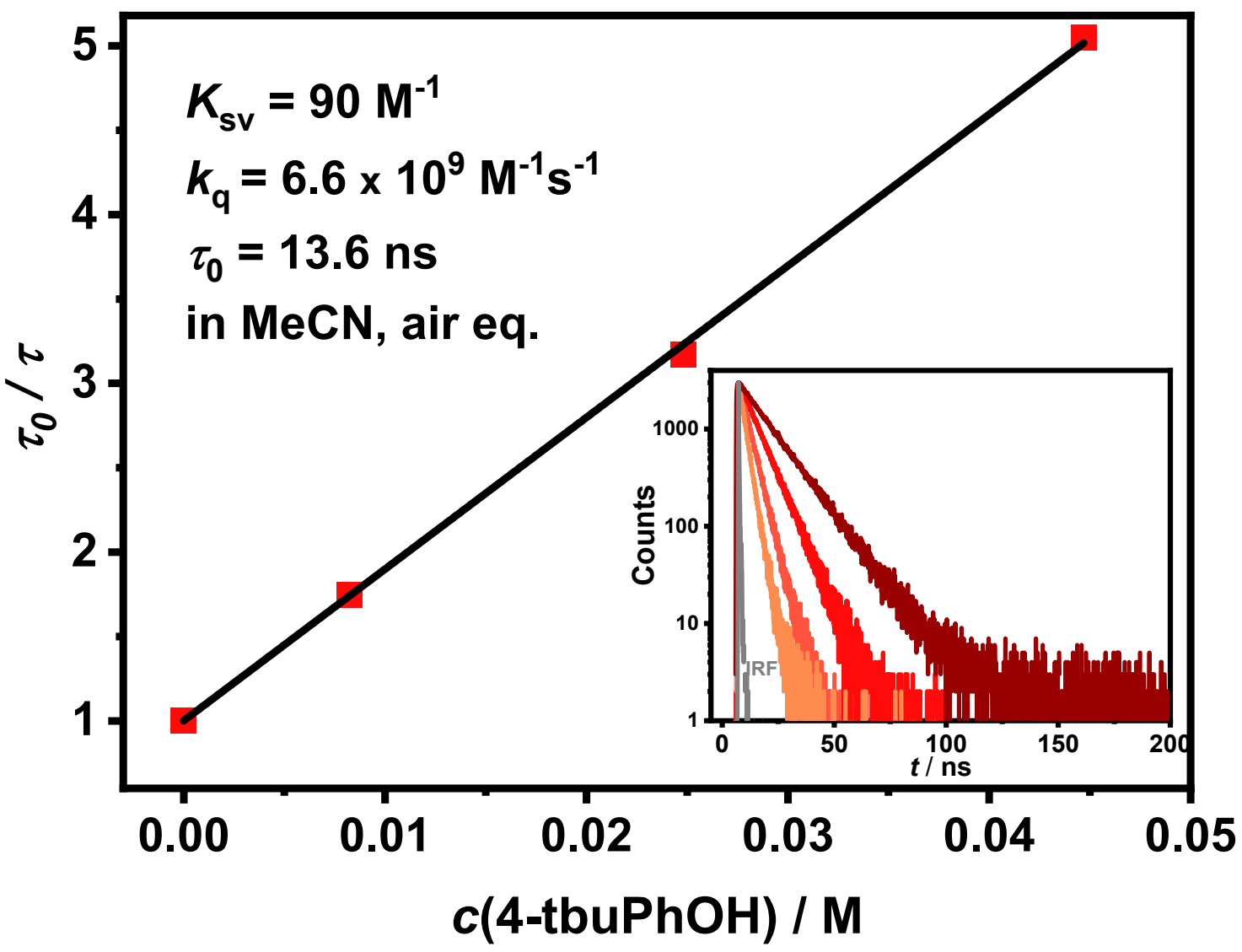

Fig. S8. Stern–Volmer plot for S_1 quenching of **PC^{2+}** (~5 μM) by **4-tbuPhOH** in air-equilibrated acetonitrile using TCSPC. Raw decay data sets, along with the instrument response function, are shown in the inset.

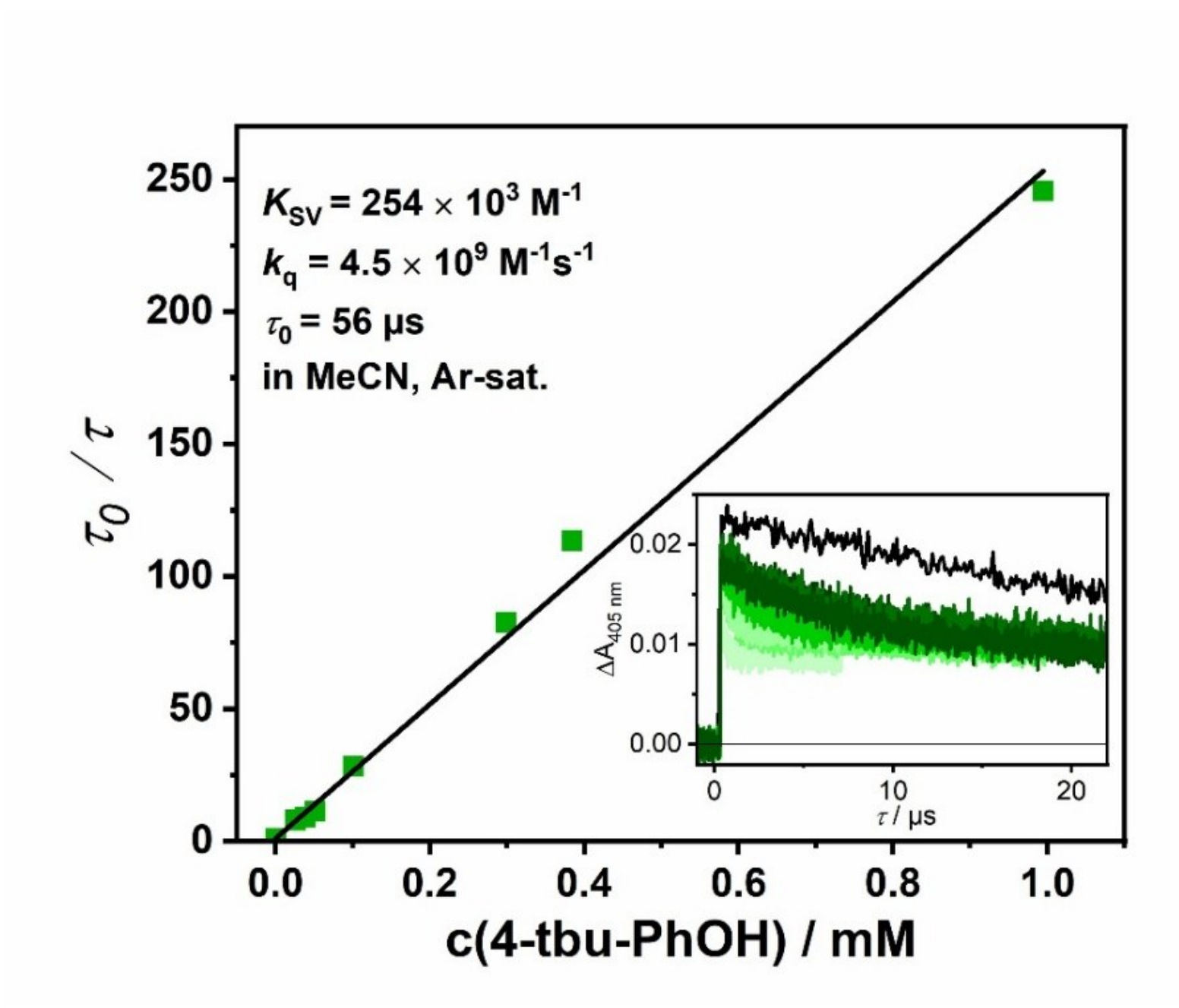

Fig. S9. Stern–Volmer plot for the T_1 quenching of **PC^{2+}** (~5 μM) by **4-tbuPhOH** in argon-saturated acetonitrile. Time-resolved transient absorption decays (λ_{exc} = 355 nm, 8 mJ/pulse) were monitored at 405 nm, and the extracted lifetimes were used to construct the Stern–Volmer plot. The incomplete decay to baseline (raw decay data in inset) upon quencher addition is due to concomitant absorption of **PC$^{\bullet+}$** formed upon quenching. Therefore, only the short-lived component of the biexponential decay was used for the analysis.

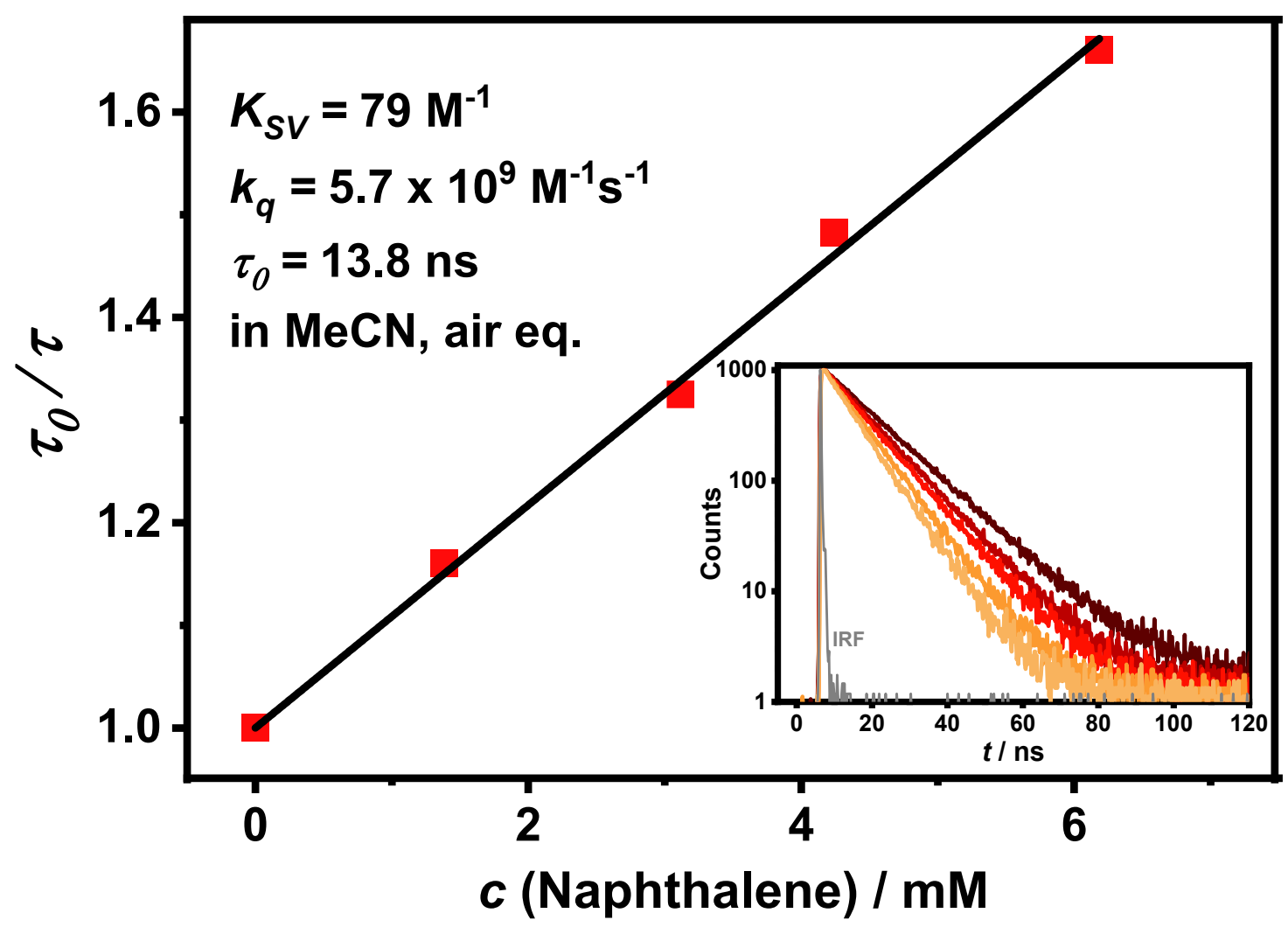

Fig. S10. Stern–Volmer plot for S_1 quenching of **PC^{2+}** (~5 μM) by **naphthalene** in air-equilibrated acetonitrile using TCSPC. Raw decay data sets, along with the instrument response function, are shown in the inset.

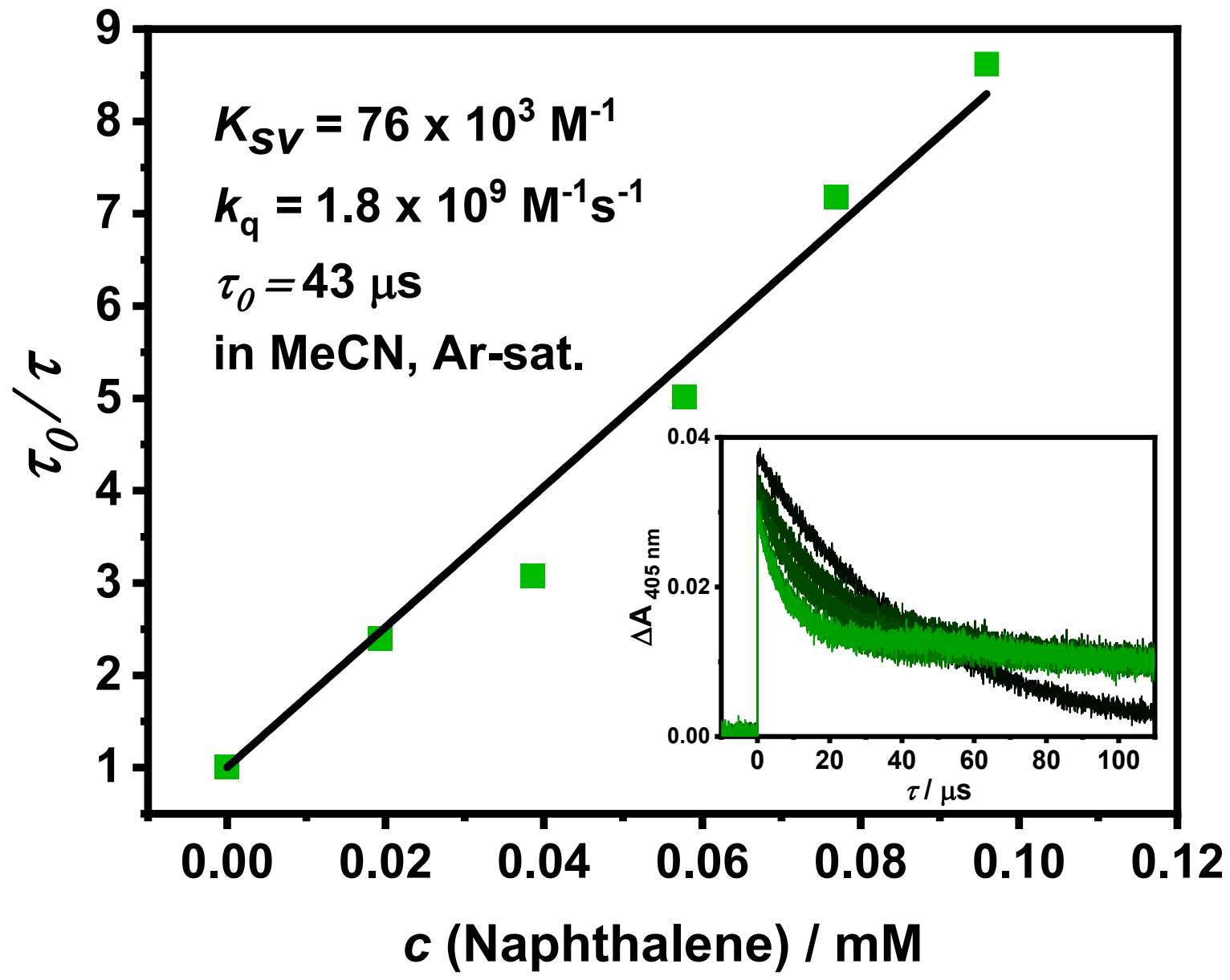

Fig. S11. Stern–Volmer plot for the T_1-state quenching of **PC^{2+}** (~5 μM) by **naphthalene** in argon-saturated acetonitrile. Time-resolved transient absorption decays (λ_{exc} = 355 nm, 5 mJ/pulse) were monitored at 405 nm, and the extracted lifetimes were used to construct the Stern–Volmer plot. The incomplete decay to baseline (raw decay data in inset) upon quencher addition is due to concomitant absorption of **PC$^{•+}$** formed upon quenching. Therefore, only the short-lived component of the biexponential decay was used for the analysis.

S2.4 Determination of Quantum Yield of Free Radical Formation $\Phi_{r,tot}$

The quantum yield of free radical formation $\Phi_{r,tot}$ is defined as the ratio between the concentration of free radicals of the reduced photocatalyst (**PC$^{•+}$**), which equals the concentration of the oxidized quencher, and the concentration of excited states generated per laser pulse (***PC^{2+}**). As described in Equations 1 and 2 in the main text, this yield includes the contributions of radicals formed upon quenching of both T_1 and S_1:

$$\Phi_{r,tot} = \Phi_{r,s} + \Phi_{r,T} = \frac{[PC^{•+}]}{[{}^*PC^{2+}]}$$

The concentration of **PC$^{•+}$** was calculated using the Beer–Lambert law from the transient absorption signal recorded at 450 nm, using the differential extinction coefficient determined in section S2.1, for 1 cm-pathlength cuvettes:

$$\Delta A_{450\ nm} = \Delta\varepsilon_{450\ nm} \times [PC^{•+}] \times 1\ cm$$

To isolate the concentration of radicals that successfully escaped the solvent cage but prior to bulk back-electron transfer, values were averaged within a specific time window where the signal remains constant**.** The selected time window for each set of measurements is specified in the following sections.

The concentration of ***PC^{2+}** was determined by relative actinometry employing an aqueous solution of **[Ru(bpy)$_3$]Cl$_2$**, iso-absorbing with the **PC^{2+}**/quencher solution at the excitation wavelength (355 nm). The ground-state bleach of the actinometer was monitored at 455 nm and fitted exponentially to time zero ($\Delta A_{0,\ 455\ nm}$) in order to determine the concentration of excited states after the laser pulse, being the differential extinction coefficient literature-known ($\Delta\varepsilon_{455\ nm}$= -10100 $M^{-1}cm^{-1}$).[5,6]

$$\Delta A_{455\ nm}(t) = \Delta A_{0,455\ nm} \times e^{-t/\tau}$$

Quencher Concentration – Dependent $\Phi_{r,tot}$: 4-*tert*-butylphenol

$\Phi_{r,tot}$ was determined for ~5 µM **PC^{2+}** solutions at increasing phenol concentrations. Each set of measurements was performed using individually prepared sample and actinometric solutions, as well as slightly different laser pulse intensities. Therefore, the absolute ΔA values in the raw data shown below are not directly comparable between measurements.

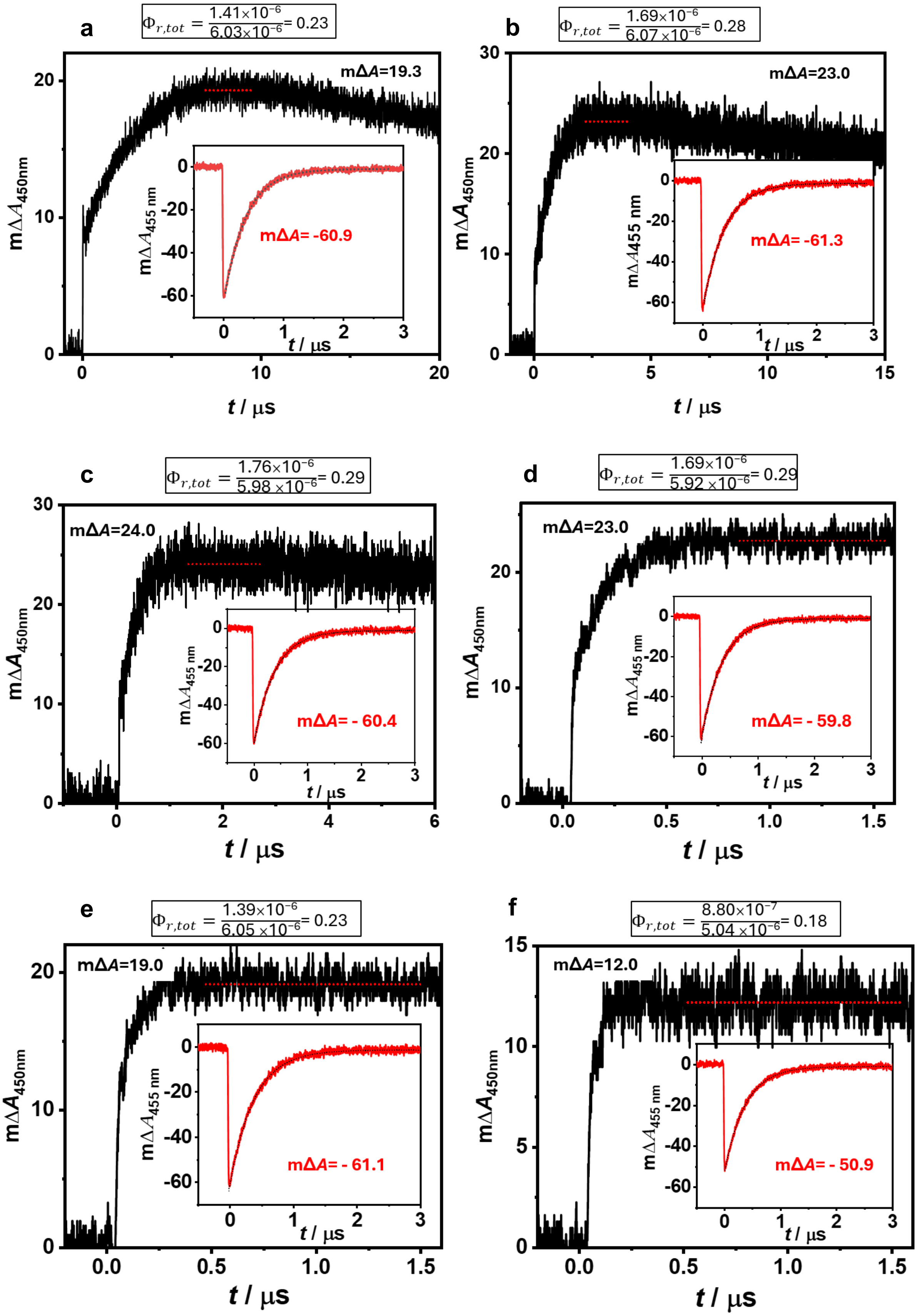
a
$\Phi_{r,tot} = \frac{1.41\times10^{-6}}{6.03\times10^{-6}} = 0.23$
mΔA=19.3
mΔA= -60.9
$m\Delta A_{450nm}$
$m\Delta A_{455\ nm}$
t / μs
b
$\Phi_{r,tot} = \frac{1.69\times10^{-6}}{6.07\times10^{-6}} = 0.28$
mΔA=23.0
mΔA= -61.3
$m\Delta A_{450nm}$
mΔA455 nm
t / μs
c
$\Phi_{r,tot} = \frac{1.76\times10^{-6}}{5.98\times10^{-6}} = 0.29$
mΔA=24.0
mΔA= - 60.4
$m\Delta A_{450nm}$
$m\Delta A_{455\ nm}$
t / μs
d
$\Phi_{r,tot} = \frac{1.69\times10^{-6}}{5.92\times10^{-6}} = 0.29$
mΔA=23.0
mΔA= - 59.8
$m\Delta A_{450nm}$
$m\Delta A_{455\ nm}$
t / μs
e
$\Phi_{r,tot} = \frac{1.39\times10^{-6}}{6.05\times10^{-6}} = 0.23$
mΔA=19.0
mΔA= - 61.1
$m\Delta A_{450nm}$
$m\Delta A_{455\ nm}$
t / μs
f
$\Phi_{r,tot} = \frac{8.80\times10^{-7}}{5.04\times10^{-6}} = 0.18$
mΔA=12.0
mΔA= - 50.9
$m\Delta A_{450nm}$
$m\Delta A_{455\ nm}$
t / μs

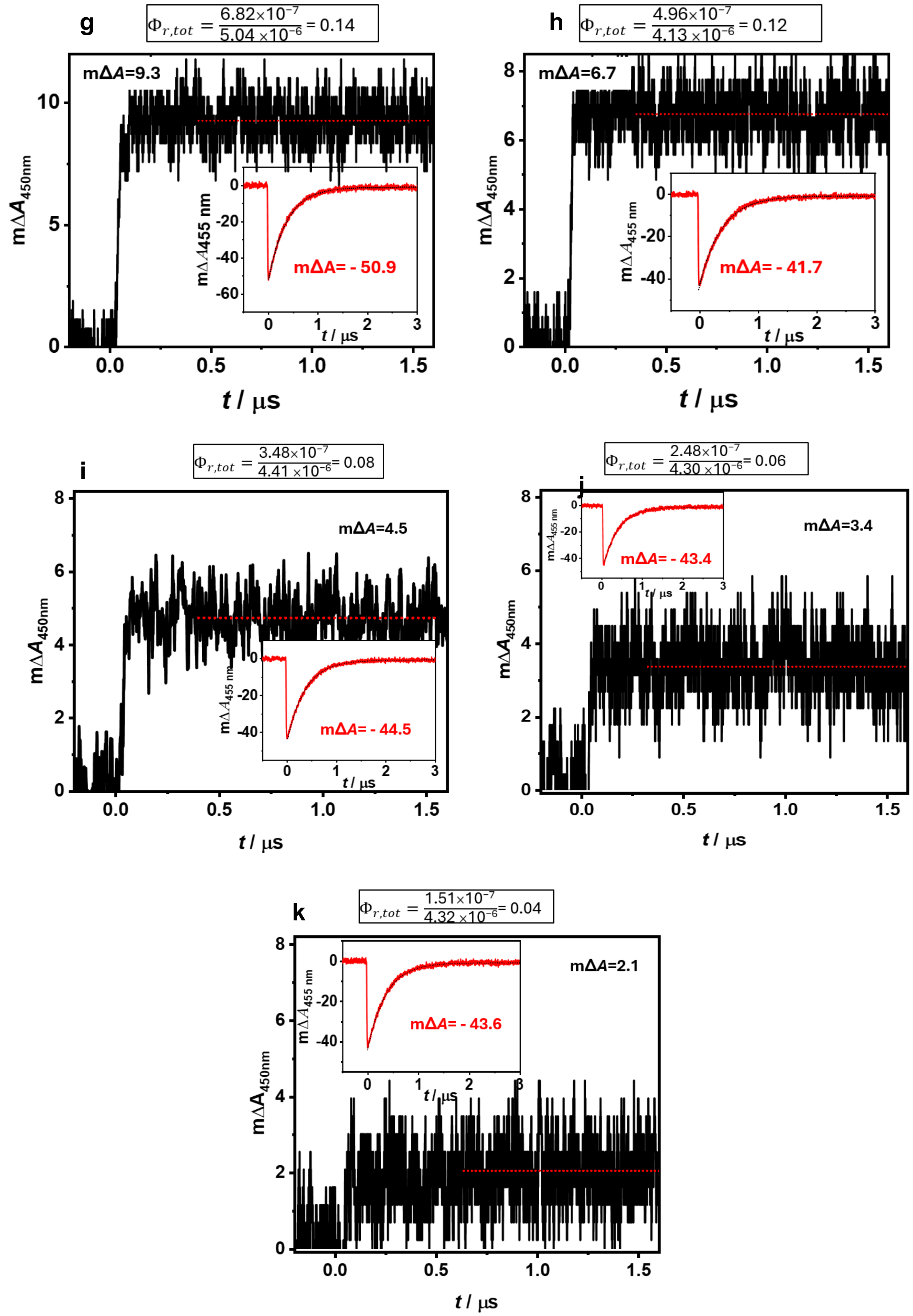

Fig. S12. Time-resolved absorption traces recorded at 450 nm (λ_{exc}= 355 nm, 5-7 mJ/pulse) for solutions ~5 μM of **PC**$^{2+}$ at different quencher concentrations (**a**→**k**: 0.075→174.8 mM of the phenol). Red dotted lines indicate the time window used for signal averaging. Inset: corresponding time-resolved absorption trace recorded at 455 nm for the solutions used for relative actinometry.

Quencher Concentration – Dependent $\Phi_{r,tot}$: naphthalene

$\Phi_{r,tot}$ was determined for ~5 µM **PC^{2+}** solutions at increasing naphthalene concentrations. Each set of measurements was performed using individually prepared sample and actinometric solutions, as well as slightly different laser pulse intensities. Therefore, the absolute ΔA values in the raw data shown below are not directly comparable between measurements unless otherwise stated.

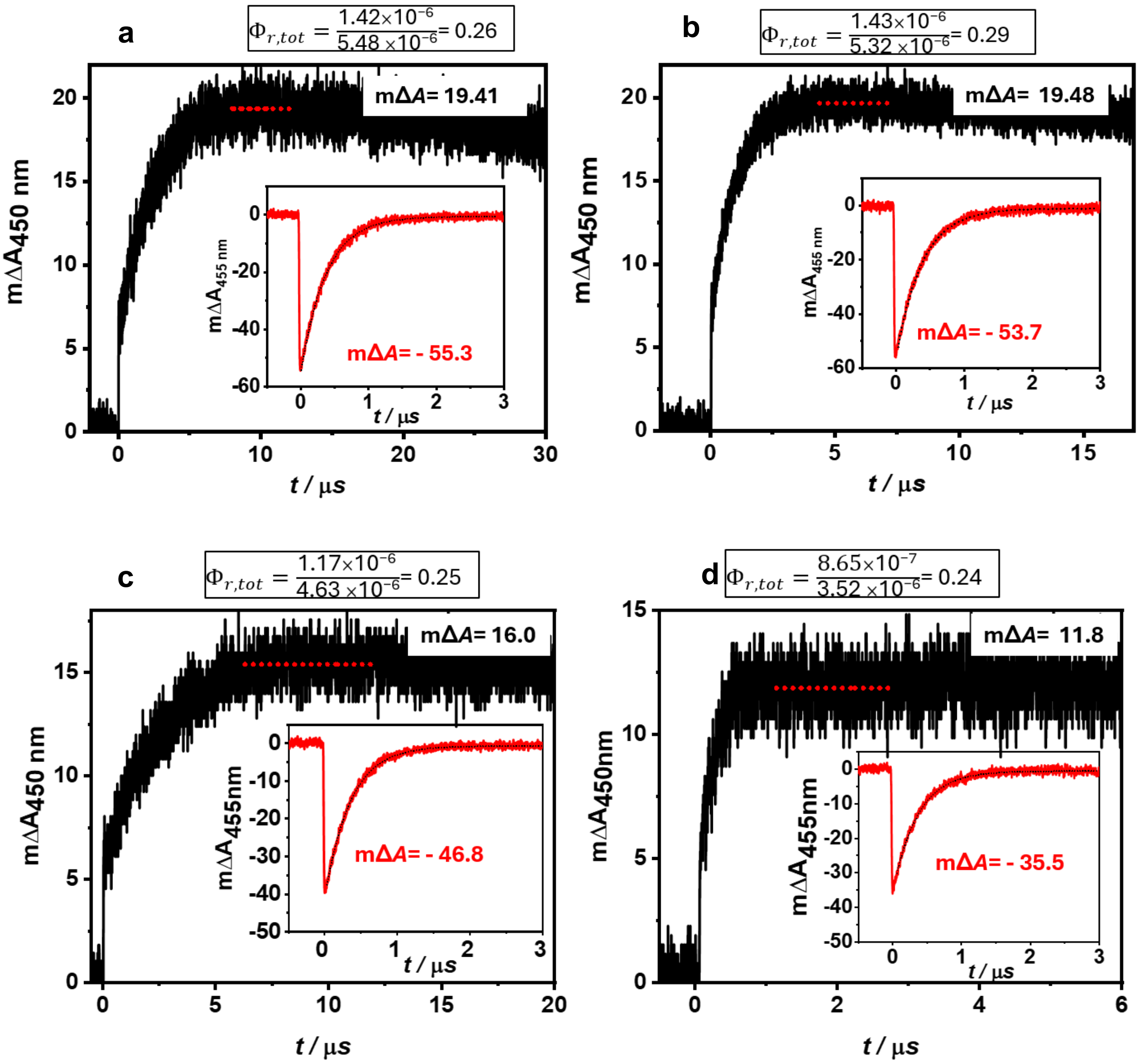

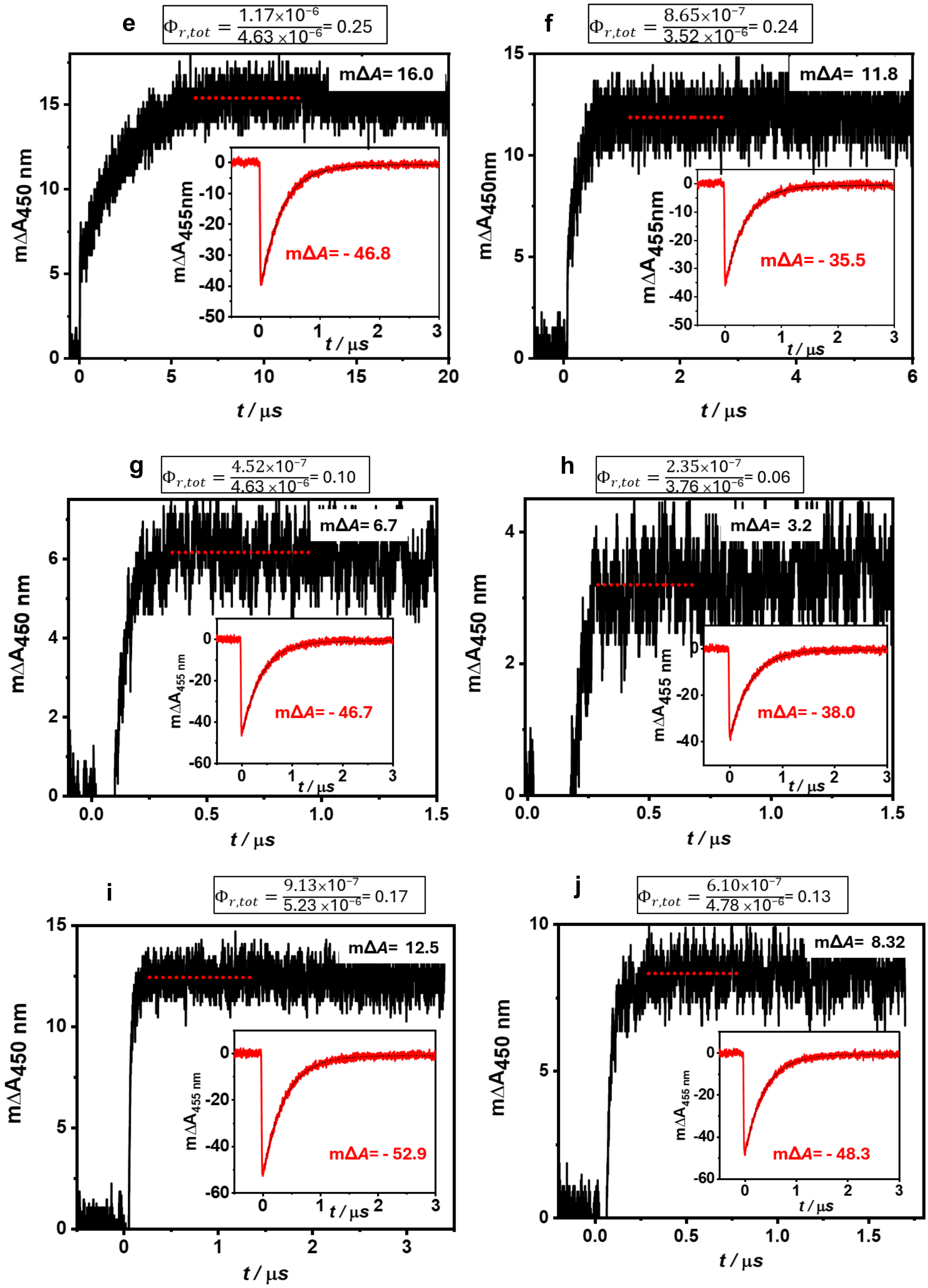

Fig. S13. Time-resolved absorption traces recorded at 450 nm for solutions ~5μM of PC^{2+} at different quencher concentrations (**a**→**j**: 0.18→ 220 mM of naphthalene), upon 355 nm excitation (laser intensity 5-7 mJ/pulse). Red dotted lines indicate the time window used for signal averaging. Inset: corresponding time-resolved absorption trace recorded at 455 nm for the actinometric solutions.

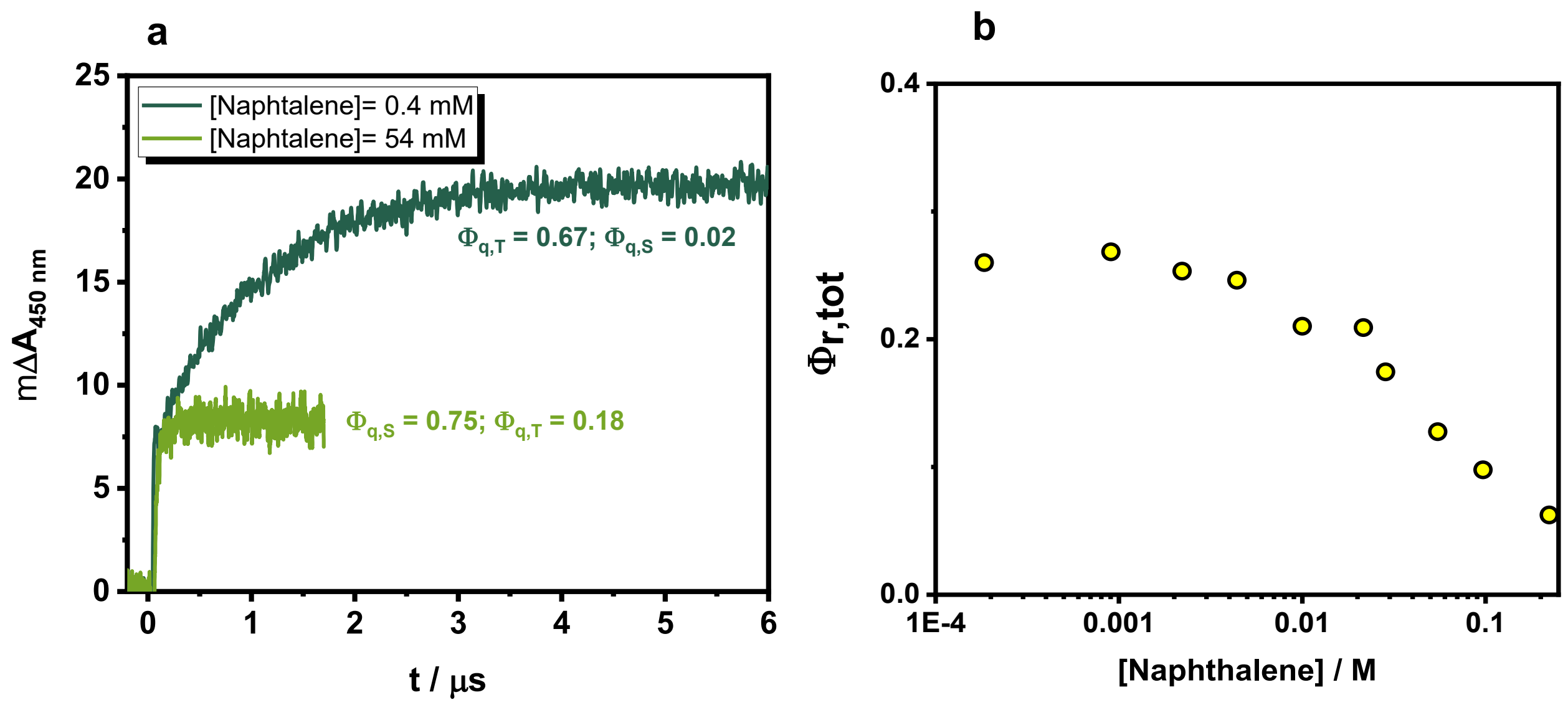

Fig. S14. (a) Time-resolved absorption traces recorded at λ = 450 nm, corresponding to the absorption of **PC**$^{\bullet+}$ generated via photoinduced electron transfer from acetonitrile solutions of **PC**$^{2+}$ at different naphthalene concentrations, under identical experimental conditions (355 nm laser excitation, 5.5 mJ/pulse, and matched ground-state absorption at the excitation wavelength); **(b)** quantum yields for free radical production as function of quencher concentration, determined from raw data sets shown in Fig. S13 a-j.

S2.5 Determination of Cage Escape Efficiency, η_{ce}

General Procedure: Cage Escape Efficiency upon Quenching of T_1, $\eta_{ce,T}$

The cage escape quantum yield of free radical formation ($\Phi_{r,tot}$) is first determined, according to the method described in section S2.4. Appropriately low quencher concentrations [Q] are employed to ensure that radical production arises almost exclusively from T_1 quenching ($\eta_{q,S} \leq 0.02$), so that $\Phi_{r,T}$ = $\Phi_{r,tot}$, and therefore:

$$\Phi_{r,T} = \eta_{isc} \times \eta_{q,T} \times \boldsymbol{\eta_{ce,T}} = \frac{k_{isc}}{\frac{1}{\tau_{0,S}} + k_{q,s}\,[Q]} \times \frac{k_{q,T}\,[Q]}{\frac{1}{\tau_{0,T}} + k_{q,T}\,[Q]} \times \boldsymbol{\eta_{ce,T}}$$

$\tau_{0,S}$ is the lifetime of the unquenched S_1 state, determined in a previous study by measuring prompt fluorescence decay via TCSPC in an Ar-saturated acetonitrile solution in the absence of quencher.[4] $\tau_{0,T}$ is the lifetime of the unquenched T_1 state, determined by monitoring the decay of the transient absorption signal at 405 nm using transient absorption spectroscopy in an Ar-saturated solution upon 355 nm excitation (see for example in Fig. S11). k_{isc} is the intersystem crossing rate constant, determined according to the equation $k_{isc} = \Phi_{isc}/\tau_{0,S}$, where Φ_{isc} = 0.68 was determined for the same photocatalyst in a previous study.[4] $k_{q,S}$ and $k_{q,T}$ are the quenching rate constants for S_1 and T_1 state respectively, determined in section S2.3, for the quencher used in the experiment.

Given the known decay constants of the system, the quencher concentration, and the experimentally determined $\Phi_{r,T}$, the unknown, $\eta_{ce,T}$, is determined. The procedure is repeated on the same quencher/actinometer solution at increasing laser intensities to

confirm the linear response of the generated concentrations with excitation energy and to obtain an averaged value of $\eta_{ce,T}$ from at least three independent measurements.

General procedure: Cage Escape Efficiency upon Quenching of S_1, $\eta_{ce,S}$

Under the experimental conditions employed to probe $\eta_{ce,S}$, quantitative S_1 quenching cannot be achieved due to the presence of a residual, non-negligible triplet population ($\eta_{isc} \neq 0$). As the triplet manifold is intrinsically more productive in terms of free radical generation, its contribution must be accounted for in the analysis.

To isolate the S_1 contribution, the T_1 derived component of the overall free radical production quantum yield is subtracted from the total measured value, as described in section S2.4. The resulting S_1-derived quantum yield of free radical formation ($\Phi_{r,s}$) is then used to determine the cage escape efficiency of the singlet pathway ($\eta_{ce,S}$) according to the equation:

$$\Phi_{r,S} = \eta_{q,S} \times \boldsymbol{\eta_{ce,S}} = \frac{k_{q,s}[Q]}{\frac{1}{\tau_{0,S}} + k_{q,s}[Q]} \times \boldsymbol{\eta_{ce,S}}$$

The same procedure described for the triplet-state analysis is applied to the singlet-state quenching. As described in the previous section, using the known kinetic constants, quencher concentration, and experimentally determined $\Phi_{r,s}$, the unknown parameter $\eta_{ce,S}$ can be obtained. Measurements are repeated at increasing laser intensities under identical conditions to confirm linearity with excitation energy and to derive an average value from at least three independent experiments.

S2.6 Experiment Series 1: Quencher 4-*tert*-butylphenol

Serie 1a: Determination of $\eta_{ce,T}$

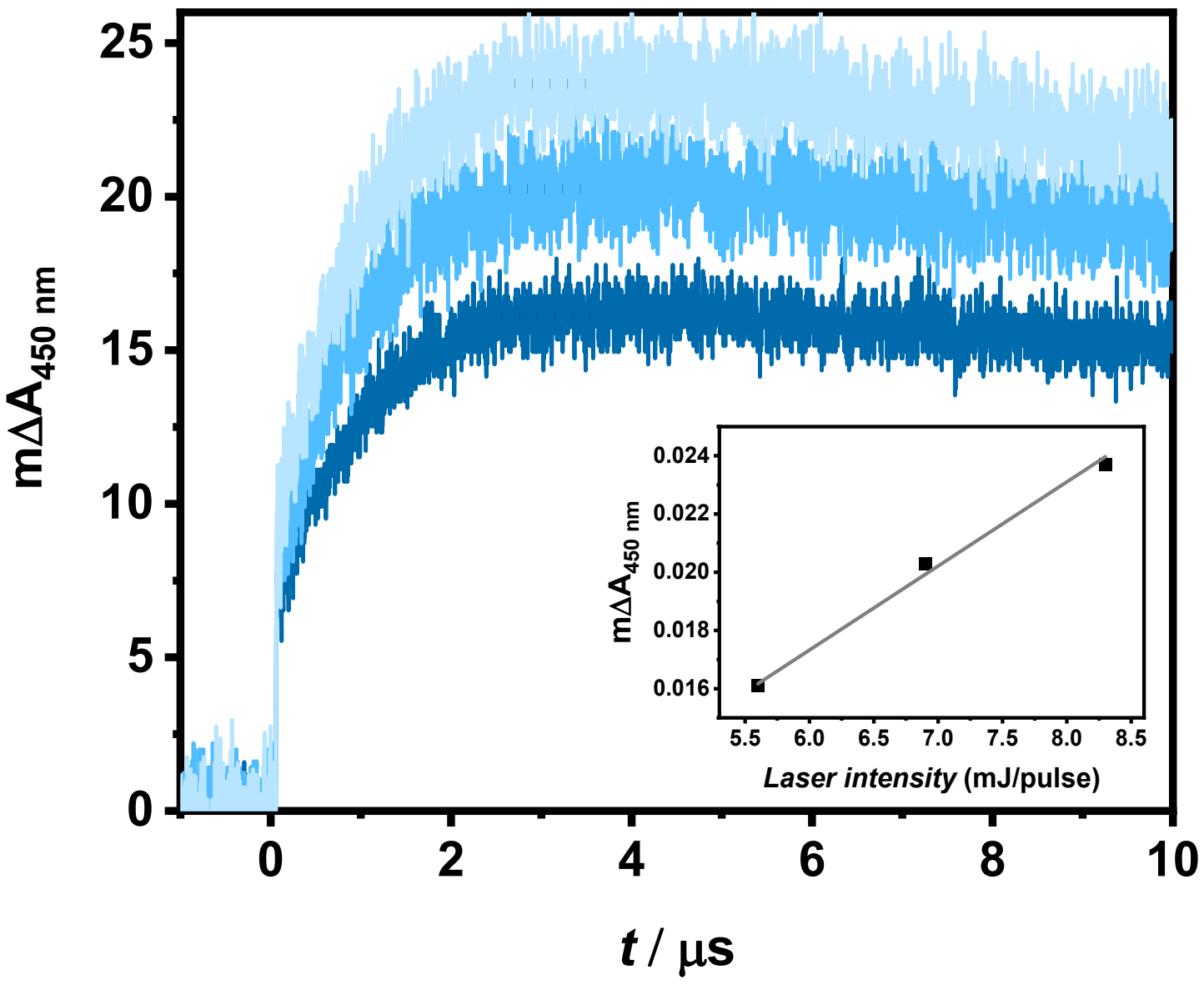

Fig. S15. Time-resolved transient absorption traces after laser excitation of an Ar-saturated acetonitrile solution containing ~5 µM of **PC**$^{2+}$ with **0.22** mM of **4-tbuPhOH** after laser excitation (λ_{exc} = 355 nm) with increasing laser intensities. The dotted lines indicate the time interval for data averaging to determine ΔA values. The inset shows the linear dependence of signal intensity on laser energy, with a line showing the trend.

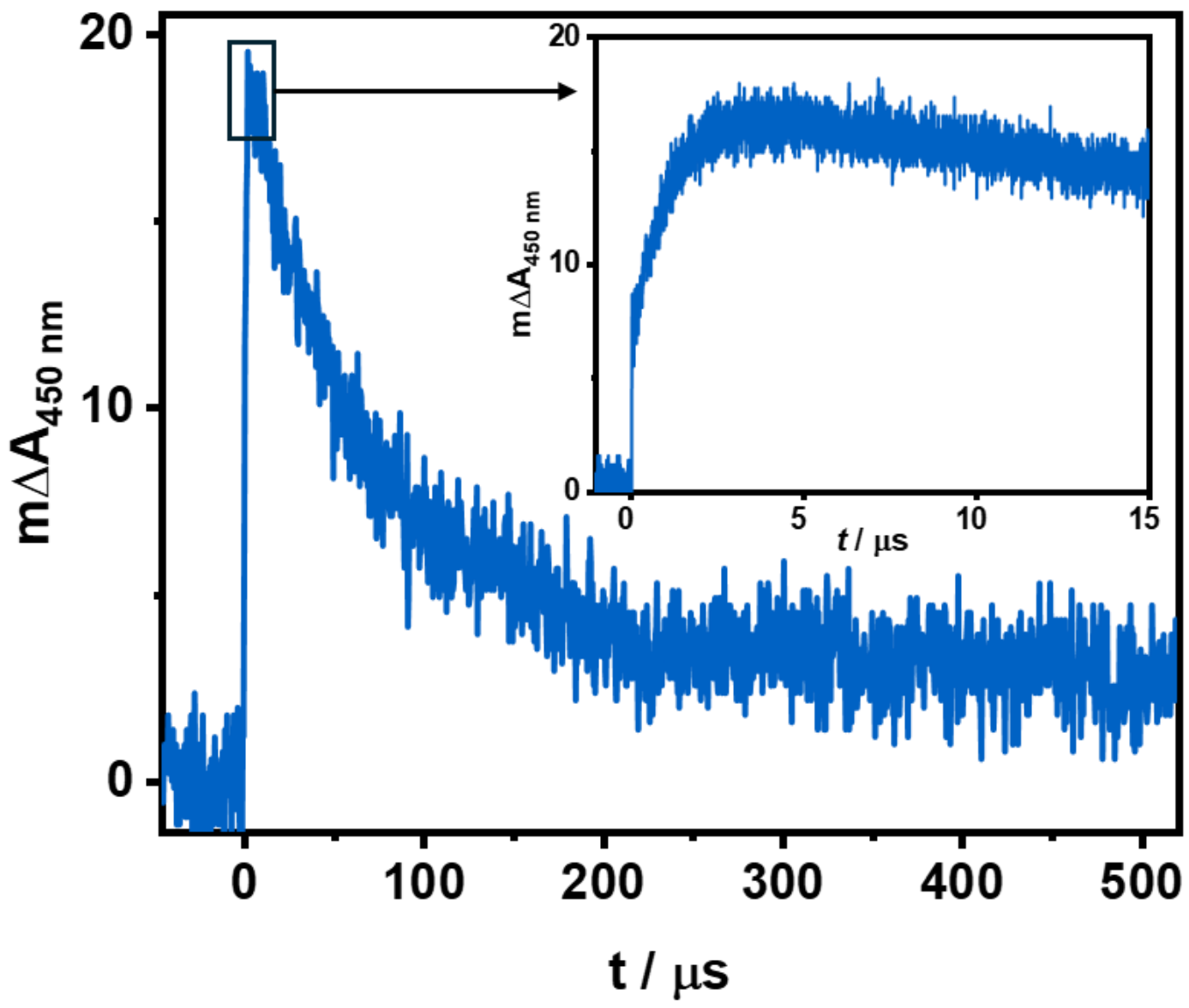

Fig. S16. Time-resolved transient absorption trace upon 355 nm laser excitation (5.5 mJ/pulse) of Ar-saturated acetonitrile solution ~5 µM of **PC**$^{2+}$ with 0.22 mM of **4-tbuPhOH**. The signal recorded on a 500 µs time scale shows the decay of the radical **PC**$^{•+}$ in solution and is compared to the time window for ΔA integration (inset) used to quantify **PC**$^{•+}$ immediately after the cage escape.

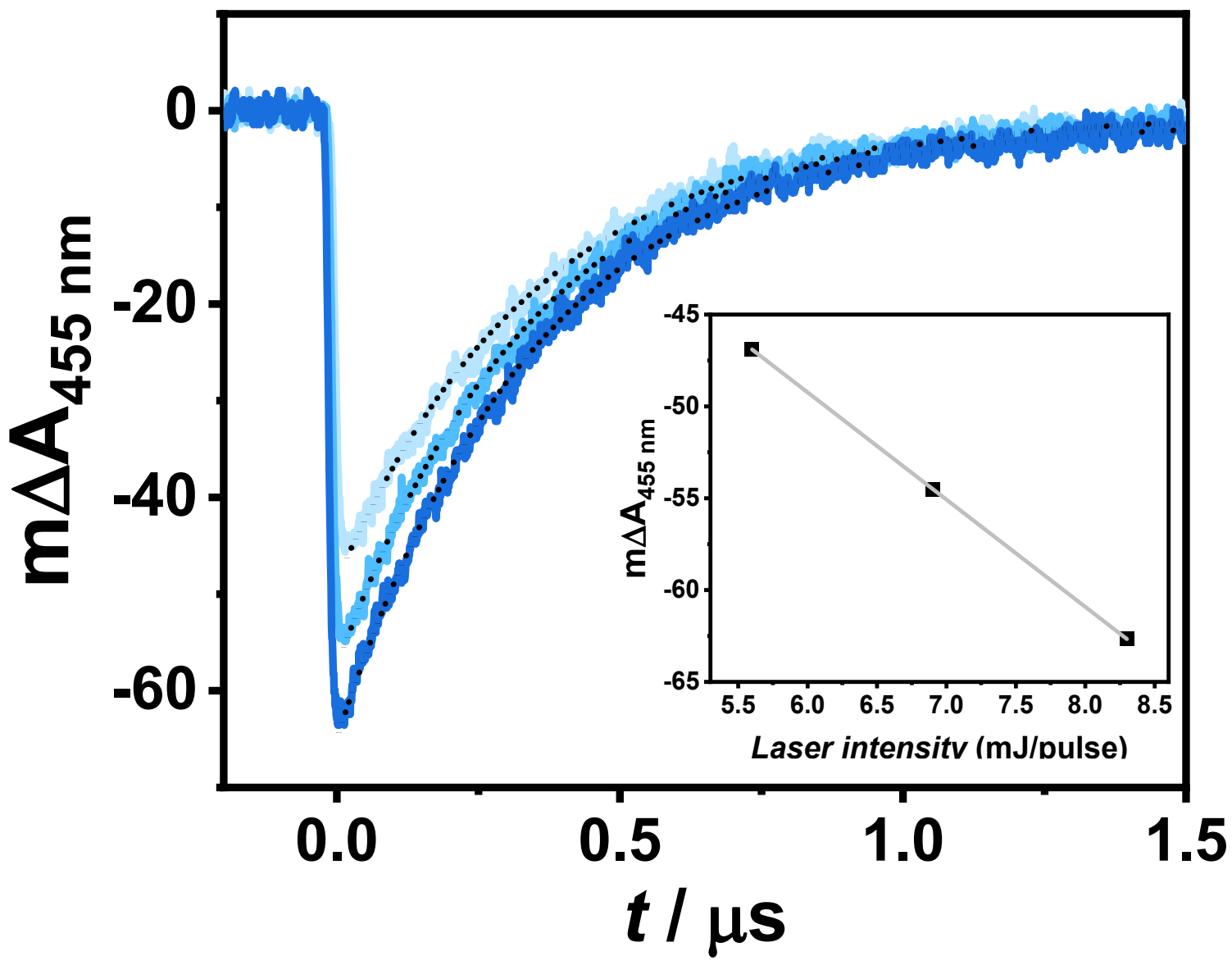

Fig. S17. Time-resolved transient absorption traces following laser excitation of air-saturated aqueous solutions of **$[Ru(bpy)_3]Cl_2$**. The concentration was adjusted to match the ground state absorption of the **PC^{2+}**/quencher solution at 355 nm. The signal at 455 nm corresponds to the ground-state bleach and exponential fitting curves (dotted lines) of the decay traces were used to extrapolate the ΔA_0 values. The inset shows the linear dependence of signal intensity on laser pulse energy, with a line showing the trend.

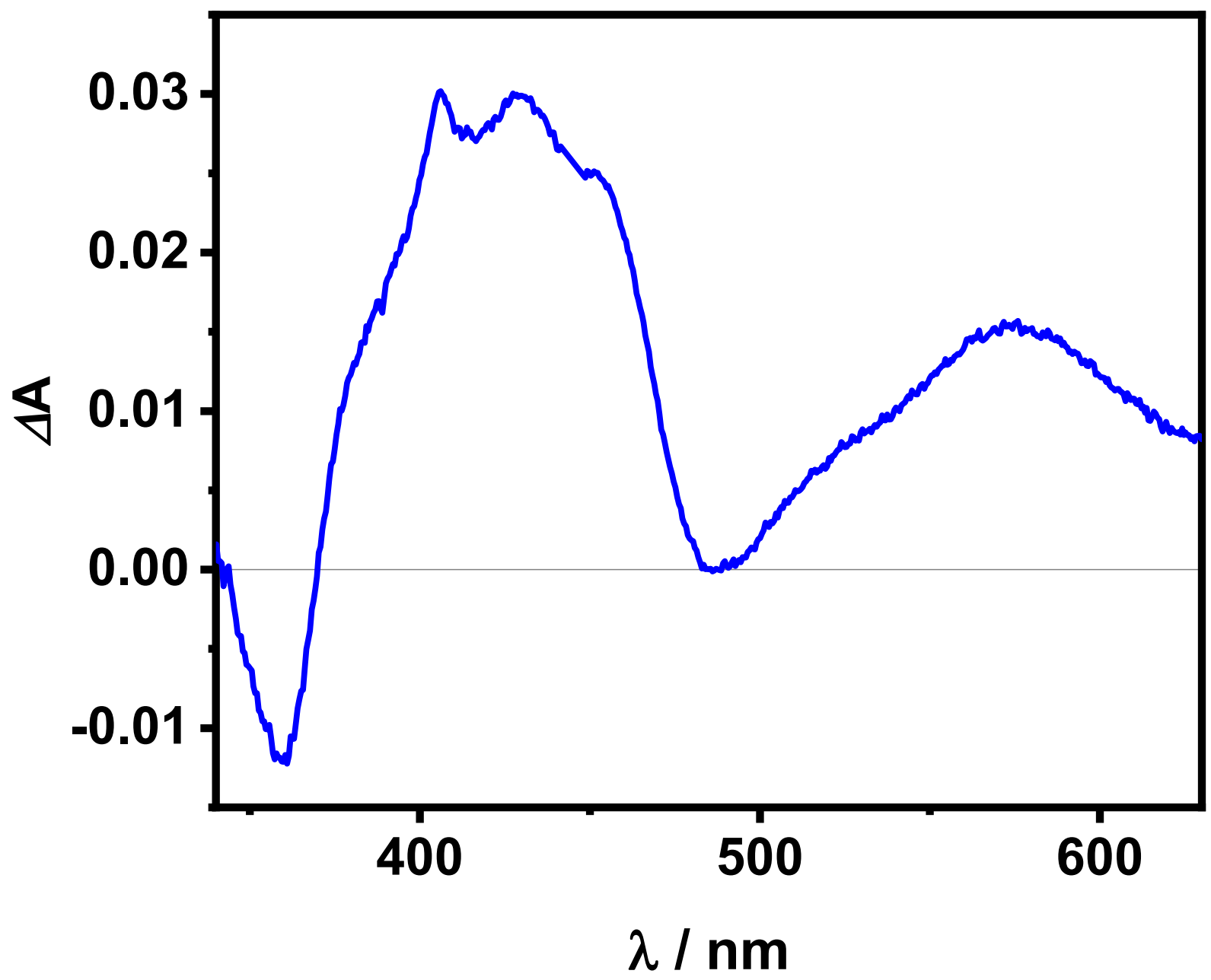

Fig. S18. Transient absorption spectrum of an Ar-saturated acetonitrile solution of **PC^{2+}** with 0.22 mM of **4-tbuPhOH** recorded with 5 µs delay after 355 nm excitation using a laser pulse energy of 12 mJ per pulse. The peak at 405 nm matches the literature-reported absorption of the phenoxyl radical,[7] while the other spectral features are consistent with those shown in Fig. S4, which were assigned to the reduced photocatalyst **$PC^{\bullet+}$**.

Table S2. Experimental data and key photophysical parameters required for the determination of cage escape efficiency following quenching of the triplet excited state of **PC²⁺** by **4-tbuPhOH**.

Laser intensity (mJ/pulse)	mΔA_0 at 455 nm	[PC*]/M	mΔA at 450 nm	[PC$^{\bullet+}$]/M	[PC$^{\bullet+}$]/[PC*]	$\Phi_{q,T}$	$\eta_{ce,T}$	$\eta_{ce,T}$ average
5.6	-46.9	4.6 ×10^{-6}	16.1	1.18 ×10^{-6}	0.25		0.39	
6.9	-54.5	5.4 ×10^{-6}	20.3	1.49 ×10^{-6}	0.27	0.65	0.42	**0.42 ± 0.02**
8.3	-62.6	6.2 ×10^{-6}	23.7	1.74 ×10^{-6}	0.28		0.43	

Series 1b: Determination of $\eta_{ce,S}$

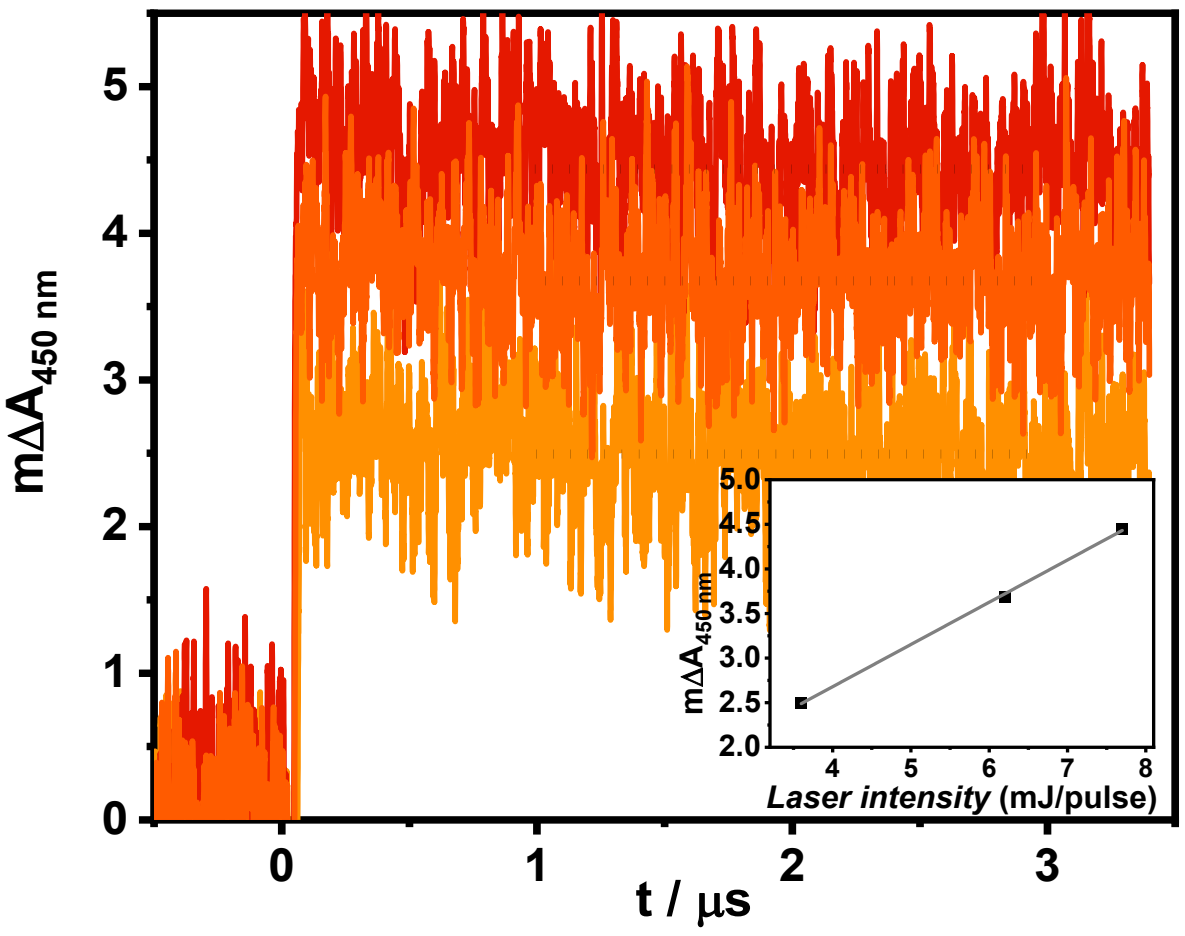

Fig. S19. Time-resolved transient absorption traces after laser excitation of Ar-saturated acetonitrile solutions ~5 µM of **PC²⁺** and 132 mM of **4-tbuPhOH** after laser excitation (λ_{exc} = 355 nm) at increasing laser pulse intensities. The dotted lines indicate the time interval for data averaging to determine ΔA value. The inset shows the linear dependence of signal intensity on laser intensity, with a line showing the trend.

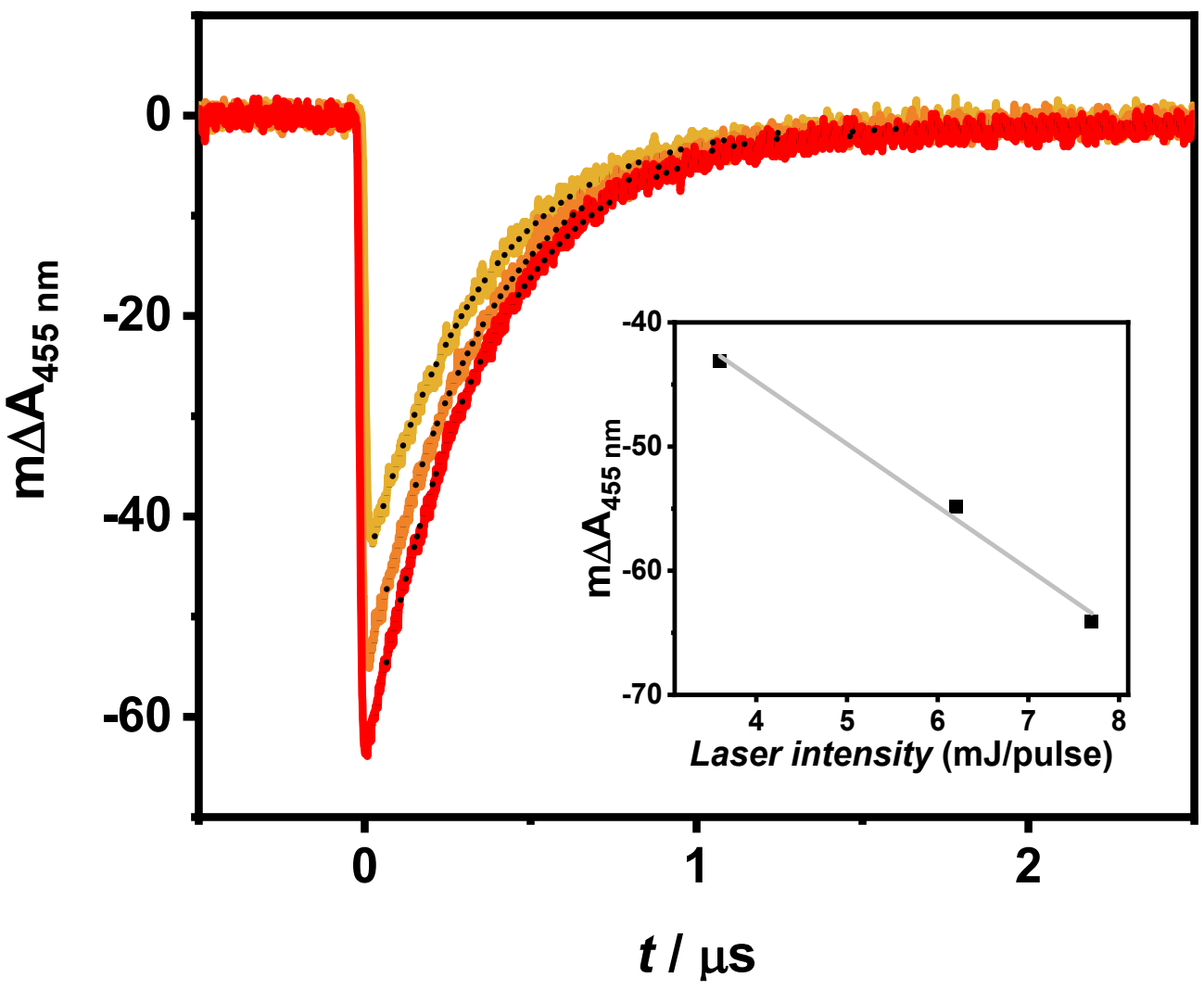

Fig. S20. Time-resolved transient absorption traces following laser excitation of air-saturated aqueous solutions of **[Ru(bpy)$_3$]Cl$_2$**. The concentration was adjusted to match the ground state absorption of the **PC²⁺**/quencher solution at 355 nm. The signal at 455 nm corresponds to the ground-state bleach and exponential fitting curves (dotted lines) of the decay traces were used to extrapolate the ΔA_0 values. The inset shows the linear dependence of signal intensity on laser pulse intensity, with a line indicating the trend.

Table S3. Experimental data and key photophysical parameters required for the determination of cage escape efficiency following quenching of the S_1 state of **PC^{2+}** by **4-tbuPhOH**.

**Laser intensity (mJ/pulse)**	**mΔA_0 at 455 nm**	**[PC*]/M**	**mΔA at 450 nm**	**[PC$^{•+}$]/M**	$\Phi_{q,T}$	$\Phi_{q,S}$	**[PC$^{•+}$]/[PC*] total**	**[PC$^{•+}$]/[PC*] From S_1**	$\eta_{ce,S}$	$\eta_{ce,T}$ **average**
3.6	-43.1	4.3×10^{-6}	2.5	1.8×10^{-7}	0.05	0.94	0.043	0.021	0.022	**0.028± 0.005**
6.2	-54.8	5.4×10^{-6}	3.7	2.7×10^{-7}			0.050	0.028	0.030	
7.7	-64.1	6.4×10^{-6}	4.5	3.3×10^{-7}			0.051	0.029	0.031	

The potential contribution of the phenol radical cation to the transient absorbance at 450 nm was evaluated by combining published spectral assignments with our kinetic data and probe-wavelength considerations. Reported spectra for the 4-tert-butylphenoxy radical cation (**4-tbuPhOH$^{•+}$**) show an absorption band tailing up to 460 nm, whereas the phenoxyl radical (**4-tbuPhO$^•$**) absorbs mainly up to 430 nm[7,8]; on the other hand, **PC$^{•+}$** exhibits a large molar absorptivity at 450 nm (≈13000 $M^{-1}cm^{-1}$), so that the **4-tbuPhOH$^{•+}$** signal at this wavelength would be comparatively small to remain undetected. Kinetically, the reported deprotonation time constant of **4-tbuPhOH$^{•+}$** in acetonitrile is ≈ 1.6 μs (which most likely depends on the actual water content in the solvent), and our singlet-quenching integration window (2–4 μs) could, in principle, include a residual fraction, producing a decaying component in that timescale followed by the persistence of the signal assigned to the longer-lived **PC$^{•+}$**. Crucially, the experimental traces at 450 nm do not display this two-component behavior, indicating that absorption of **4-tbuPhOH$^{•+}$** is negligible. Although measurements at 470 nm would further exclude any contribution of **4-tbuPhOH$^{•+}$**, the signal-to-noise ratio at 470 nm is lower in our conditions (due to concomitant lower absorptivity of **PC$^{•+}$**), making 450 nm a more reliable wavelength for quantification. Finally, any small positive bias in **PC$^{•+}$** yield arising from residual **4-tbuPhOH$^{•+}$** absorption would increase the apparent productivity of the singlet pathway; correcting for this effect would decrease the singlet cage escape efficiency and thus even strengthen the conclusion that the singlet channel is less productive than the triplet channel.

S2.7 Experiment Series 2: Quencher Naphthalene

Series 2a: Determination of $\eta_{ce,T}$

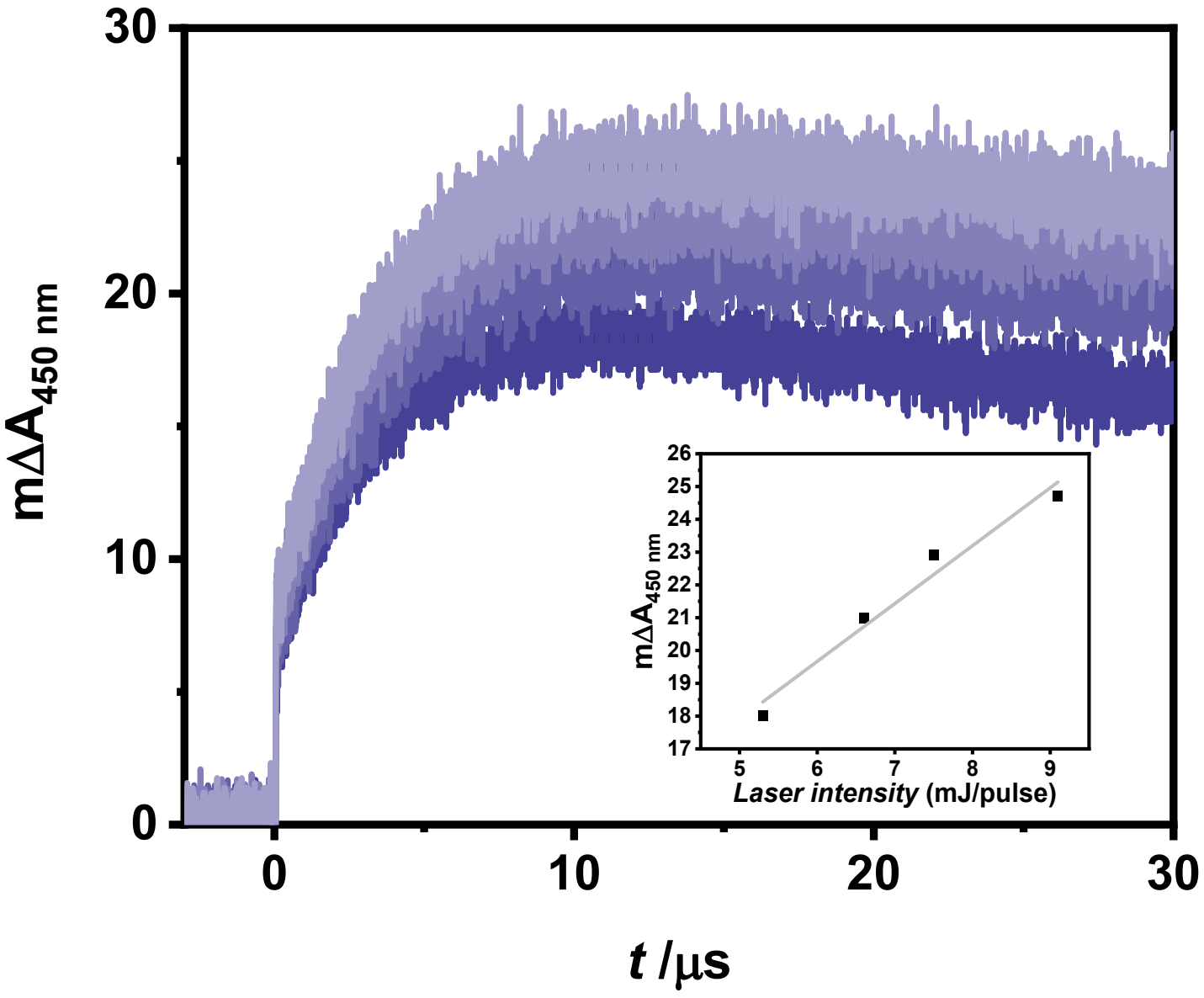

Fig. S21. Time-resolved transient absorption traces after laser excitation of Ar-saturated acetonitrile solutions containing ~5 µM of $\mathbf{PC^{2+}}$ and 0.12 mM of **naphthalene** after laser excitation (λ_{exc} = 355 nm) with increasing laser pulse intensities. The dotted lines indicate the time interval for data averaging to determine ΔA value. The inset shows the linear dependence of signal intensity on laser energy, with a line showing the trend.

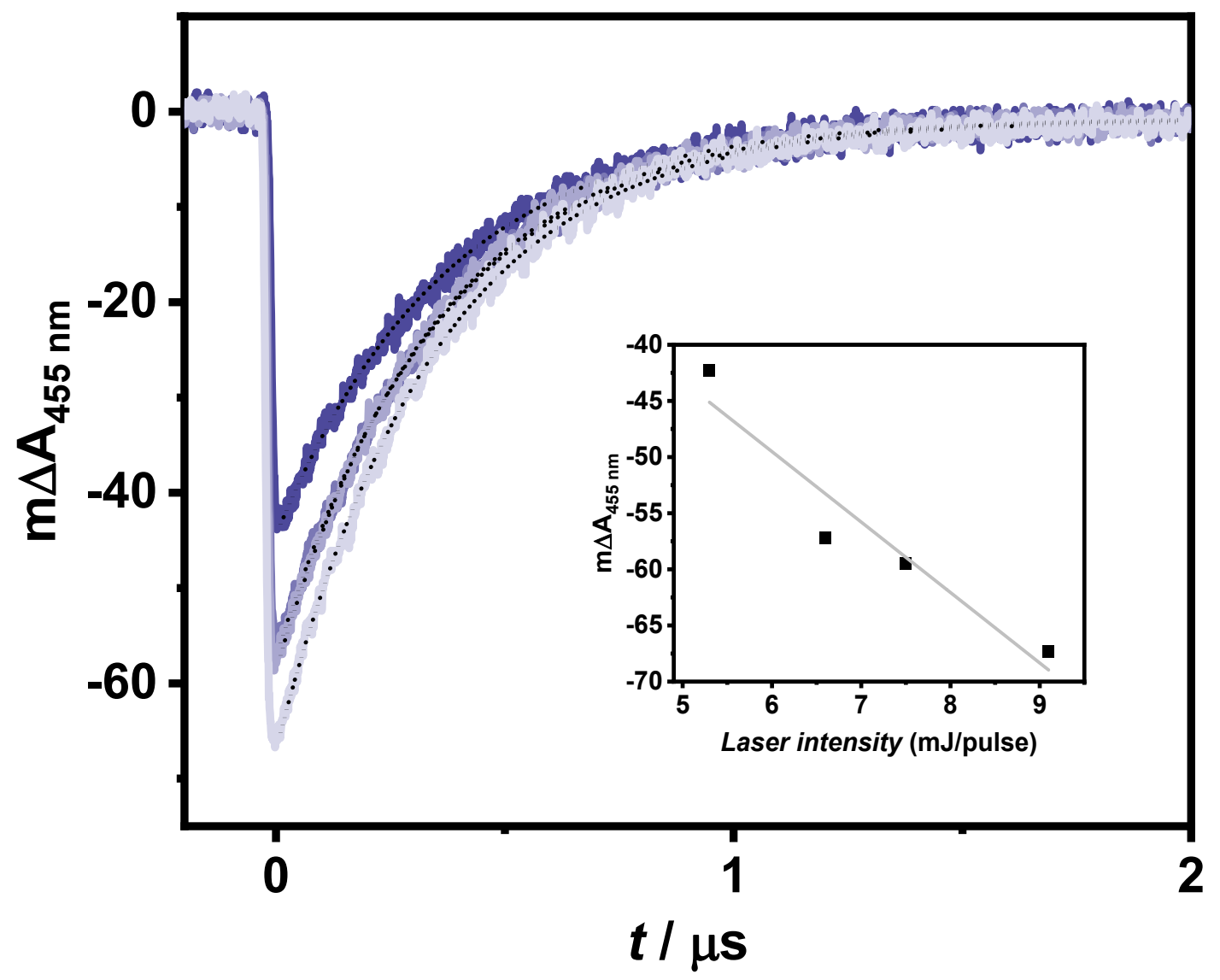

Fig. S22. Time-resolved transient absorption traces following laser excitation of air-saturated aqueous solutions of **[Ru(bpy)$_3$]Cl$_2$**. The concentration was adjusted to match the ground state absorption of the PC^{2+}/quencher solution at 355 nm. The signal at 455 nm corresponds to the ground-state bleach and exponential fitting curves (dotted lines) of the decay traces were used to extrapolate the ΔA_0 values. The inset shows the linear dependence of signal intensity on laser energy, with a line indicating the trend.

Table S4. Experimental data and key photophysical parameters required for the determination of cage escape efficiency following quenching of the T_1 state of **PC^{2+}** by **naphthalene**.

Laser intensity (mJ/pulse)	$m\Delta A_{t=0}$ at 455 nm	[PC*]/M	$m\Delta A_{average}$ at 450 nm	[$PC^{\cdot+}$]/M	[$PC^{\cdot+}$]/[PC*]	$\Phi_{q,T}$	$\eta_{ce,T}$	$\eta_{ce,T}$ average
5.3	-42.3	4.2×10^{-6}	18.0	1.3×10^{-6}	0.32	0.62	0.51	**0.46 ± 0.03**
6.6	-57.2	5.7×10^{-6}	21.0	1.5×10^{-6}	0.27		0.44	
7.5	-59.5	5.9×10^{-6}	22.9	1.7×10^{-6}	0.29		0.46	
9.1	-67.3	6.7×10^{-6}	24.7	1.8×10^{-6}	0.27		0.44	

Series 2b: Determination of $\eta_{ce,S}$

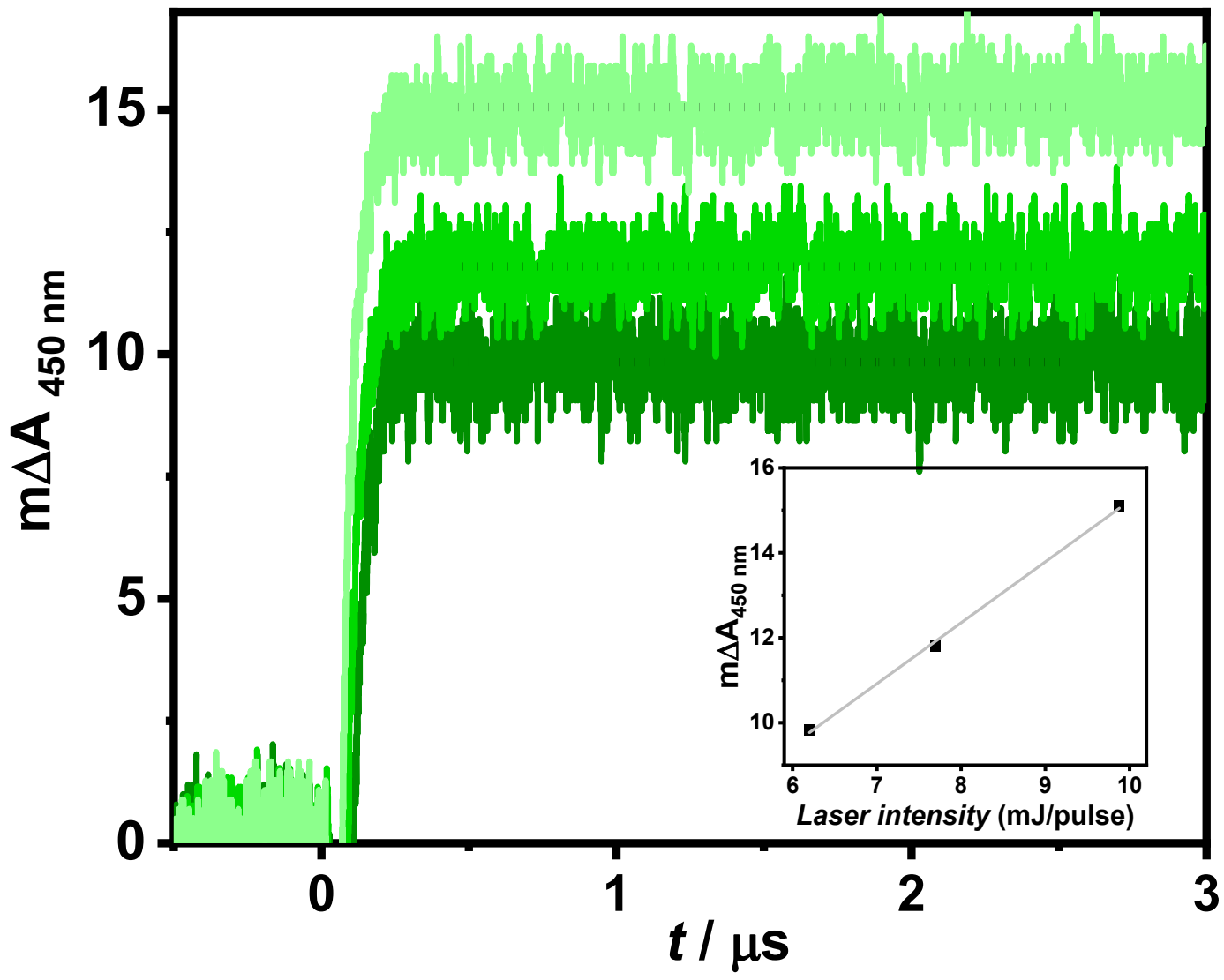

Fig. S23. Time-resolved transient absorption traces after laser excitation of Ar-saturated acetonitrile solutions containing ~5 µM of **PC^{2+}** and 132 mM of **naphthalene** after laser excitation (λ_{exc} = 355 nm) with increasing laser pulse intensities. The dotted lines indicate the time interval for data averaging to determine ΔA value. The inset shows the linear dependence of signal intensity on laser energy, with a line showing the trend.

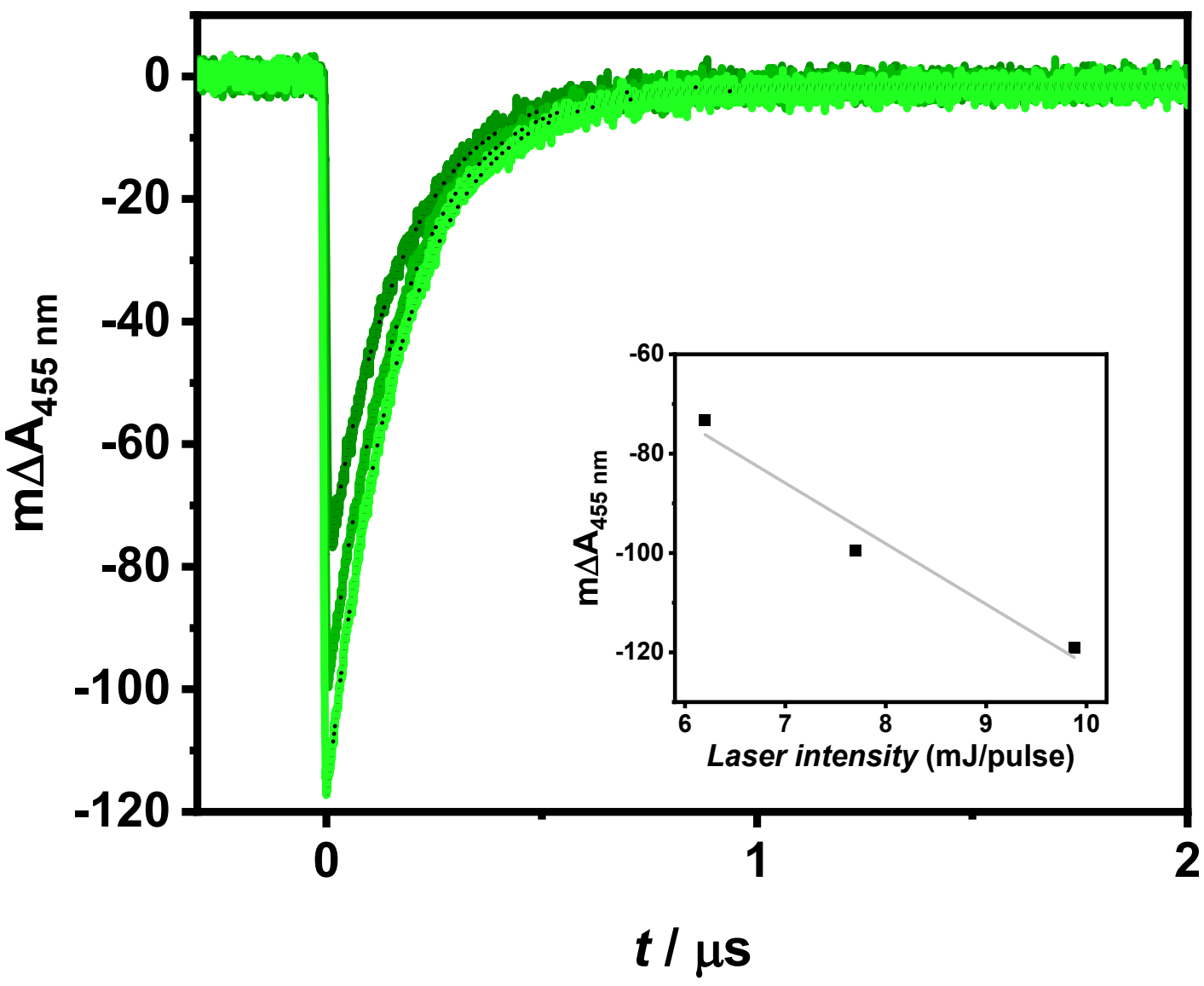

Fig. S24. Time-resolved transient absorption traces following laser excitation of air-saturated aqueous solutions of **$[Ru(bpy)_3]Cl_2$**. The concentration was adjusted to match the ground state absorption of the PC^{2+}/quencher solution at 355 nm. The signal at 455 nm corresponds to the ground-state bleach and exponential fitting curves (dotted lines) of the decay traces were used to extrapolate the ΔA_0 values. The inset shows the linear dependence of signal intensity on laser energy, with a line indicating the trend.

Table S5. Experimental data and key photophysical parameters required for the determination of cage escape efficiency following quenching of the S_1 state of **PC^{2+}** by **naphthalene**.

Laser intensity (mJ/pulse)	$\Delta A_{t=0}$ at 455 nm	$[PC^*]$/M	$\Delta A_{average}$ at 450 nm	$[PC^{\bullet+}]$/M	$\Phi_{q,T}$	$\Phi_{q,S}$	$[PC^{\bullet+}]/[PC^*]$ total	$[PC^{\bullet+}]/[PC^*]$ From S_1	$\eta_{ce,S}$	$\eta_{ce,T}$ average
6.2	-7.3×10^{-2}	7.3×10^{-6}	9.84×10^{-3}	7.2×10^{-7}	0.07	0.89	0.099	0.066	0.074	0.07 ± **0.01**
7.7	-9.9×10^{-2}	9.9×10^{-6}	1.18×10^{-2}	8.6×10^{-7}			0.088	0.054	0.060	
9.9	-1.2×10^{-1}	1.2×10^{-5}	1.51×10^{-2}	1.1×10^{-6}			0.094	0.060	0.067	

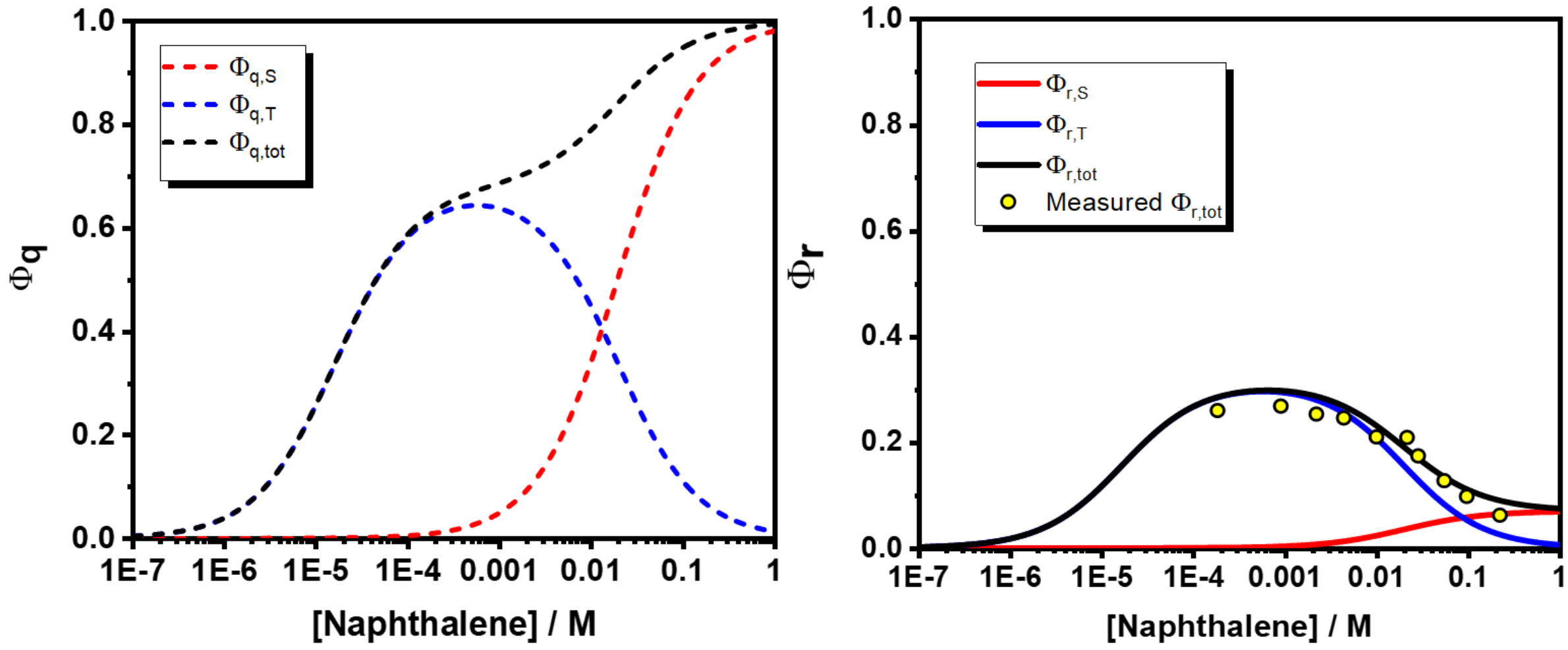

Fig. S25. (Left) Theoretical prediction of radical formation quantum yields based on a quenching-only predictive model. The red and blue lines represent the individual contributions from S_1 and T_1 quenching, respectively, while the black line shows the sum of the two contributions. (Right) Free radical formation quantum yield, accounting for cage escape efficiencies. The red and blue lines correspond to the singlet and triplet channels, respectively, and the black line represents the total free radical formation efficiency. Yellow dots indicate experimentally measured quantum yields of free radical formation over a concentration range of 0.18 mM to 220 mM, calculated from data shown in Fig. S13 (a-j).

S2.8 Kinetic Parameters and η_{ce} with Exact TADF Formalism

In the previous sections (S2.5-S2.7) and in the main text, cage escape measurements were performed by treating the TADF photocatalyst PC^{2+} within the kinetic formalism of conventional chromophores. Within this approximation, the kinetic coupling between the S_1 and T_1 states was neglected. However, in TADF systems, S_1 and T_1 continuously interconvert via intersystem crossing (*isc*) / reverse intersystem crossing (*risc*) cycles. Therefore, equations in section S2.5 are not strictly valid, and a rigorous kinetic framework was employed to validate the cage escape efficiencies previously determined. According to literature reports,[9–11] the correct expressions for the singlet and triplet quantum yields of radical formation are:

$$\Phi_{r,S} = \frac{k_{q,S}[Q]}{k_{fl} + k_{ic} + k_{isc} + k_{q,S}[Q]} \times \eta_{ce,S} \times \frac{1}{1 - \eta_{isc} \times \eta_{risc}}$$

$$\Phi_{r,T} = \frac{k_{isc}}{k_{fl} + k_{ic} + k_{isc} + k_{q,S}[Q]} \times \frac{k_{q,T}[Q]}{k_{ph} + k'_{isc} + k_{risc} + k_{q,T}[Q]} \times \eta_{ce,T} \times \frac{1}{1 - \eta_{isc} \times \eta_{risc}}$$

Where the kinetic constants indicate the following deactivating pathways: $k_{q,s \text{ or } T}$ = quenching from T_1 or S_1, k_{fl} = fluorescence, k_{ic} = internal conversion S_1-S_0, k_{isc} = intersystem crossing S_1-T_1, k_{ph} = phosphorescence, k_{risc} = reverse intersystem crossing T_1-S_1, k'_{isc} = intersystem crossing T_1-S_0.

The factor $\frac{1}{1-\eta_{isc}\times\eta_{risc}}$, not appearing in the equations in section S2.5 accounts for the recursive S_1–T_1 cycling mediated by intersystem crossing and reverse intersystem crossing. Accordingly, radical formation from either state can occur not only after the initial excitation event but also following one or more *isc/risc* cycles prior to quenching. The formulation is further made exact by the explicit inclusion of the unimolecular rate constants governing S_1 and T_1 deactivation, as the expressions:

$$\tau_d = \frac{1}{k_{ph}+k'_{isc}+k_{risc}} \quad \text{and} \quad \tau_p = \frac{1}{k_{fl}+k_{ic}+k_{isc}}$$

are not rigorously applicable, as they do not adequately describe systems with strongly coupled singlet and triplet states.

Instead, we determined the individual unimolecular rate constants by applying the exact mathematical treatment for TADF systems developed in existing literature. [9–11] The kinetic constants were obtained by inserting initial estimates into the full set of equations and iteratively refining them until the calculated output parameters showed optimal agreement with the experimental data (prompt fluorescence lifetime τ_p, delayed fluorescence lifetime τ_d, and ratio of prompt and delayed fluorescence quantum yield Φ_p/Φ_d).

Table S6. Refined kinetic constants of PC^{2+} providing the best agreement between experimental measurements and values calculated using non-approximated TADF kinetic equations.

**Input Kinetic constants**				**Calculated output parameters**			**Experimental parameters**		
$k_{fl}+k_{ic}$	k_{isc}	k_{risc}	$k_{ph}+k'_{isc}$	τ_p calc.	τ_d calc.	Φ_p/Φ_d	τ_p	τ_d	Φ_p/Φ_d
1.90×10^{7}	4.00×10^{7}	4.00×10^{4}	1.00×10^{4}	16.9 ns	43.7 µs	0.84	17 ns	44 µs	0.86

Once the kinetic constants for PC^{2+} were explicitly determined, the correct TADF kinetic equations were used to recalculate the cage-escape efficiencies from the experimental data reported in section S2.6 and S2.7. The results are summarized in Tables S7 and S8.

Table S7. Comparison of cage escape efficiencies obtained from simplified and rigorous TADF kinetic treatments for phenolic quencher.

**Quencher: 4-tbuPhOH**											
**Cage escape measurement for T_1 quenching**											
		**Simplified treatment (section S2.6)**				**Rigorous TADF treatment**					
Laser intensity (mJ/pulse)	[PC•+]/[PC*]	η_{isc}	$\eta_{q,T}$	$\eta_{ce,T}$	**$\eta_{ce,T}$** average	η_{isc}	$\eta_{q,T}$	η_{risc}	$\frac{1}{1-\eta_{isc}\times\eta_{risc}}$	$\eta_{ce,T}$	**$\eta_{ce,T}$** average
5.6	0.25	0.66	0.98	0.39	**0.42 ± 0.02**	0.66	0.95	0.04	1.03	0.39	**0.42 ± 0.02**
6.9	0.27			0.42						0.43	
8.3	0.28			0.43						0.43	
**Cage escape measurement for S_1 quenching**											
		**Simplified treatment (section S2.6)**				**Rigorous TADF treatment**					
Laser intensity (mJ/pulse)	[PC•+]/[PC*]	η_{isc}	$\eta_{q,s}$	$\eta_{ce,T}$	**$\eta_{ce,T}$** average	η_{isc}	$\eta_{q,S}$	η_{risc}	$\frac{1}{1-\eta_{isc}\times\eta_{risc}}$	$\eta_{ce,T}$	**$\eta_{ce,T}$** average
3.6	0.043	0.05	0.94	0.022	**0.028 ± 0.005**	0.04	0.94	<0.01	1.00	0.027	**0.032 ± 0.005**
6.2	0.050			0.030						0.034	
7.7	0.051			0.031						0.036	

Table S8. Comparison of cage escape efficiencies obtained from simplified and rigorous TADF kinetic treatments for naphthalene quencher.

**Quencher: Naphtalene**											
**Cage escape measurement for T_1 quenching**											
		**Simplified treatment (section S2.7)**				**Rigorous TADF treatment**					
Laser intensity (mJ/pulse)	[PC•+]/[PC*]	η_{isc}	$\eta_{q,T}$	$\eta_{ce,T}$	$\eta_{ce,T}$ average	η_{isc}	$\eta_{q,T}$	η_{risc}	$\frac{1}{1-\eta_{isc}\times\eta_{risc}}$	$\eta_{ce,T}$	$\eta_{ce,T}$ average
5.3	0.32	0.69	0.90	0.51	**0.46 ± 0.03**	0.67	0.81	0.15	1.11	0.52	**0.47 ± 0.03**
6.6	0.27			0.44						0.45	
7.5	0.29			0.46						0.47	
9.1	0.27			0.44						0.45	
**Cage escape measurement for S_1 quenching**											
		**Simplified treatment (section S2.7)**				**Rigorous TADF treatment**					
Laser intensity (mJ/pulse)	[PC•+]/[PC*]	η_{isc}	$\eta_{q,s}$	$\eta_{ce,T}$	$\eta_{ce,T}$ average	η_{isc}	$\eta_{q,S}$	η_{risc}	$\frac{1}{1-\eta_{isc}\times\eta_{risc}}$	$\eta_{ce,T}$	$\eta_{ce,T}$ average
6.2	0.099	0.07	0.89	0.074	**0.07 ± 0.01**	0.07	0.89	<0.01	1.00	0.073	**0.07 ± 0.01**
7.7	0.088			0.060						0.060	
9.88	0.094			0.067						0.067	

S2.9 Transferring Spectroscopic predictions to continuous irradiation (University of Bologna)

Up to this point, we have discussed only results obtained from pulsed experiments, in which the system is subjected to an intense but very short light pulse. Under these conditions, the composition of the system remains essentially unchanged: no species are significantly depleted relative to their initial concentrations. On the other hand, in a photoreaction the system is generally kept under irradiation for several hours, during which the reacting environment changes: even in the best-case scenario, where no side reactions are considered, the quencher (Q) will be depleted during reaction progression. This introduces a fundamental discrepancy between pulsed experiments – where only instantaneous quantum yields can be measured – and reaction outcomes – where the photoreaction quantum yield is measured in a larger time-span and is also affected by the evolution of the system under analysis. We address this discrepancy in this section and in the main text by means of a kinetic model developed in a previous work by the Ceroni group.[11]

Mechanistic Model *a*

According to ref. [11], we considered both mechanisms that can occur in a photoreaction. As for mechanism a, we consider a cycle as depicted in Fig. S26, where all the reaction steps after the production of $PC^{•+}$ are quantitative.

In this framework, the model is able to predict how the photoreaction quantum yield changes as a result of Q depletion. The magnitude of Q depletion depends on the ratio between the amount of quencher and the amount of substrate used: If [Q] ≫ [S], the reaction will end up with a minimal amount of quencher consumed, and therefore the photoreaction quantum yield will likely remain fairly constant during time. On the other hand, if [Q]~[S], the photoreaction quantum yield can significantly change during reaction.

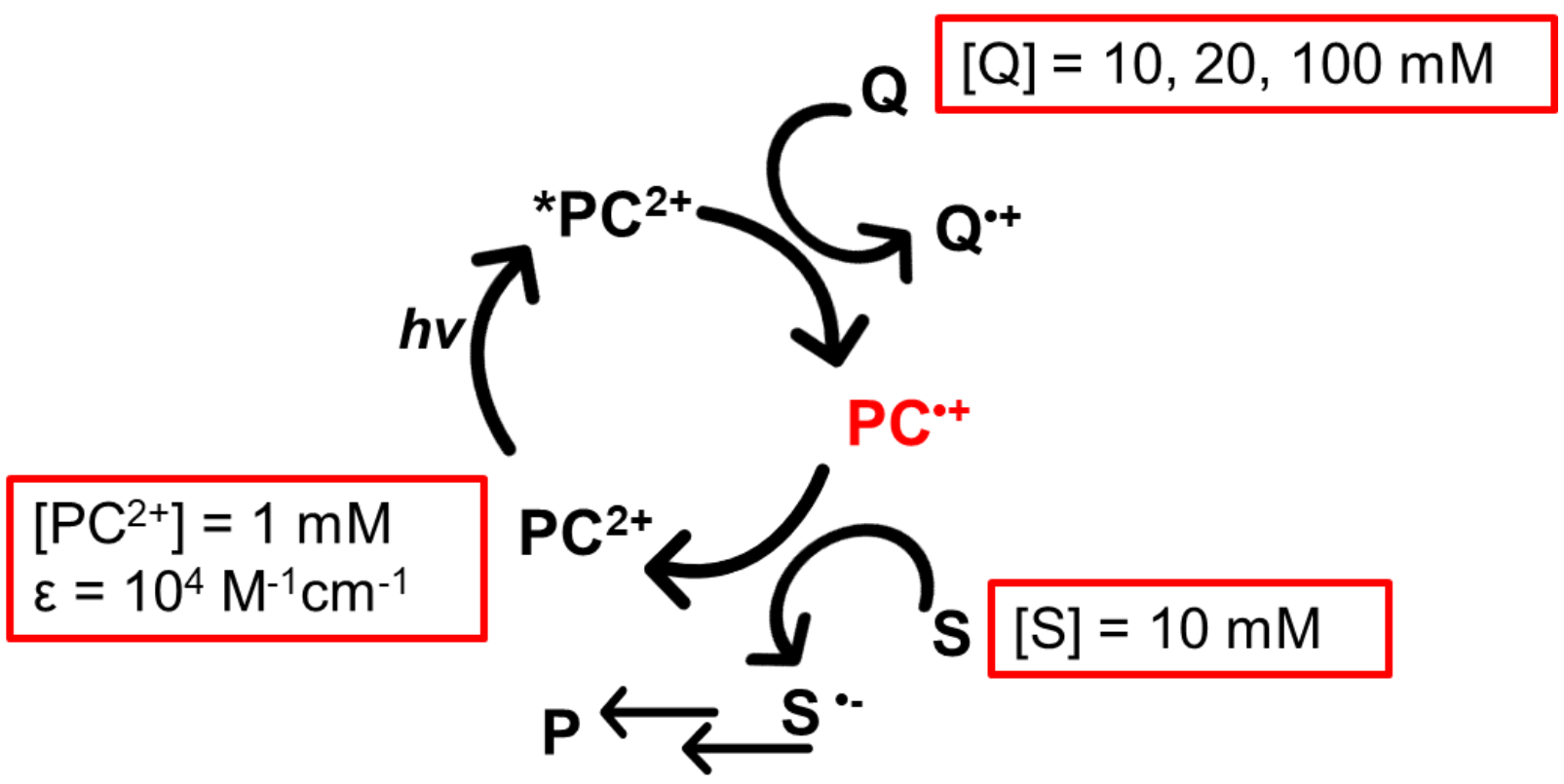

Fig. S26. Mechanistic model ***a*** of the photoreaction used to investigate the photoreaction efficiency under continuous irradiation. Q represents the sacrificial quencher that reduces the photocatalyst, while S is the reaction substrate that accepts an electron from the reduced photocatalyst and subsequently undergoes a dark reaction pathway leading to the formation of product P.

In the model, the amount of product formed is calculated along reaction progression, and "the end of the photoreaction" is considered as the instant t_{fin} at which $[S]_{fin} = 0.01\ [S]_0$,

meaning that 99% of the substrate is converted to product. Then, the overall photoreaction quantum yield can be calculated at the end of the photoreaction as the ratio between the final concentration of product and the total amount of photons absorbed during the whole process. The model allows to calculate the photoreaction quantum yield for a series of starting concentrations of the quencher, by using the following input parameters:

```
  #insert the values of all the kinetic constants

kS0 = 1.9E7
kisc = 4e7
kT0 = 1e4
krisc = 4e4
kqS = 6.6e9
kqT = 4.5e9
ceS = 0.03 #cage escape efficiency of S1
ceT = 0.42 #cage escape efficiency of T1

#Additional input values
A0 = 0.01 #initial concentration of the substrate in M
S0 = 0.001 #initial concentration of the sensitizer in M
eps = 1e4 #molar absorptivity of the sensitizer in M^-1 cm^-1
I0 = 1e-3 #incident photon flux in mol/s
f = 0.99 #fraction substrate consumption that we consider "the end of the reaction"

#Insert the values of [Q] to be analysed
Q0_min = A0*1 #cannot be lower than the initial concentration of the substrate
Q0_max = 1
```

The input parameters were chosen in order to mimic the system under analysis in this study: all the kinetic constants and efficiencies are taken as those measured in pulsed measurements, while the photon flux and initial concentration of the photocatalyst are chosen to mimic real photochemical set-ups. The range of quencher concentrations scanned is chosen to match the experimentally measured ones, as depicted in Fig. 4 in the main text. To obtain meaningful data, the substrate concentration is fixed at 10 mM. Consequently, quencher concentrations below 10 mM could not be considered, since for one-electron transfer reactions the minimum feasible Q ratio is 1:1, reflecting the reaction stoichiometry.

The $\Phi_{reaction}$ values obtained with the kinetic model can be compared to the same values obtained on the basis of instantaneous efficiencies, directly measured in pulsed experiments (Fig. S27). As mentioned above, instantaneous efficiencies do not account for the evolution of the system during photoreaction, and therefore it is interesting to compare the two curves to understand how the results found in pulsed experiments would translate in a real photoreaction.

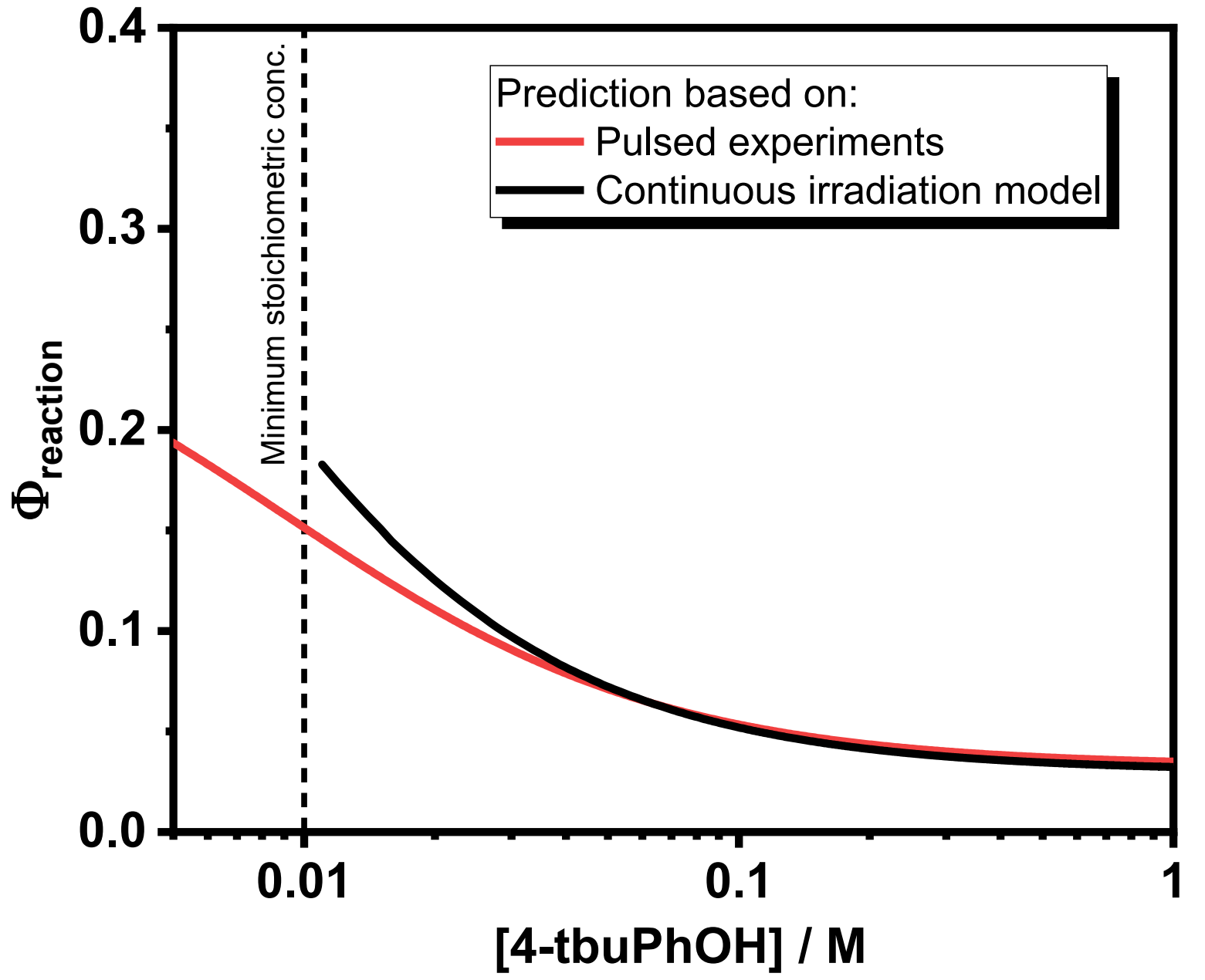

Fig. S27. Comparison of the predicted photoreaction quantum yield obtained from pulsed experiments and simplified kinetic equations with that derived from the rigorous kinetic treatment implemented in the Python code under continuous irradiation conditions. The minimum quencher concentration required to achieve complete substrate conversion (10 mM) is highlighted**.**

The two curves show very similar trends, with $\Phi_{reaction}$ decreasing upon increasing the initial concentration of the quencher. This validates the results obtained with pulsed experiments: the counterintuitive trend “less is more” should be expected also for real photoreactions based on the same system. However, a discrepancy is observed at low quencher concentrations (10-30 mM). This can be rationalized by considering that the initial substrate concentration was fixed at [S] = 10 mM. Consequently, when the initial quencher concentration approaches that of the substrate, a substantial fraction of the quencher is consumed over the course of the photoreaction. For example, if the initial quencher concentration is $[4\text{-tbuPhOH}]_0$ = 20 mM, complete conversion of the substrate would result in a final quencher concentration of $[4\text{-tbuPhOH}]_{fin}$ = 20 - [S]*0.99 ≈ 10.01 mM. Such a large decrease in quencher concentration causes the system to progressively evolve toward conditions of higher T_1 reactivity, ultimately leading to an increase in the overall photoreaction quantum yield. In contrast, when [Q] ≫ [S] (e.g., [Q] = 1 M), only a negligible fraction of the quencher is consumed during the reaction. Under these conditions, the quencher concentration remains effectively constant, and the results closely resemble those obtained in pulsed experiments. Therefore, this comparison demonstrates that the "less is more" trend is preserved upon moving from pulsed to continuous irradiation and, if anything, becomes even more pronounced under continuous irradiation conditions.

We chose three concentrations of the quencher in the explored range to analyze how $\Phi_{reaction}$ would evolve in a real photoreaction (Fig. 5 in the main text). The input parameters for modelling the photoreaction used to generate Fig. 5 in the main text are as follows:

```
mechanism_type = 'a' #options: 'a', 'b'

#insert the values of all the kinetic constants
kS0 = 1.9e7
kisc = 4e7
kT0 = 1e4
krisc = 4e4
kqS = 6.6e9
kqT = 4.5e9
ceS = 0.03 #cage escape efficiency of S1
ceT = 0.42 #cage escape efficiency of T1

#Additional input values
A0 = 0.01 #initial concentration of the substrate in M
S0 = 0.001 #initial concentration of the sensitizer in M
Q0 = SPANNING FROM 0.01  TO 0.2 #initial concentration of the sacrificial donor in
M - not relevant in mechanism b
eps = 1e4 #molar absorptivity of the sensitizer in M^-1 cm^-1
I0 = 1e-3 #incident photon flux in mol/s
f = 0.99 #fraction substrate consumption that we consider "the end of the reaction"
```

Mechanistic Model *b*

The dependence of the photoreaction quantum yield on quencher concentration and its variation over reaction progress can also be investigated using an alternative mechanistic model:

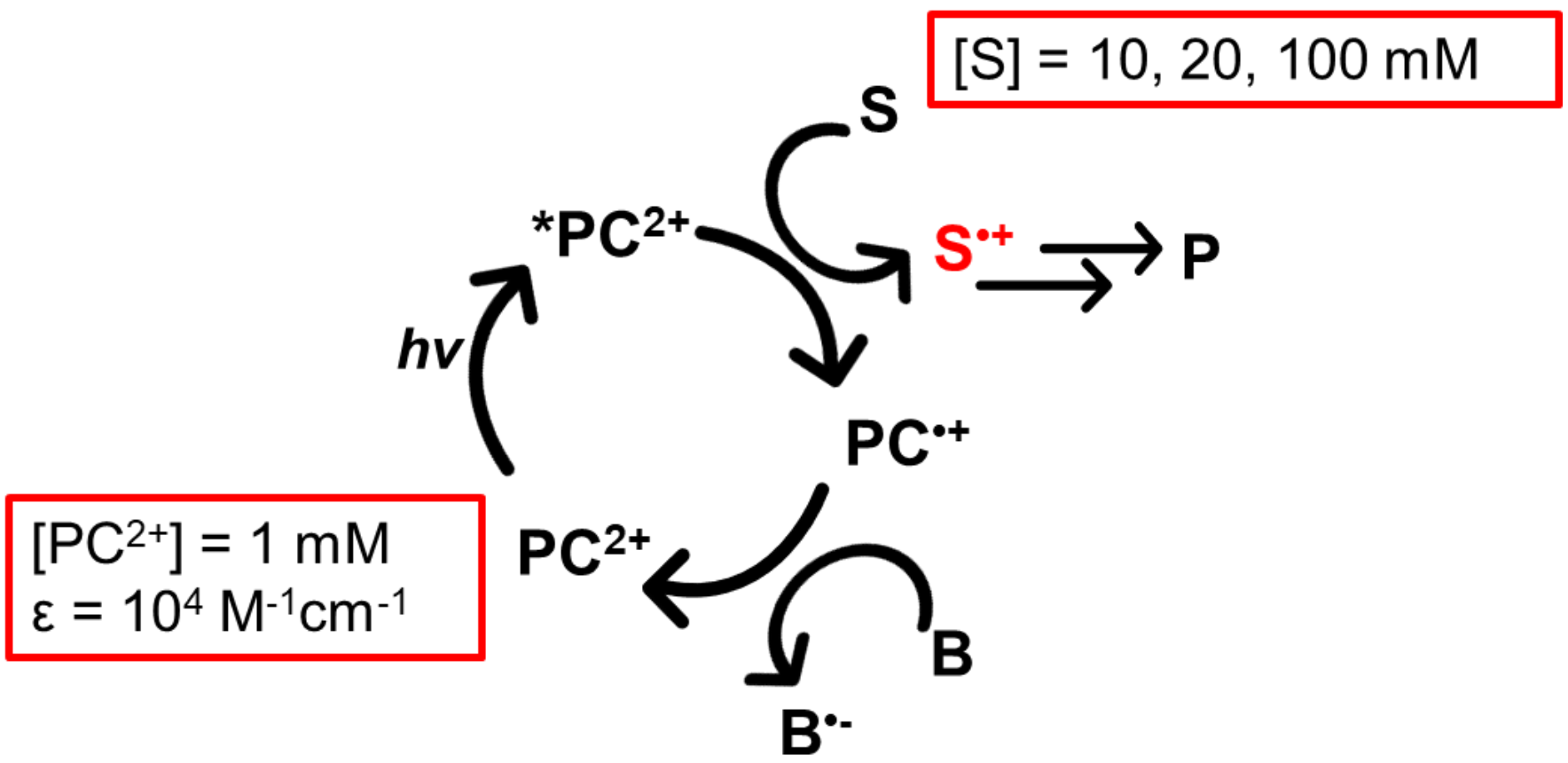

Fig. S28. Mechanistic model ***b*** for photoreaction was used to investigate the photoreaction efficiency under continuous-wave irradiation. S represents both the quencher undergoing photoinduced electron transfer to the excited photocatalyst and the substrate involved in the subsequent dark processes leading to the final product P; B represents a sacrificial electron acceptor that regenerates the photocatalyst to its original state, enabling a new catalytic cycle.

The photoreaction quantum yield is calculated as explained previously for mechanism **a**. Analogously, this model assumes that processes following $S^{\bullet+}$ formation occur with unitary efficiency. The only difference in this mechanism is that in this case the quencher is the

substrate, meaning that its concentration will surely change during reaction, as we want its almost complete conversion into the product. Also in this case, we consider as the end of the photoreaction the instant t_{fin} in which $[S]_{fin} = 0.01*[S]_0$.

The input parameters for modelling the photoreaction are as follows:

```
mechanism_type = 'b' #options: 'a', 'b'

#insert the values of all the kinetic constants
kS0 = 1.9e7
kisc = 4e7
kT0 = 1e4
krisc = 4e4
kqS = 6.6e9
kqT = 4.5e9
ceS = 0.03 #cage escape efficiency of S1
ceT = 0.42 #cage escape efficiency of T1

#Additional input values
A0 = SPANNING FROM 0.01 TO 0.1 #initial concentration of the substrate in M
S0 = 0.001 #initial concentration of the sensitizer in M
Q0 = 0.02 #initial concentration of the sacrificial donor in M - not relevant in
mechanism b
eps = 1e4 #molar absorptivity of the sensitizer in M^-1 cm^-1
I0 = 1e-3 #incident photon flux in mol/s
f = 0.99 #fraction substrate consumption that we consider "the end of the reaction"
```

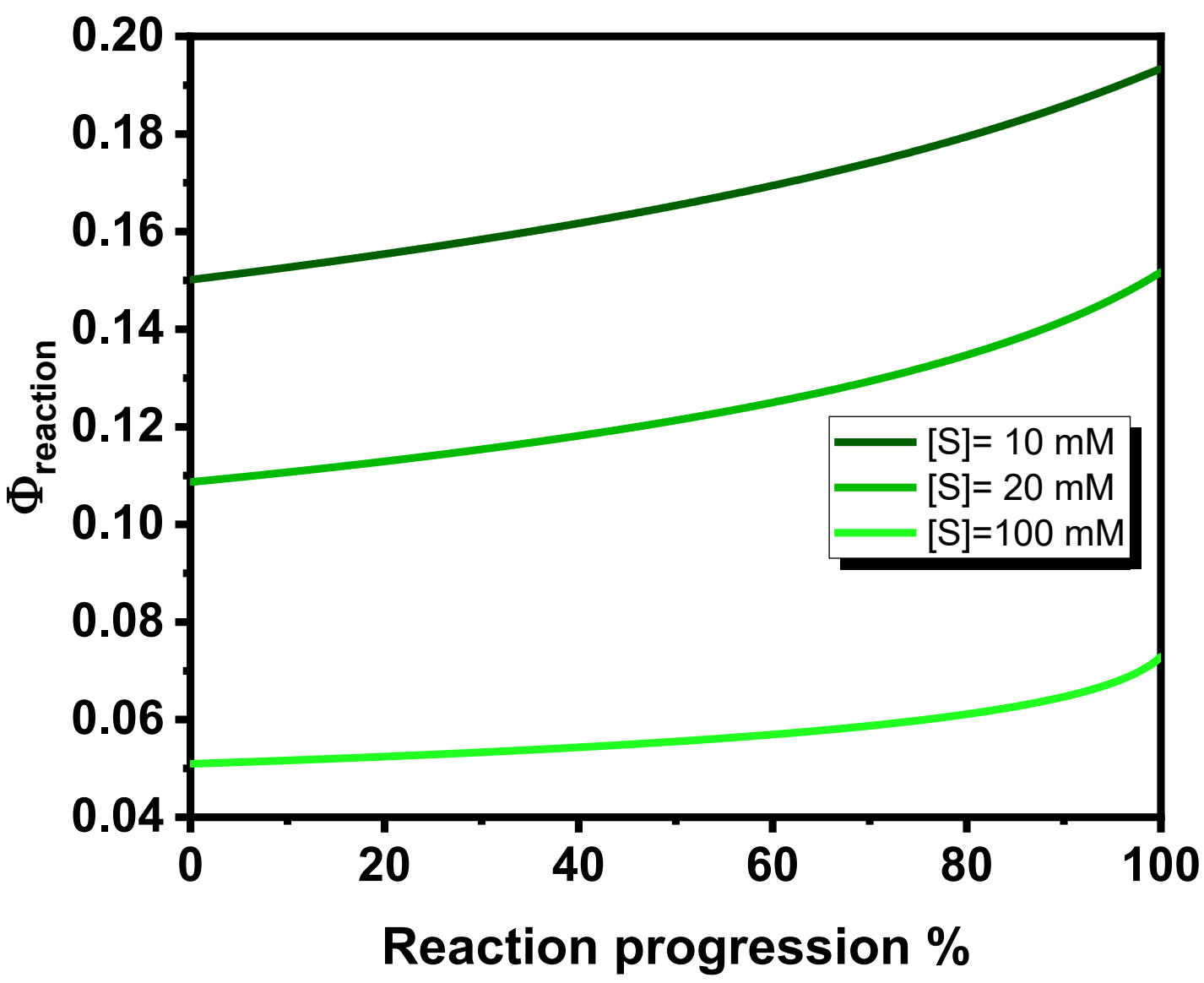

Fig. S29. Predicted maximum photoreaction quantum yield vs reaction progress with three initial quencher concentrations in a hypothetical photoreaction following mechanism ***b*** (Fig. S28). The x-axis is normalized to the total reaction time to allow direct comparison.

Fig. S29 illustrates a situation analogous to that shown in Fig. 5, where higher quencher concentrations result in substantially lower overall photoreaction quantum yields that evolve over the course of the reaction. In this case, however, the dependence of $\Phi_{reaction}$ on reaction progress is considerably more pronounced. This behavior can be rationalized by considering that the quencher – which in this system is the substrate itself – undergoes complete conversion to the product, such that its concentration varies substantially throughout the entire reaction. In contrast to the scenario discussed previously, in which high quencher concentrations could be approximated as constant over the reaction timescale, this assumption is never valid here. Consequently, the system undergoes more significant changes over the course of the reaction, accounting for the more marked improvement in photoreaction quantum yield observed in Fig. S29.

S3. "Less is More" Effect for Further Photocatalysts

S3.1 4CzIPN

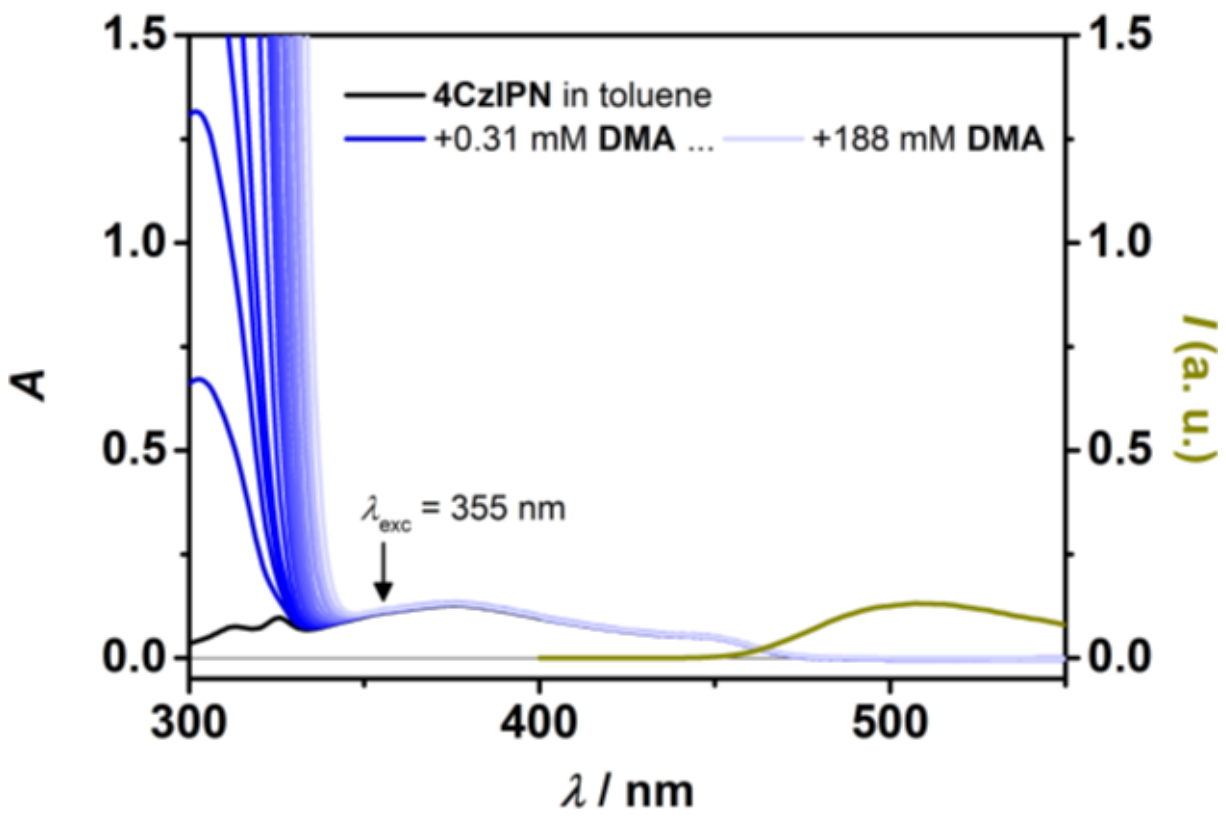

Fig. S30. UV-vis absorption (black) and emission (yellow) spectra of 4CzIPN in toluene as well as UV-vis absorption spectra of solutions containing 4CzIPN in the presence of different concentrations of DMA (0.31–188 mM, blue) along with the indication of the excitation wavelength for nanosecond transient absorption spectroscopy.

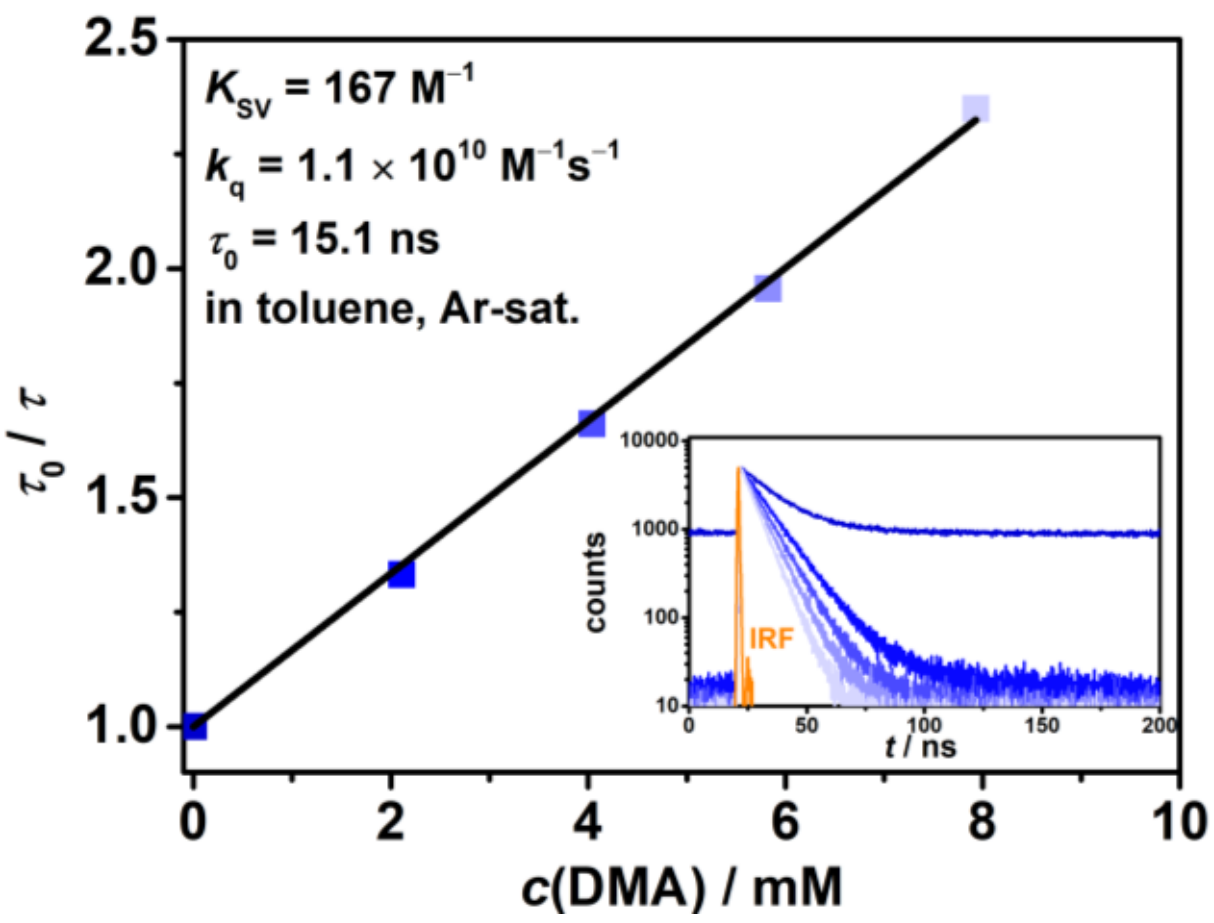

Fig. S31. Stern–Volmer plot of S_1 quenching of 4CzIPN by DMA in Ar-saturated toluene using TCSPC (see inset for raw data sets, λ_{exc} = 371 nm, λ_{det} = 517.5–522.5 nm). The elevated baseline in the unquenched solution is due to the delayed emission under the selected oxygen-free conditions.

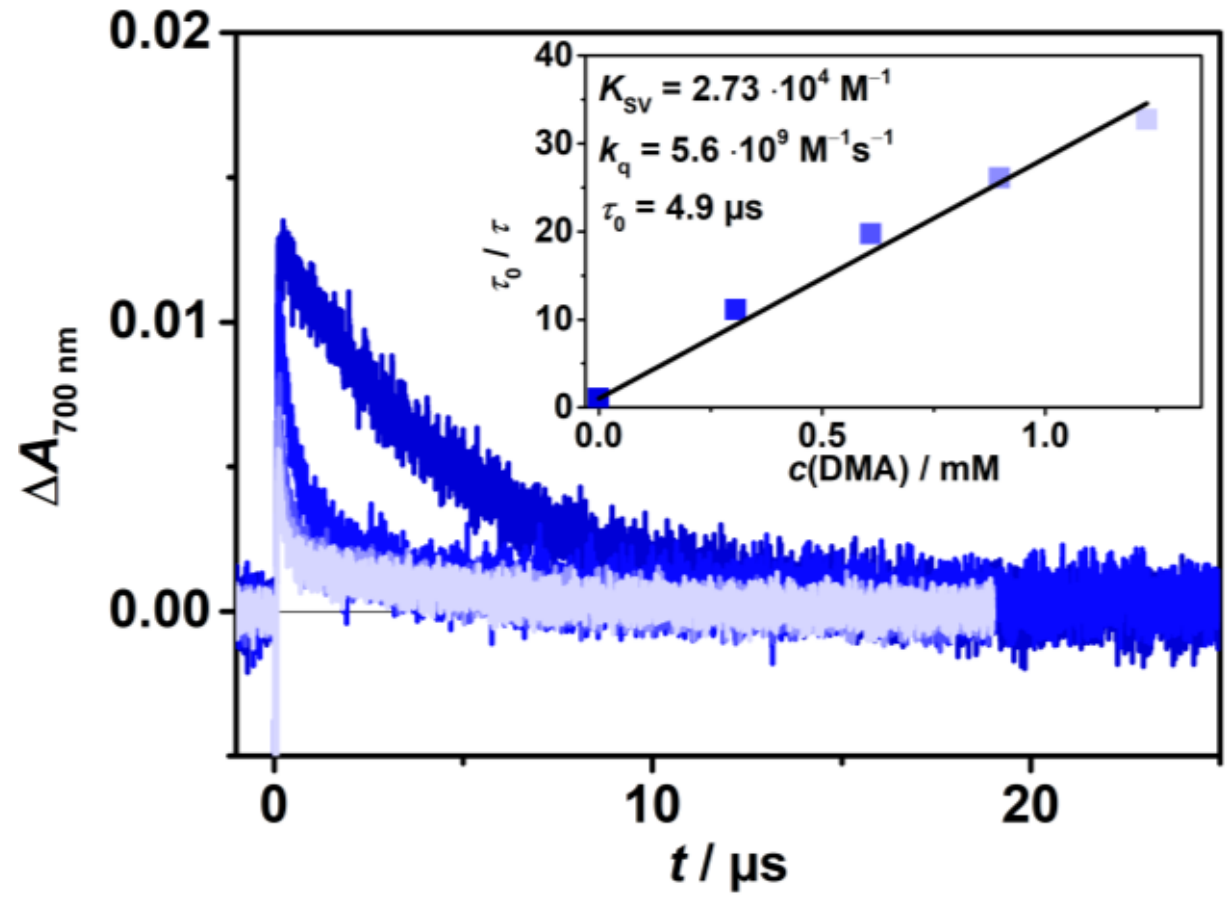

Fig. S32. Time-resolved transient absorption traces after laser excitation displaying T_1 quenching of 4CzIPN by DMA in Ar-saturated toluene (see inset for corresponding Stern–Volmer plot, λ_{exc} = 355 nm (10 mJ/pulse), λ_{det} = 700 nm).

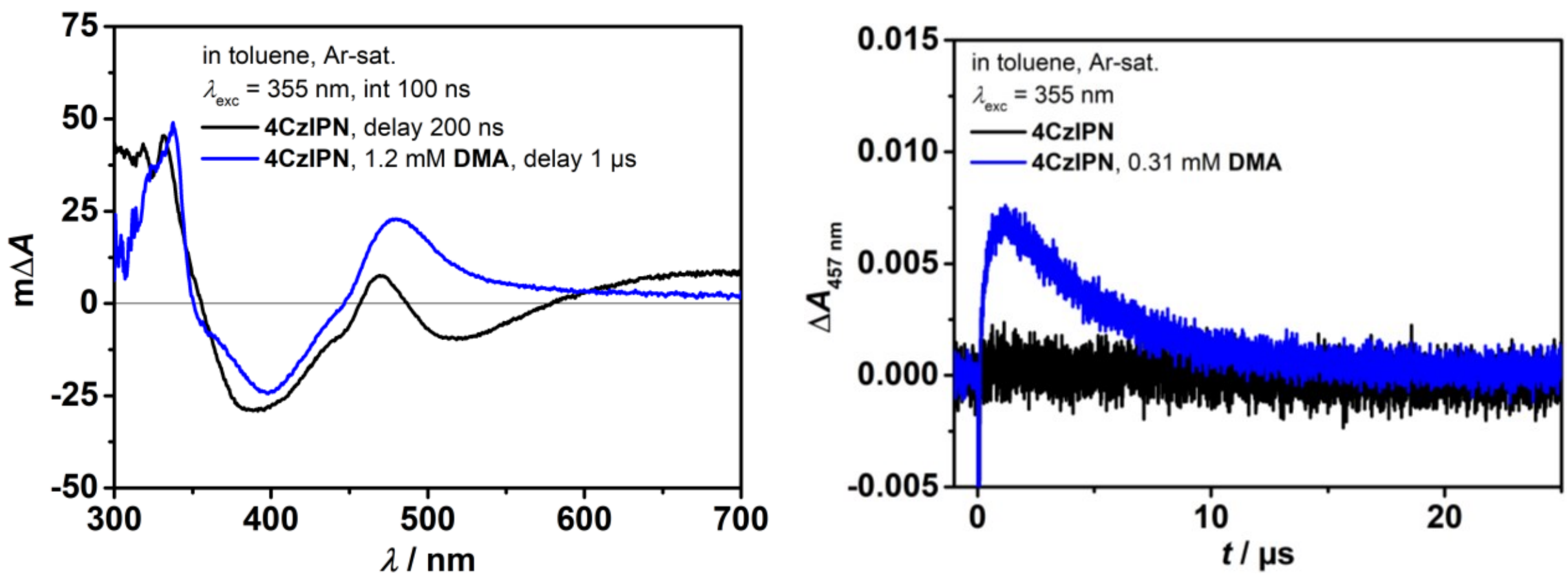

Fig. S33. (Left) Transient absorption spectra of Ar-saturated solutions containing 4CzIPN without or with 1.2 mM DMA in toluene after laser excitation (λ_{exc} = 355 nm, delay times are indicated in the figure) displaying ^{3}4CzIPN (black) or $4CzIPN^{\bullet-}$ (blue). (Right) Time-resolved transient absorption traces after laser excitation of Ar-saturated solutions containing 4CzIPN without or with 0.31 mM DMA in toluene after laser excitation (λ_{exc} = 355 nm, 10 mJ/pulse) showing the recombination of $4CzIPN^{\bullet-}$ and $DMA^{\bullet+}$ with a time constant of ~4 µs.

After quenching of ^{3}4CzIPN by DMA, a transient absorption spectrum is obtained that is characteristic for the formation of $4CzIPN^{\bullet-}$.[12] Besides that, $DMA^{\bullet+}$ is formed in stoichiometric amounts, which, however, possesses an absorption in the visible region (~450 nm) with a much lower extinction coefficient[13] and it is therefore most likely masked by much more pronounced absorption changes caused by $4CzIPN^{\bullet-}$ formation.

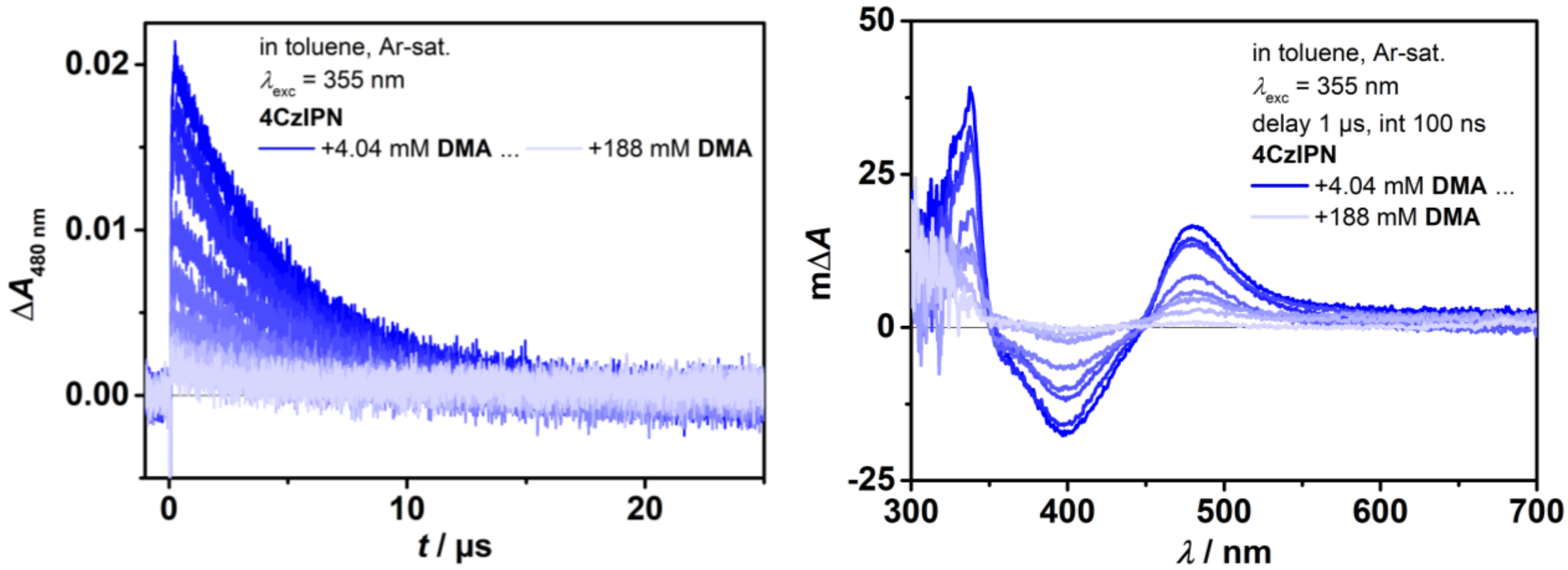

Fig. S34. (Left) Time-resolved transient absorption traces after laser excitation of Ar-saturated solutions containing 4CzIPN with different amounts of DMA in toluene after laser excitation (λ_{exc} = 355 nm, 10 mJ/pulse). (Right) Transient absorption spectra of the same solutions after laser excitation (λ_{exc} = 355 nm, 10 mJ/pulse).

The initial amplitude of the transient absorption signal shown in Fig. S34 (left), which is directly proportional to the radical formation upon photoexcited 4CzIPN quenching, was determined by an exponential fit. The DMA concentration-dependent relative amplitudes are shown in Fig. 6 in the main part. For the fit to determine the ratio between $\eta_{ce,S}$ and $\eta_{ce,T}$ shown in Fig. 6C, k_{isc} (= $\Phi_{isc} \times k_{0,S}$) was calculated based on the known Φ_{isc} = 0.73 in toluene.[14]

S3.2 SO_2TDS^{2-}

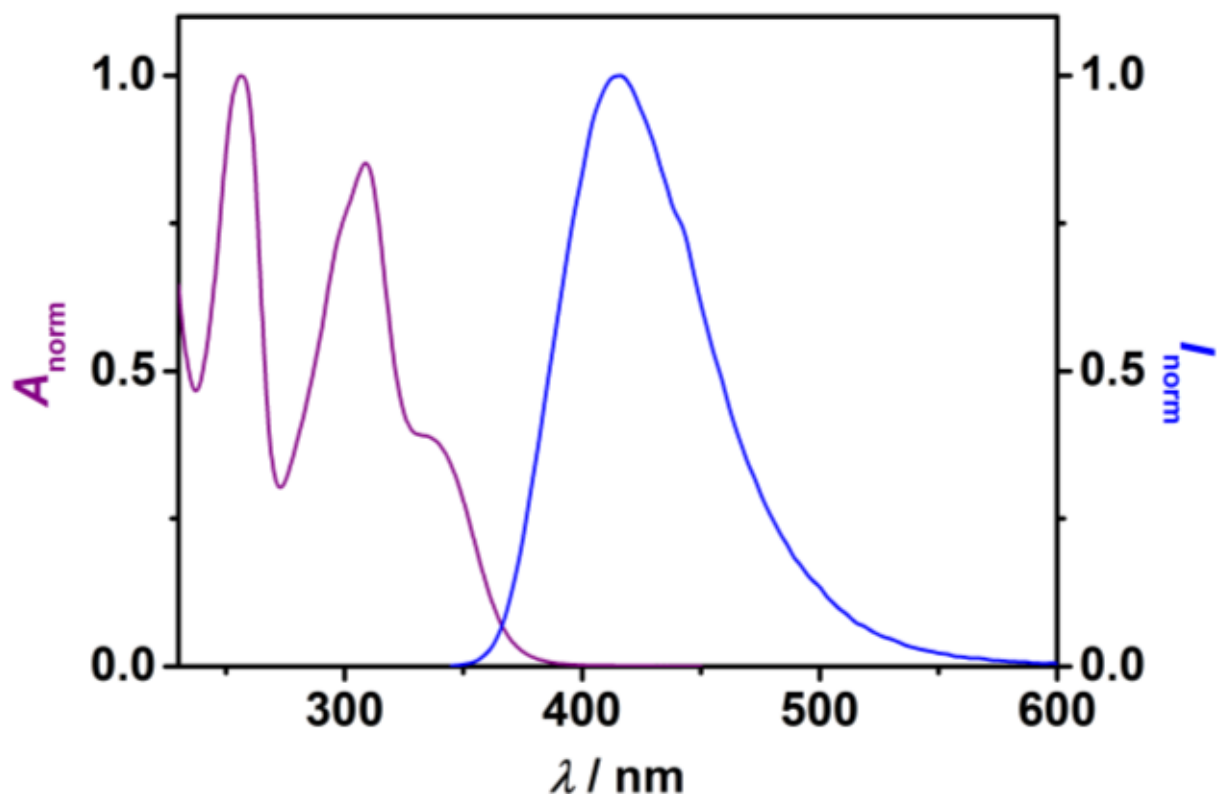

Fig. S35. UV-vis absorption (violet) and emission (blue) spectra of SO_2TDS^{2-} in an aqueous phosphate buffer (200 mM, pH = 7.2).

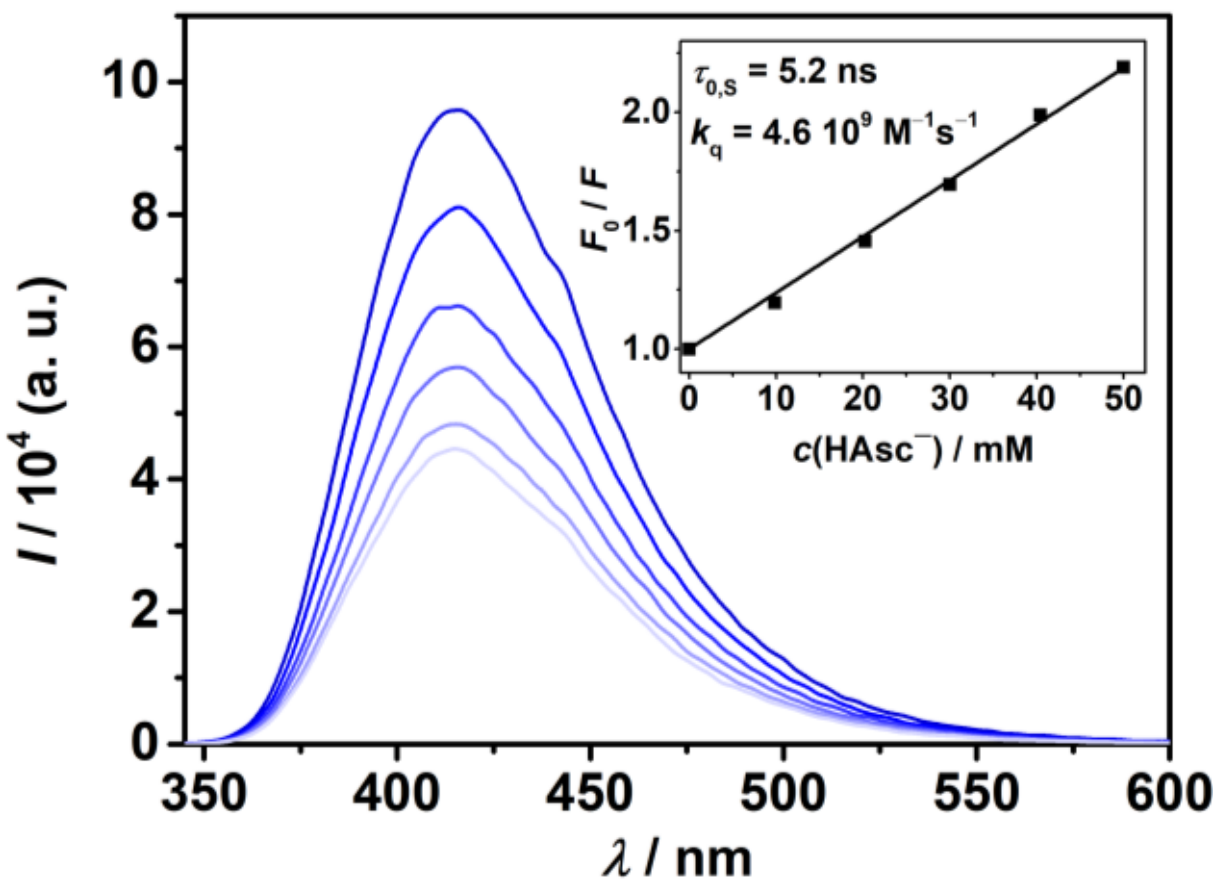

Fig. S36. Steady-state emission spectra of Ar-saturated aqueous solutions (200 mM phosphate buffer, pH = 7.2) containing SO_2TDS^{2-} (13 μM) and different amounts of NaHAsc (λ_{exc} = 335 nm). Inset: Corresponding Stern–Volmer plot along with the calculated dynamic quenching rate constant k_q (using the known unquenched S_1 lifetime of 5.2 ns)[2].

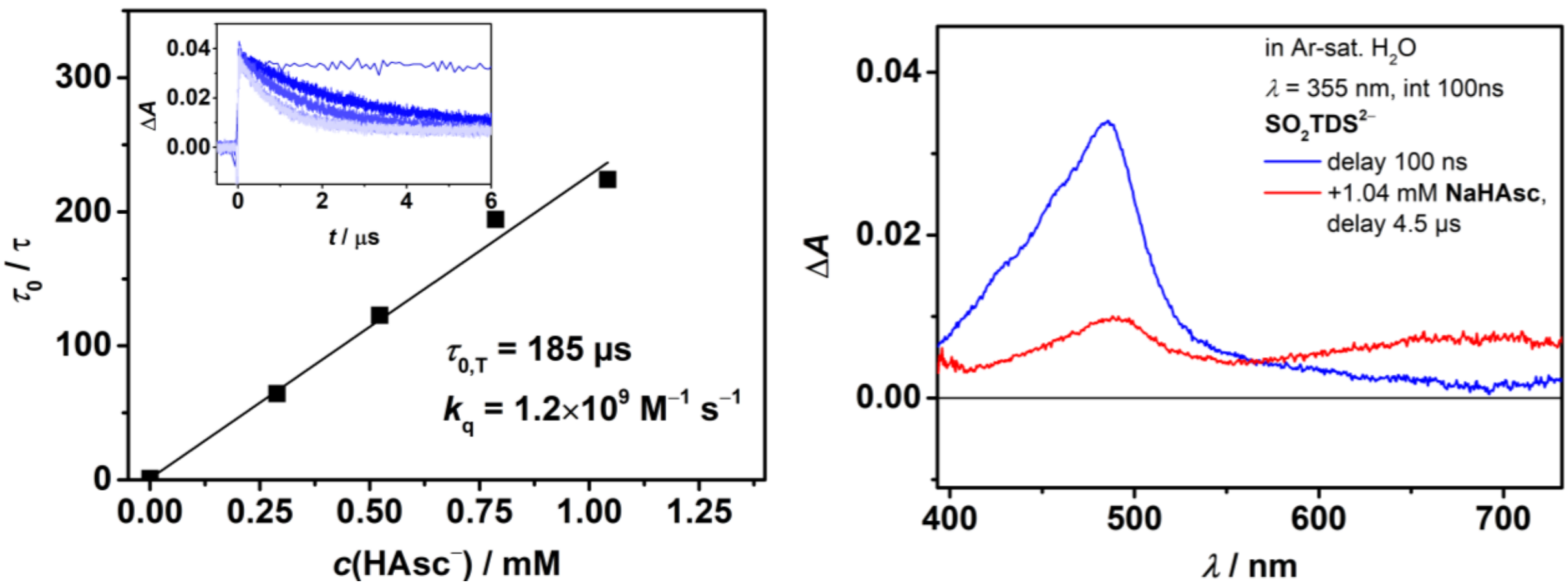

Fig. S37. (Left) Stern–Volmer plot of the T_1 quenching of SO_2TDS^{2-} by $HAsc^-$ in Ar-saturated aqueous solution (200 mM phosphate buffer, pH = 7.2, see inset for time-resolved transient absorption traces after laser excitation (λ_{exc} = 355 nm 15 mJ/pulse, λ_{det} = 485 nm). (Right) Transient absorption spectra of Ar-saturated solutions containing SO_2TDS^{2-} without or with 1.04 mM $HAsc^-$ aqueous solution (200 mM phosphate buffer, pH = 7.2) after laser excitation (λ_{exc} = 355 nm, 15 mJ/pulse), delay times are indicated in the figure displaying $^3SO_2TDS^{2-}$ (blue) or $SO_2TDS^{\bullet 3-}$ (red).

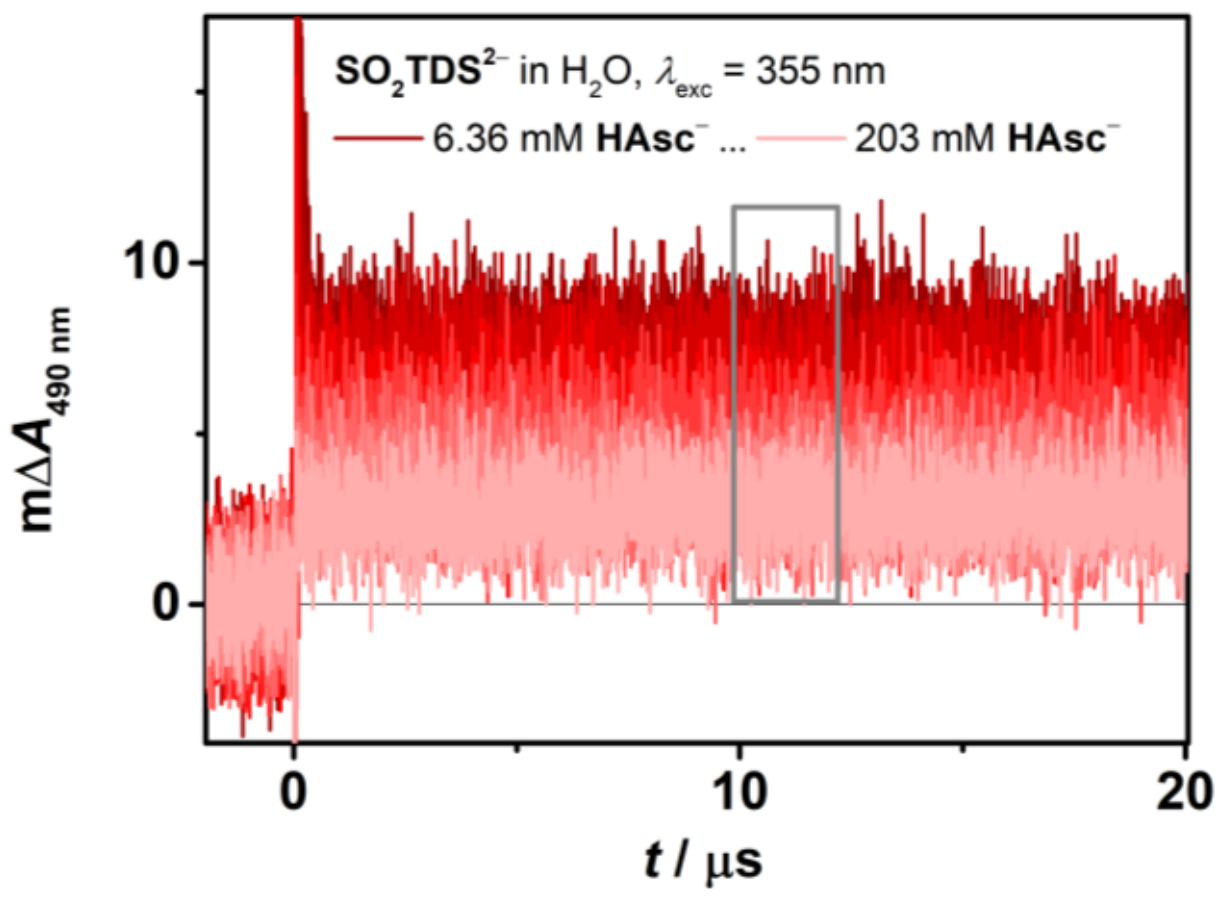

Fig. S38. Time-resolved transient absorption traces after laser excitation of Ar-saturated aqueous solutions (200 mM phosphate buffer, pH = 7.2) containing SO_2TDS^{2-} with different amounts of $HAsc^-$ after laser excitation (λ_{exc} = 355 nm, 15 mJ/pulse). The gray box indicates the time interval for data averaging to determine ΔA value at a given $HAsc^-$ concentration (see Fig. 6 C for relative $HAsc^-$ concentration-dependent ΔA or radical yield).

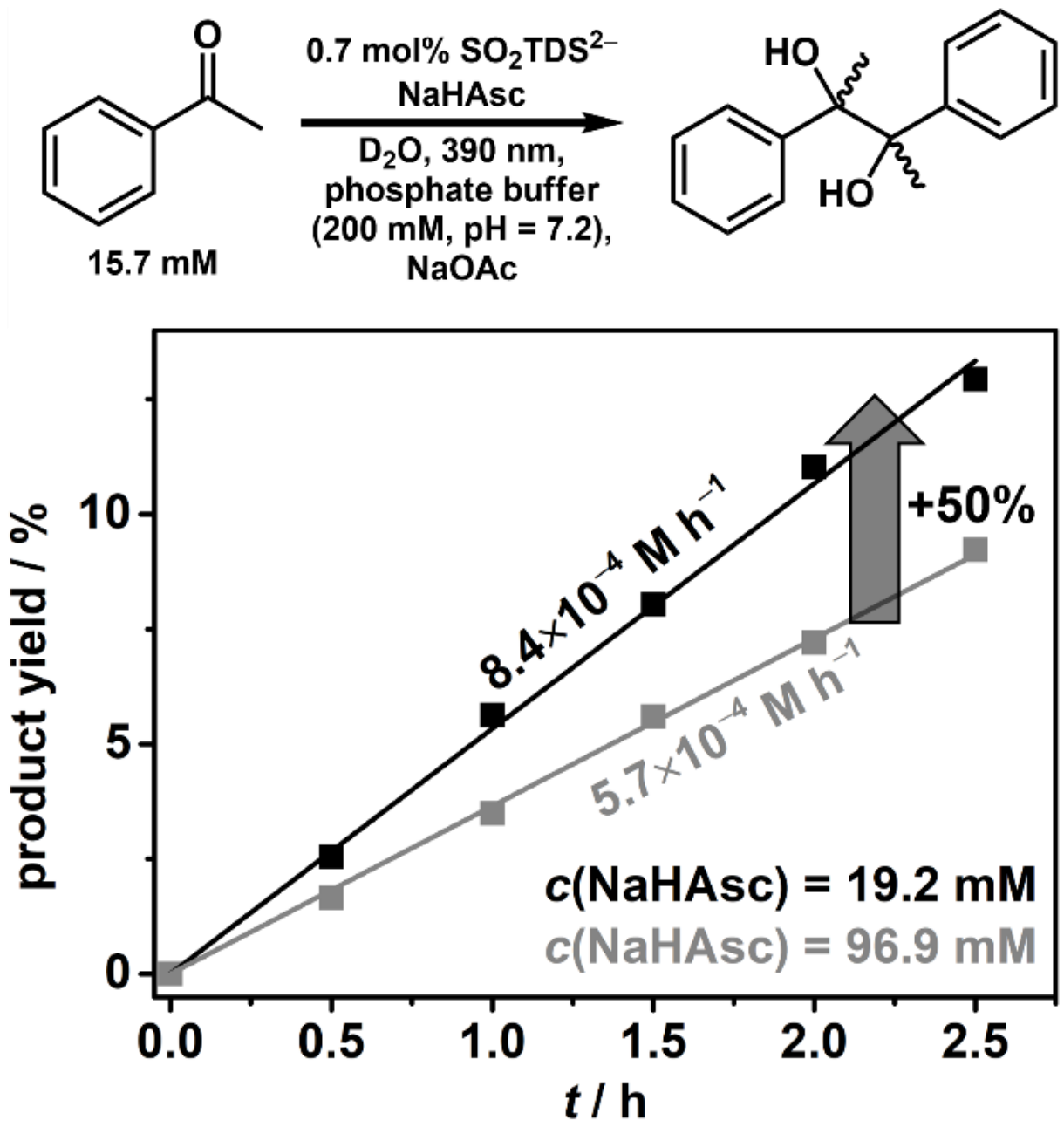

Fig. S39. (Top) Chemical equation of the pinacol coupling of acetophenone using SO_2TDS^{2-} as photocatalyst under 390 nm LED irradiation (Ar-saturated D_2O solution in the presence of 200 mM phosphate buffer (pH = 7.2) and NaOAc (13.0 mM) as internal reference). (Bottom) Time-resolved product yield using low (19.2 mM) and high (96.9 mM) concentrations of the sacrificial electron donor $HAsc^-$, along with linear fitting functions to determine the respective slopes. See Fig. S40 and S41 for corresponding ^1H NMR spectra.

To confirm the "less is more" effect under lab-scale conditions, irradiation experiments (390 nm LED) were carried out using SO_2TDS^{2-} as photocatalyst and $HAsc^-$ as reductive quencher for the pinacol coupling of acetophenone (see Fig. S39). In the underlying mechanism, photoexcited SO_2TDS^{2-} is reductively quenched by $HAsc^-$, leading to the formation of $SO_2TDS^{\bullet 3-}$, which is capable of reducing acetophenone to its ketyl radical anion. Two reduced acetophenone species subsequently undergo C–C bond formation, which, after protonation, results in the pinacol product shown in Fig. S39. As presented earlier in this section and in Fig. 6 B/C in the main part, higher concentrations or a large

excess of the reductive quencher $HAsc^{-}$ can result in significantly lower concentrations of the key species $SO_2TDS^{\bullet 3-}$ and thus affect the performance of a photocatalytic reaction. Thus, an irradiation experiment was carried out with a lower $HAsc^{-}$ concentration (19.2 mM, 1.2 eq.), which represents a sufficient concentration to achieve a complete conversion,[2] and again with a high $HAsc^{-}$ concentration (96.9 mM, 6.2 eq.) under otherwise identical conditions. Fig. S39 shows the time-resolved product yields of both irradiation experiments, which were kept below 15% to remain in the linear regime. The rates of the reaction progress were determined using linear fitting functions. Based on this, the reaction performed ~50% better with a low $HAsc^{-}$ concentration than with a larger excess of the sacrificial electron donor. This aligns well with the spectroscopic investigations, which show a ~60% increase in $SO_2TDS^{\bullet 3-}$ formation for the low $HAsc^{-}$ concentration (19.2 mM) relative to the high $HAsc^{-}$ concentration (96.9 mM) (see Fig. 6 C in the main part), strongly indicating that the "less is more" effect is operative in lab-scale irradiation experiments.

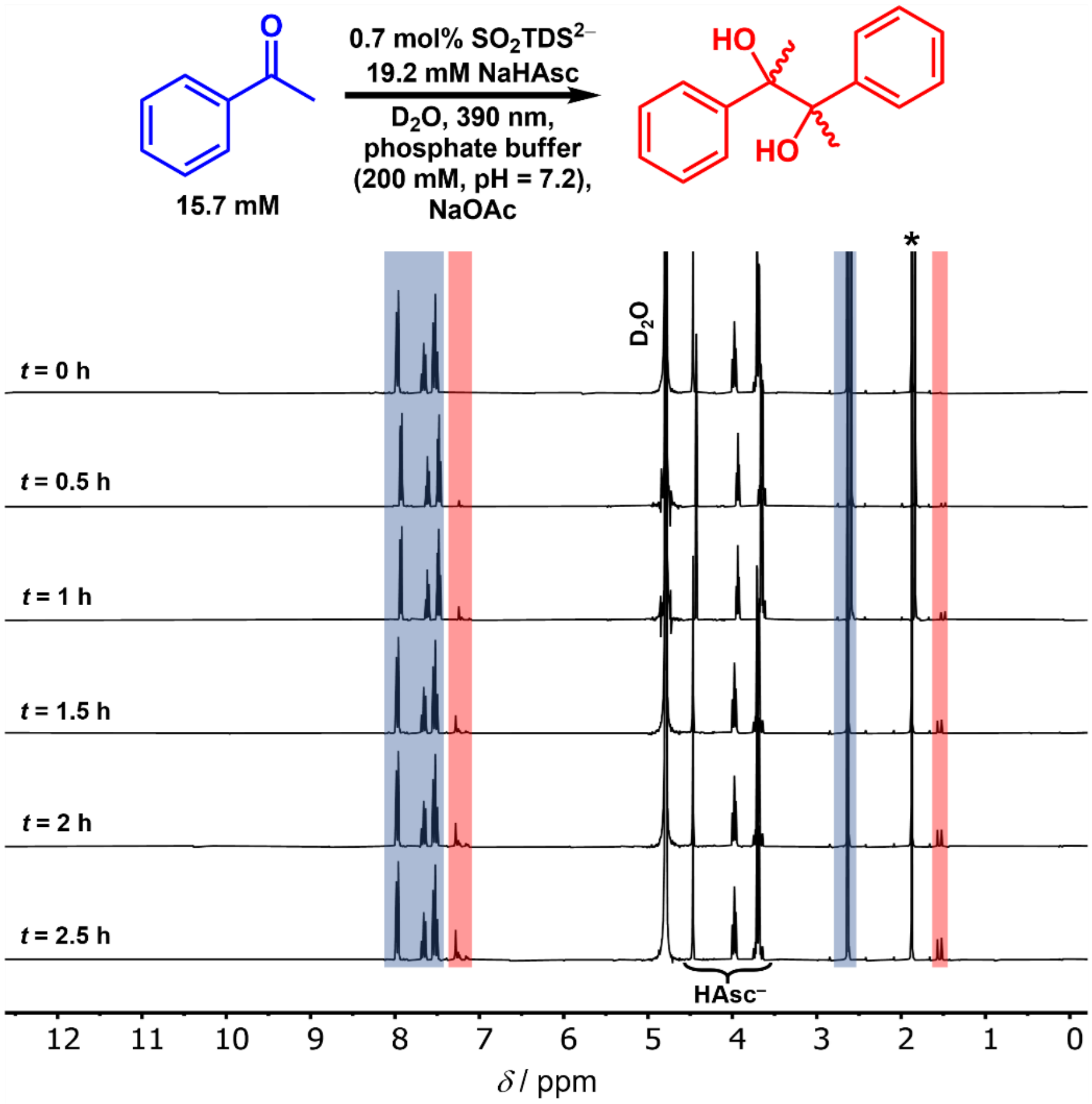

Fig. S40. ^{1}H NMR spectra of the pinacol coupling of acetophenone using SO_2TDS^{2-}, a 390 nm LED, and a lower concentration of NaHAsc (19.2 mM, 1.2 eq.) in Ar-saturated D_2O (200 mM phosphate buffer, pH = 7.2). The product formation was quantified using the CH_3 proton signals (at $\delta \sim$ 1.5 ppm, the two signals correspond to the D/L and the meso forms of the product) relative to the internal standard (NaOAc, assigned with an asterisk). See also Fig. S39 for the resulting time-resolved product formation plot.

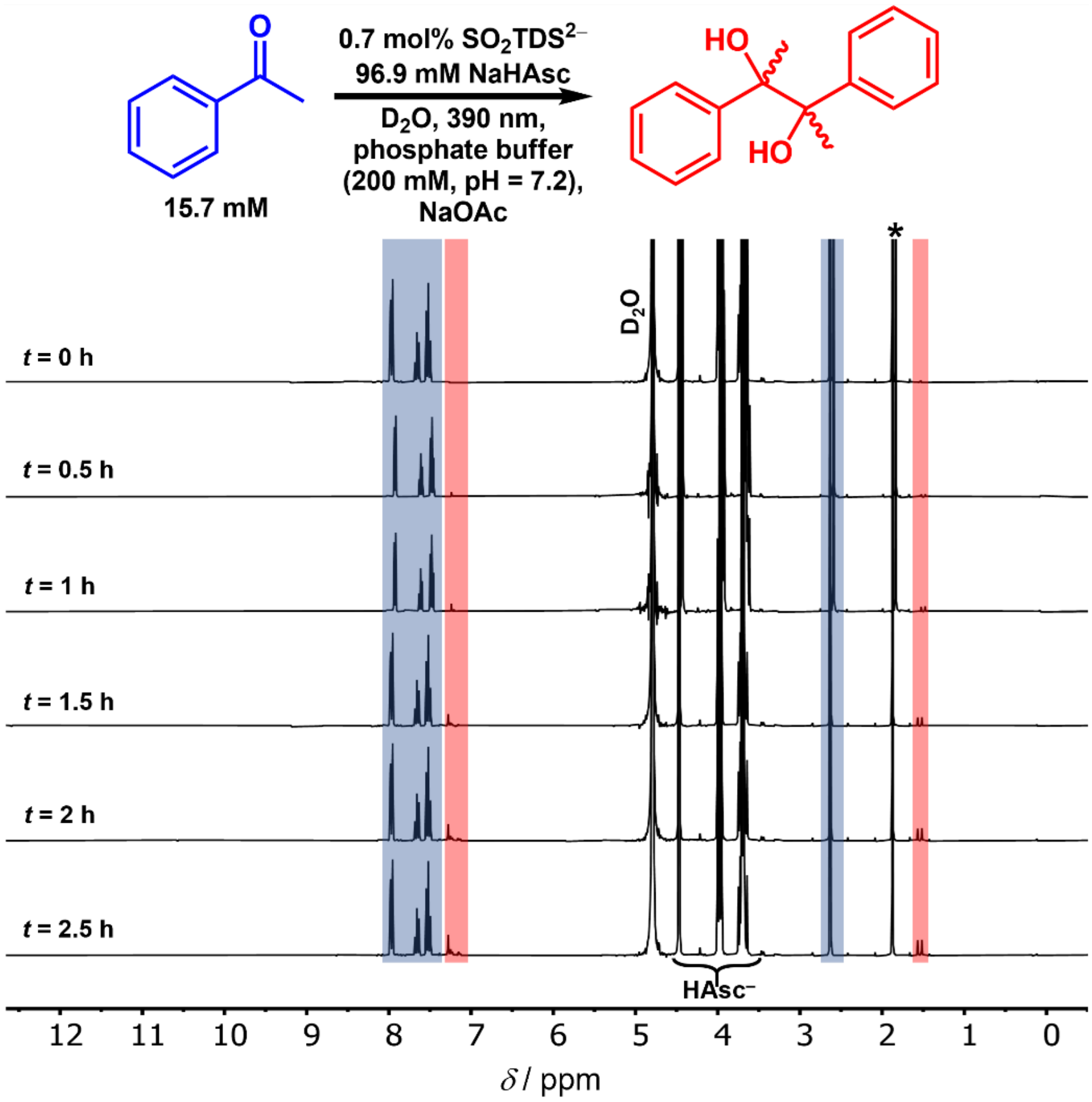

Fig. S41. ^{1}H NMR spectra of the pinacol coupling of acetophenone using SO_2TDS^{2-}, a 390 nm LED, and a higher concentration of NaHAsc (96.9 mM, 6.2 eq.) in Ar-saturated D_2O (200 mM phosphate buffer, pH = 7.2). The product formation was quantified using the CH_3 proton signals (at $\delta \sim 1.5$ ppm, the two signals correspond to the D/L and the meso forms of the product) relative to the internal standard (NaOAc, assigned with an asterisk). See also Fig. S39 for the resulting time-resolved product formation plot.

S4 Assessing the Sensitivity of Kinetic Parameters in Model TADF Photocatalytic Systems

For illustrative purposes, selected kinetic analyses were conducted on hypothetical model systems to assess how variations in S_1 and T_1 lifetimes affect total free radical production. Importantly, the effect of changing $\tau_{0,S}$ in particular, depends strongly on which kinetic rate constant is varied to achieve that change. Thus, the same $\tau_{0,S}$ can correspond to substantially different *isc* efficiency and thus different $\Phi_{r,tot}$ depending on the underlying combination of decay rates. We therefore compare the predicted $\Phi_{r,tot}$ at operationally relevant quencher concentrations (50 and 100 mM) and examine the corresponding concentration-dependent kinetic profiles. The selected cases are intended to illustrate representative and photocatalytically relevant trends rather than provide an exhaustive exploration of the full parameters' kinetic space; nevertheless, they demonstrate that variations in individual rate constants can lead to markedly different kinetic responses even when the resulting excited-state lifetimes are similar.

Table S9. Variation of the photophysical parameters and predicted total free-radical quantum yields across model photocatalysts at catalytically relevant quencher concentrations (50 and 100 mM). *Variation* **0** serves as reference system, with photophysical rate constants and cage-escape efficiencies chosen to reflect physically plausible values for TADF photocatalysts, based on values analogous to those of the TADF systems investigated in this work. *Variations* **1a-c** and **2a** refer to changes in singlet- and triplet- related kinetic constants, respectively, which are highlighted in red.

<table>
<tr><th>Variation</th><th>$\tau_{0,S}$ / ns</th><th>$\tau_{0,T}$ / µs</th><th>k_{fluo}+ k_{ic} / s^{-1}</th><th>k_{isc} / s^{-1}</th><th>k_{risc}+ k_{ph} + $k'_{isc,T}$ / s^{-1}</th><th>$\eta_{ce,S}$</th><th>$\eta_{ce,T}$</th><th>$k_{q,S}$ / $M^{-1}s^{-1}$</th><th>$k_{q,T}$ / $M^{-1}s^{-1}$</th><th>η_{isc} at 0 mM</th><th>$\Phi_{r,tot}$ at 50 mM</th><th>$\Phi_{r,tot}$ at 100 mM</th></tr>
<tr><td>0</td><td>2</td><td rowspan="4">20</td><td>1.7×10^8</td><td>2×10^8</td><td rowspan="4">5×10^4</td><td rowspan="5">0.03</td><td rowspan="5">0.4</td><td rowspan="5">4×10^9</td><td rowspan="5">2×10^9</td><td>0.56</td><td>0.16</td><td>0.13</td></tr>
<tr><td>1a</td><td>5</td><td>1.7×10^8</td><td>2×10^7</td><td>0.10</td><td>0.035</td><td>0.033</td></tr>
<tr><td>1b</td><td>5</td><td>1.1×10^7</td><td>2×10^8</td><td>0.95</td><td>0.21</td><td>0.15</td></tr>
<tr><td>1c</td><td>32</td><td>1.1×10^7</td><td>2×10^7</td><td>0.65</td><td>0.060</td><td>0.065</td></tr>
<tr><td>2a</td><td>2</td><td>70</td><td>1.7×10^8</td><td>2×10^8</td><td>1×10^3</td><td>0.56</td><td>0.16</td><td>0.13</td></tr>
</table>

As shown in Table S9, $\tau_{0,S}$ increases from 2 to 5 ns (*variation* **1a**) by decreasing k_{isc}. In this case, fluorescence and internal conversion outcompete *isc*, resulting in a strongly reduced efficiency of *isc*. In the context of photoredox catalysis, this leads to a lower $\Phi_{r,tot}$ because the triplet population is suppressed in favor of processes leading the system back to S_0. Alternatively, $\tau_{0,S}$ may increase by reducing k_{fluo}+k_{ic} (*variation* **1b**): deactivations to S_0 are highly suppressed. By maintaining a high k_{isc}, intersystem crossing becomes almost the exclusive decay pathway of S_1, leading to an improved population of T_1, which in turn improves the free radicals' production. Elongating $\tau_{0,S}$ further, lowering both k_{fluo}+k_{ic} and k_{isc}

by one order of magnitude (*variation* **1c**) in comparison to *variation* **0**, decreases the $\Phi_{r,tot}$: although being as fast as $k_{fluo}+k_{ic}$, the low k_{isc}, as absolute value leaves S_1 more exposed to the unproductive and comparatively fast bimolecular quenching. The η_{isc} in the absence of a quencher should thus not be considered an absolute predictor of photocatalytic performances (e.g. comparing *variation* **0** and **1c**). It only describes the intrinsic competition of *isc* with fluorescence and internal conversion but does not provide information on the ability of *isc* in competing with quenching processes: the absolute value of k_{isc} should also be considered. An increase in $\tau_{0,T}$ (*variation* **2a**) while maintaining a short $\tau_{0,S}$ does not translate directly into a better performance at common catalytically relevant quencher concentrations, as in those conditions the rate of bimolecular triplet quenching is already several orders of magnitude faster than the rates of unimolecular triplet decays. However, it expands the operational window toward lower quencher regimes, enabling bimolecular quenching even under highly dilute conditions. The corresponding kinetic plots are shown in Figure S42.

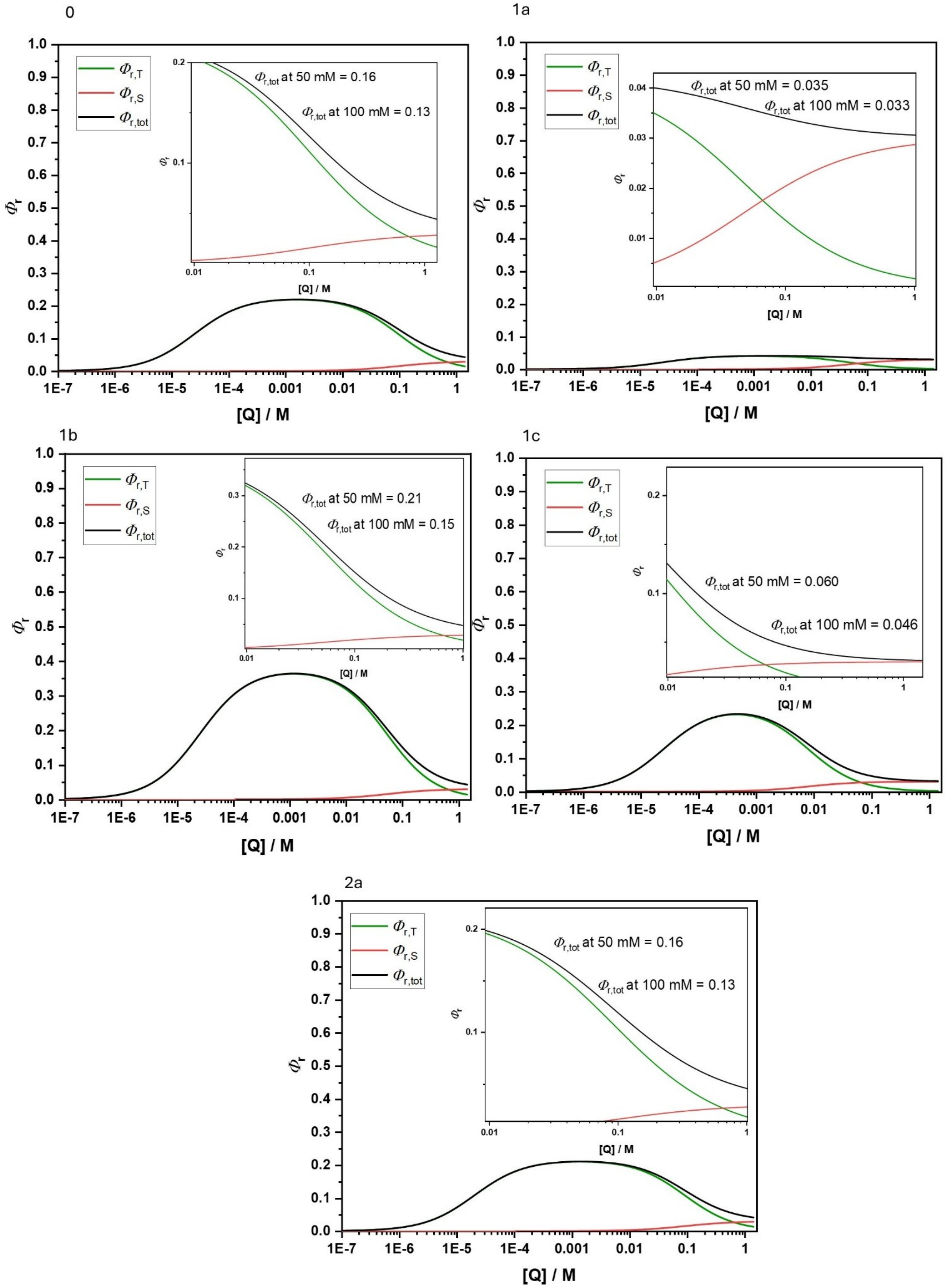

Figure S42. Kinetic plots corresponding to variations **0**,**1a-c** and **2a** reported in Table S9. Insets show the trends at catalytically relevant quencher concentrations.

S5 Synthetic Procedures

Synthesis of **P1**

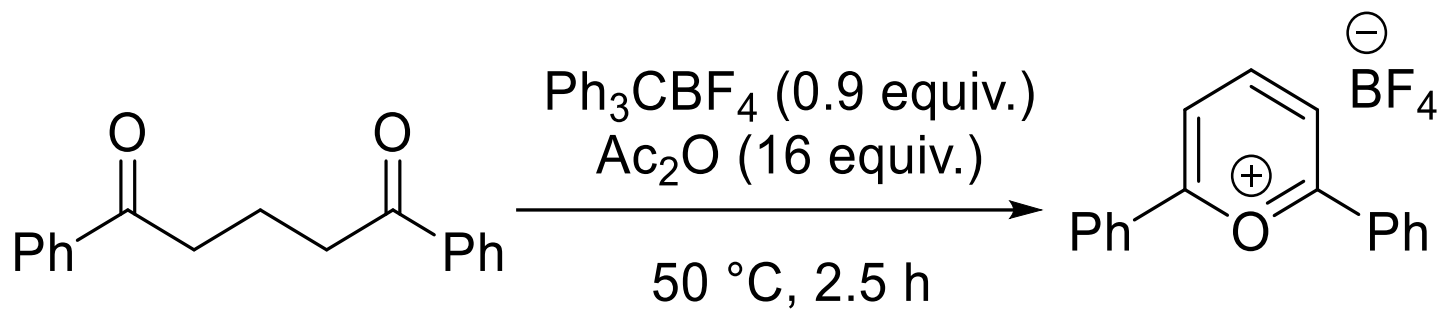

Pyrylium salt **P1:** 1,5-Diphenylpentane-1,5-dione (5 g, 19.82 mmol, 1 equiv.) was dissolved in acetic anhydride (30 mL, 312.12 mmol, 16 equiv.). Then tritylium tetrafluoroborate (5.9 g, 17.84 mmol, 0.9 equiv.) was added to the red solution. The resulting yellow suspension was stirred for 2.5 h at 50 °C. The brown-yellow solution was cooled to rt and diluted with Et_2O (50 mL) resulting in a yellow suspension. The supernatant was filtered off using a filter cannula and the resulting brown-yellow solid was washed with Et_2O (50 mL), THF (50 mL) and again with Et_2O (2 × 50 mL). Drying of the residue under reduced pressure gave pyrylium salt **P1** (5.05 g, 15.8 mmol, 80 %) as an orange-yellow solid. The spectroscopic data are in good agreement with previous reports.[15]

^{1}H NMR (600 MHz, CD_3CN, 298K): δ [ppm] = 8.96 (t, *J* = 8.4 Hz, 1H), 8.52 (d, *J* = 8.4 Hz, 2H), 8.38-8.32 (m, 4H), 7.88-7.85 (m, 2H), 7.78-7.75(m, 4H); **^{13}C NMR** (151 MHz, CD_3CN, 298K): δ [ppm] = 173.3, 158.3, 136.8, 131.2, 129.8, 129.6, 120.5.

Synthesis of **P2**

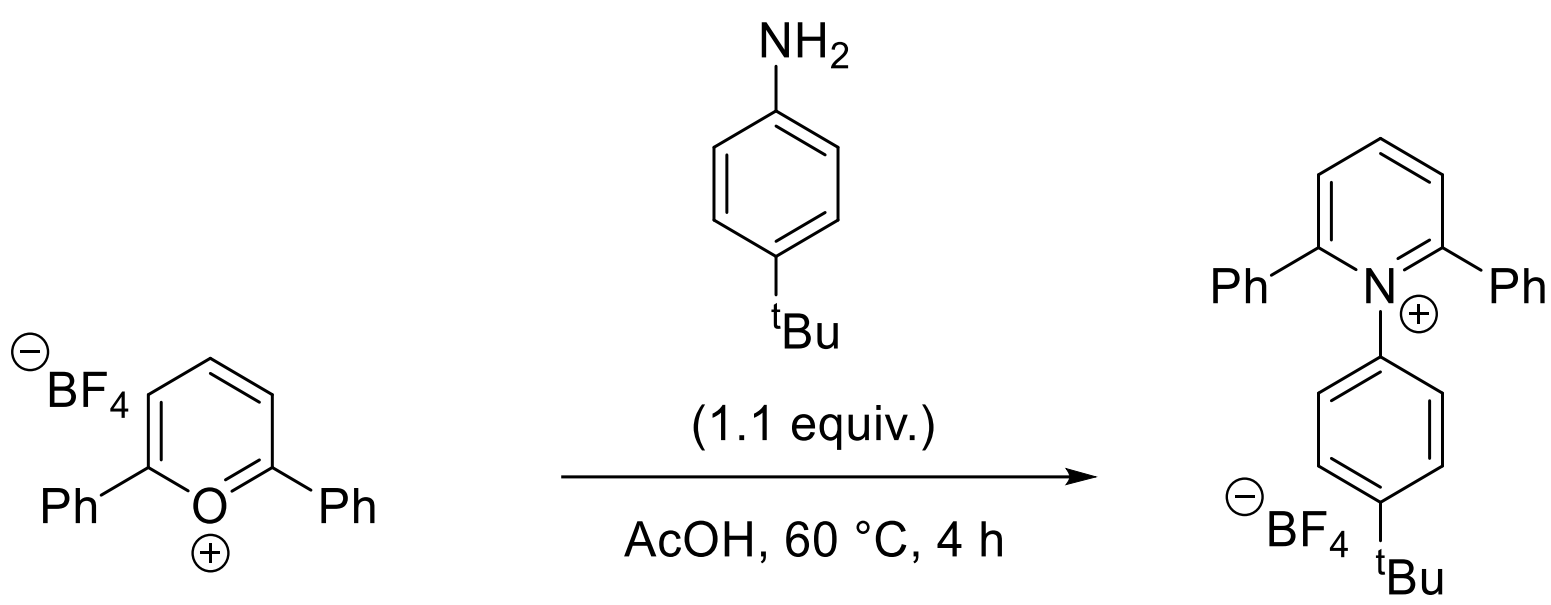

Pyridinium salt **P2:** Pyrylium salt **P1** (2 g, 6.25 mmol, 1 equiv.) was dissolved in glacial acid (40 mL). To this yellow suspension *p-tert*-butyl-aniline (1.03 g, 21.8 mmol, 1.1 equiv.) was added. The now red suspension was stirred for 4 h at 60 °C resulting in a red-brown solution. After cooling down to room temperature the solvent was evaporated. The resulting red solid was dissolved in CH_2Cl_2 (10 mL) and added dropwise to Et_2O (100 mL, strongly stirring). The suspension was filtered and the now orange solid was washed with Et_2O (40 mL). Evaporating of the residue solvent gave pyridinium salt **P2** (1.08 g, 2.4 mmol, 38 %) as a light pink solid. The spectroscopic data are in good agreement with previous reports.[16]

^{1}H NMR (500 MHz, CD_3CN, 298K): δ [ppm] = 8.68 (t, *J* = 8.0 Hz, 1H), 8.11 (d, *J* = 8.0 Hz, 2H), 7.46-7.36 (m, 2H), 7.35-7.26 (m, 8H), 7.20-7.16 (m, 2H), 7.16 – 7.07 (m, 2H), 1.12 (s, 9H); **^{13}C NMR** (126 MHz, CD_3CN, 298K): δ [ppm] = 157.9, 154.8, 147.1, 137.6, 133.8, 131.1, 130.0, 130.2, 129.2, 128.8, 126.6, 35.3, 31.1.

Synthesis of **P3**

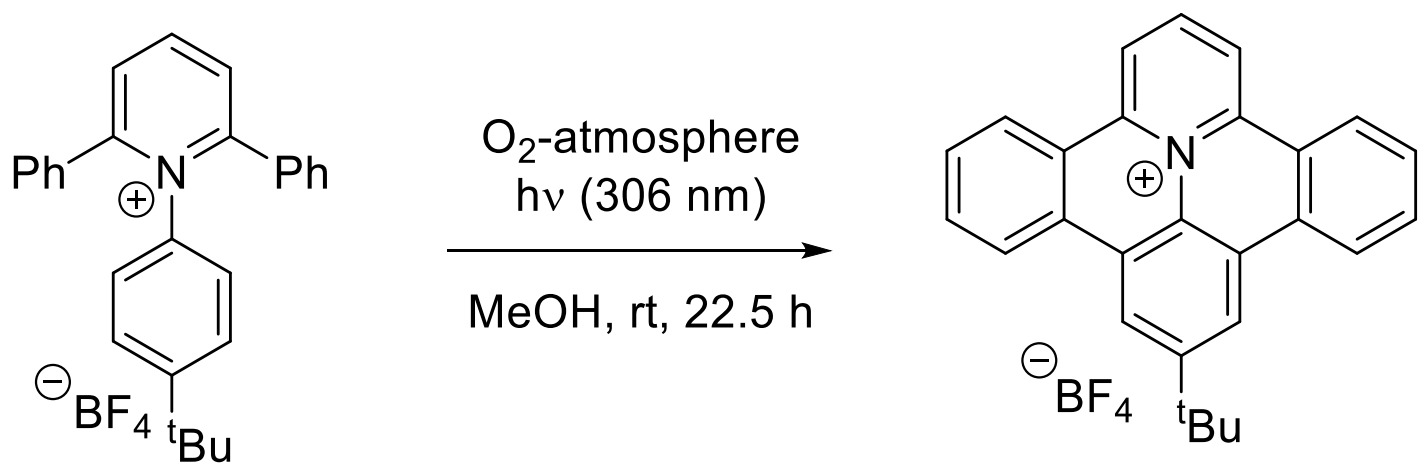

p-tert.-butyl-*aza*-dibenzo-pyrene **P3**: Pyridinium salt **P2** (535 mg, 1.2 mmol, 1 equiv.) was dissolved in methanol (30 mL) and the resulting red solution was irradiated with UV-Light (306 nm) for 22.5 h under oxygen atmosphere. The yellow solid was filtered off and washed with Et_2O until the filtrate was nearly colorless. Drying under reduced pressure afforded *p-tert.*-butyl-*aza*-dibenzo-pyrene **P3** (335.4 mg, 0.749 mmol, 63 %) as a yellow solid.

Modified Procedure:

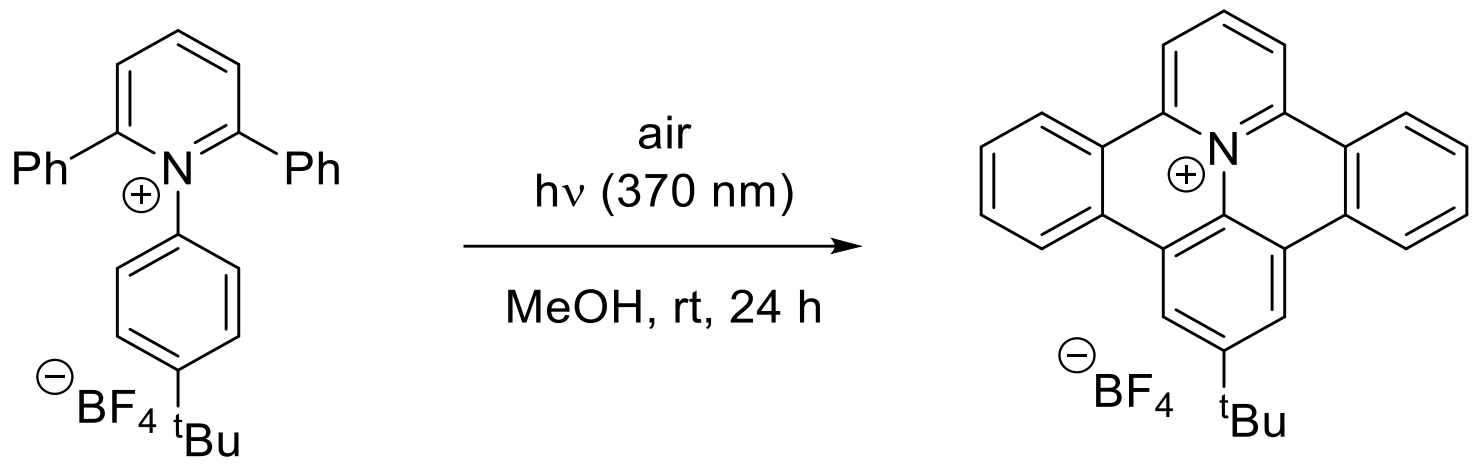

p-tert.-butyl-*aza*-dibenzo-pyrene **P3:** A round bottom flask was charged with pyridinium salt **P2** (1.4 g, 3.1 mmol, 1 equiv.) and methanol (50 mL). The resulting red solution was irradiated with two Kessil lamps (370 nm) for 24h under ambient air. The yellow solid was filtered off and washed with Et_2O until the filtrate was nearly colorless. Drying under reduced pressure afforded *p-tert.*-butyl-*aza*-dibenzo-pyrene **P3** (865.0 mg, 1.93 mmol, 62 %) as a yellow solid. The spectroscopic data are in good agreement with previous reports.[4]

m.p. 364 °C (decomp.); **^{1}H NMR** (500 MHz, CD_3CN): δ [ppm] = 9.15 (d, *J* = 8.2 Hz, 2H, Ar-CH), 8.9 (s, 2H, Ar-CH), 8.77 (d, *J* = 8.2 Hz, 2H, Ar-CH), 8.68 (d, *J* = 8.4 Hz, 2H, Ar-CH), 8.64 (t, *J* = 8.3 Hz, 1H, Ar-CH), 8.01 (m, 2H, Ar-CH), 7.87 (m, 2H, Ar-CH), 1.65 (s, 9H, $C(CH_3)_3$); **^{13}C NMR** (126 MHz, CD_3CN): δ [ppm] = 154.5 (C_q), 143.1 (C_q), 139.7 (CH), 135.0 (CH), 131.7 (CH), 129.7 (C_q), 127.3 (CH), 127.0 (C_q), 125.6 (C_q), 124.6 (C_q), 124.5 (CH), 122.3 (CH), 122.0 (CH), 36.6 ($\mathit{C}(CH_3)_3$), 31.4 ($C(\mathit{C}H_3)_3$); **IR** [cm^{-1}]: ṽ = 3139, 2957, 2916, 2164, 1984, 1627, 1608, 1546, 1499, 1481, 1458, 1418, 1401, 1391, 1373, 1350, 1286, 1254, 1238, 1146, 1094, 1050, 881, 801, 778, 726, 707, 662; **HR-MS-ESI(+)** calc. $[M]^+$ $C_{27}H_{22}N^+$ 360.1747; found: 360.1753; **UV-vis** (CH_3CN)**:** λ_{max}: 328 nm (19400 M^{-1} cm^{-1}), 402 nm (9530 M^{-1} cm^{-1}), 423 nm (12300 M^{-1} cm^{-1}).

Synthesis of **P4**

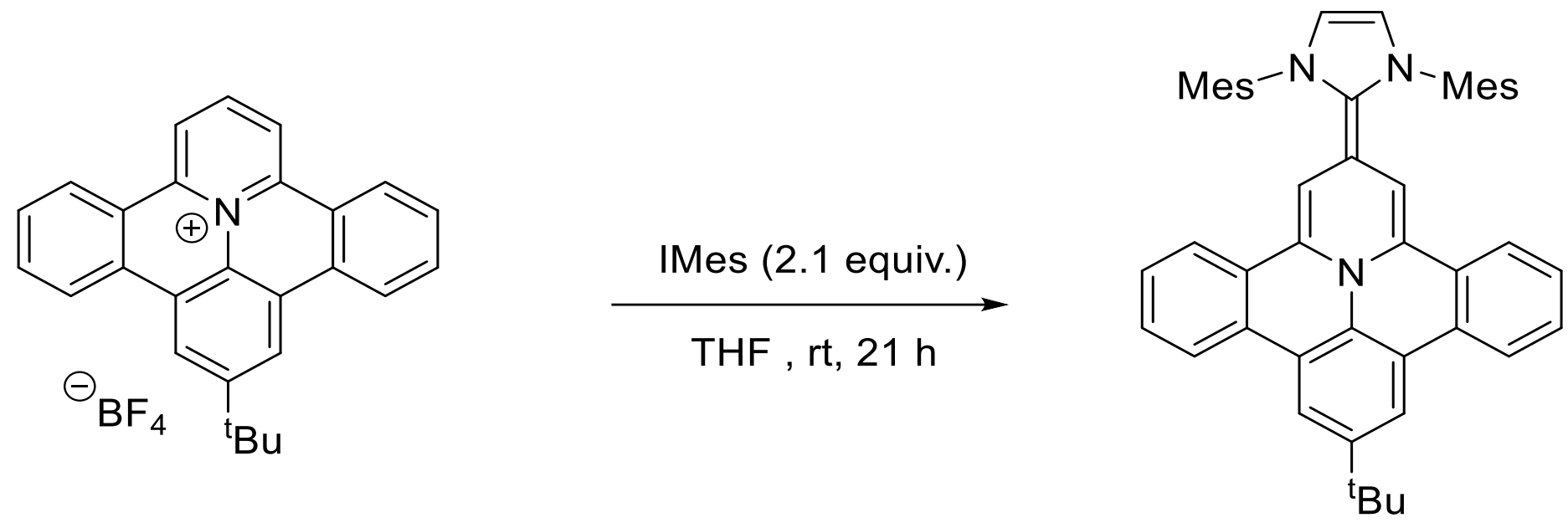

Hybrid system **P4:** *p-tert*-butyl-*aza*-dibenzo-pyrene **P3** (280 mg, 0.626 mmol, 1 equiv.) was dissolved in THF (10 mL) and the resulting yellow suspension was slowly added to a solution of **IMes** carbene (400.21 mg, 1.31 mmol, 2.1 equiv.) in THF (5 mL). The solution turned to a purple color and was stirred for 21 h. The solvent was evaporated under reduced pressure. The dark purple solid was transferred inside a glovebox, dissolved in Et_2O and then filtrated over a celite-plug. Then the solvent was evaporated under reduced pressure. For further purification, a saturated solution of the purple solid in Et_2O was generated and stored at – 40 °C to form a purple crystalline solid. Drying of this solid yielded hybrid-system **P4** (276.2 mg, 0.416 mmol, 67 %) as purple crystals. The spectroscopic data are in good agreement with previous reports.[4]

m.p. 284 °C; **^{1}H NMR** (600 MHz, C_6D_6, 298 K): δ [ppm] = 7.03 (dd, J = 7.3, 2.0 Hz, 2H, Ar-CH), 6.9 (s, 2H, Ar-CH), 6.72 (s, 4H, Mes-CH), 6.69 (td, J = 6.6, 1.7 Hz, 4H, Ar-CH), 5.98 (dd, J = 7.2, 2.0 Hz, 2H, Ar-CH), 5.53 (s, 2H, Ar-CH), 4.50 (s, 2H, NC*H*C*H*N), 2.29 (s, 12H, Mes-^{ortho}CH_3), 2.14 (s, 6H, Mes-^{para}CH_3), 1.11 (s, 9H, C(CH_3)$_3$); **^{13}C NMR** (151 MHz, C_6D_6, 298K): δ [ppm] = 141.3 (C_q), 139.1 (C_q), 137.8 (C_q), 136.8 (C_q), 136.4 (C_q), 132.5 (C_q), 131.6 (C_q), 130.6 (C_q), 130.1 (C_q), 129.1 (Mes-*C*H), 128.4 (Ar-CH), 126.9 (Ar-CH), 121.7 (Ar-CH), 121.6 (Ar-CH), 120.3 (C_q), 120.2 (Ar-CH), 116.0 (Ar-CH), 107.5 (N*C*H*C*HN), 88.7 (C_q), 33.8 (*C*(CH_3)$_3$, 31.1 (C(*C*H_3)$_3$), 21.0 (Mes-^{para}*C*H_3), 18.3 (Mes-^{ortho}*C*H_3); **IR** [cm^{-1}]: ṽ = 3130, 2970, 2948, 2912, 2864, 1658, 1595, 1576, 1561, 1548, 1478, 1424, 1398, 1378, 1348, 1305, 1278, 1239, 1199, 1159, 1110, 1075, 1045, 1033, 1011, 985, 962, 930, 912, 887, 857, 801, 768, 744, 625, 574; **HR-MS-ESI(+)** calc. $[M+H]^+$ $C_{48}H_{46}N_3^+$ 664.3686; found: 664.3673; **UV-vis** (THF)**:** λ_{max}: 300 nm (26900 M^{-1} cm^{-1}), 399 nm (20650 M^{-1} cm^{-1}), 519 nm (14300 M^{-1} cm^{-1}); **CCDC**: 2467868.

Synthesis of **PC^{2+}**

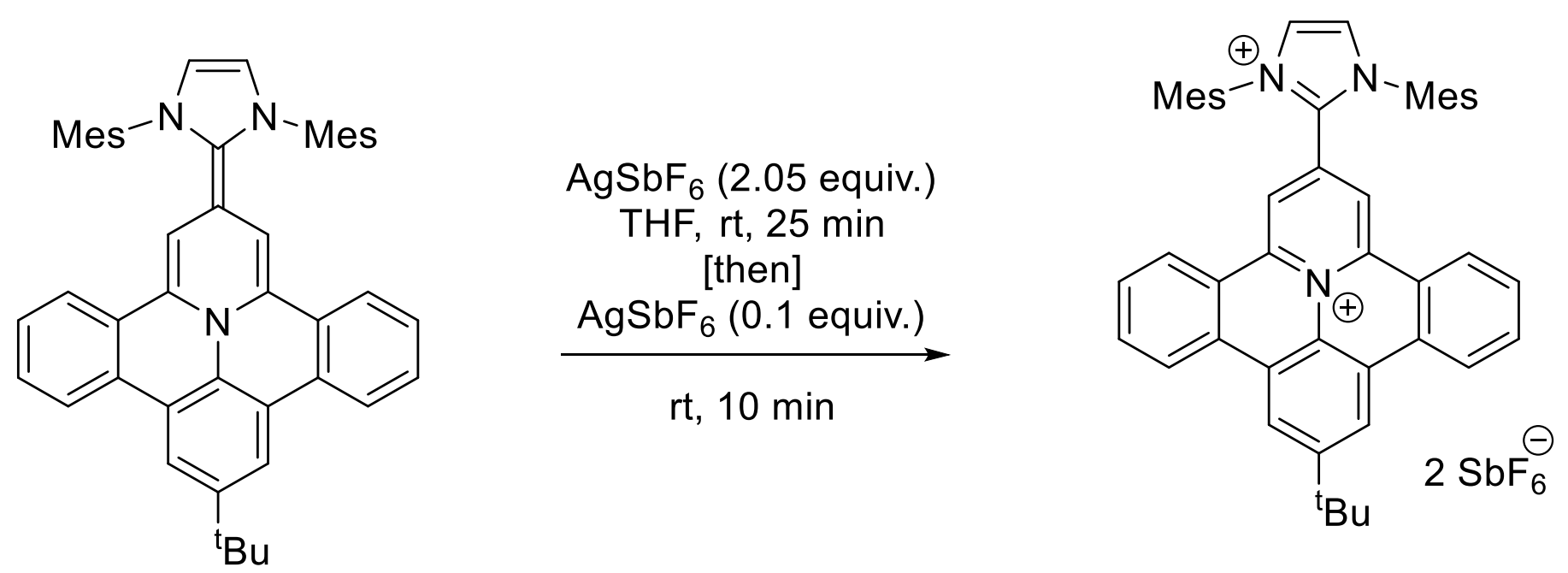

Dicationic photocatalyst **PC^{2+}** (used as SbF_6-salt throughout this study): $AgSbF_6$ (51.03 mg, 0.149 mmol, 2.05 equiv.) was dissolved in THF (2 mL) and added dropwise to a solution of

hybrid-system **P4** (48.1 mg, 0.072 mmol, 1 equiv.) in THF (5 mL). The yellow-green solution was stirred for 25 minutes. $AgSbF_6$ (2.5 mg, 0.007 mmol, 0.1 equiv.) was added again and the now yellow solution was then stirred for an additional 10 minutes. The solution was filtered over a celite plug and extracted with THF and CH_3CN. The solvent was evaporated until only 1 mL was left. Et_2O (5 mL) was added to form a yellow precipitate. After two minutes the Et_2O was decanted off and the resulting yellow solid was washed with Et_2O (2 × 5 mL). The yellow solid was dried to give **PC^{2+}** (46.8 mg, 0.0412 mmol, 57 %) as a yellow solid. The spectroscopic data are in good agreement with previous reports.[4]

m.p. 353 °C (decomp.); **^{1}H NMR** (400 MHz, CD_3CN): δ [ppm] = 9.12 (s, 2H, Ar-CH), 9.97 (d, *J* = 7.8 Hz, 2 H, Ar-CH), 8.48 (s, 2H, Ar-CH), 8.25 (s, 2H, Ar-CH), 8.15 (m, 2H, Ar-CH), 7.95 (m, 2H, Ar-CH), 7.69 (d, *J* = 8.1 Hz, 2H, Ar-CH), 7.34 (s, 4H, Mes-CH), 2.38 (s, 6H, Mes-^{para}CH$_3$) 2.23 (s, 12H, Mes-^{ortho}CH$_3$), 1.65 (s, 9H, C(C*H*$_3$)$_3$; **^{13}C NMR** (101 MHz, CD_3CN): δ [ppm] = 156.1 (C_q), 144.1 (C_q), 144.0 (C_q), 138.7 (C_q), 136.1 (Ar-*C*H), 135.9 (C_q), 132.0 (Ar-*C*H), 131.9 (Mes-*C*H), 131.22 (C_q), 130.8 (C_q), 128.8 (Ar-*C*H), 128.7 (C_q), 127.0 (Ar-*C*H), 126.1 (C_q), 125.3 (Ar-*C*H), 124.2(C_q), 122.9 (Ar-*C*H), 120.0 (Ar-*C*H), 37.0 (*C*(CH$_3$)$_3$), 31.4 (C(*C*H$_3$)$_3$), 21.2 (Mes-*C*H$_3$), 18.2 (Mes-*C*H$_3$); **IR** [cm^{-1}]: $\tilde{\nu}$ = 3172, 2976, 1990, 1635, 1608, 1560, 1478, 1459, 1406, 1385, 1343, 1289, 1230, 1141, 1110, 1034, 984, 932, 854, 769, 731, 721, 708, 652, 576, 500, 440, 429; **HR-MS-ESI(+)** calc. [M]$^{2+}$ $C_{48}H_{45}N_3^{2+}$ 331.6801; found: 331.6790; **UV-vis** (CH_3CN)**:** λ_{max}: 309 nm (132470 M^{-1} cm^{-1}), 361 nm (48700 M^{-1} cm^{-1}), 477 nm (15600 M^{-1} cm^{-1}).

NMR Spectra

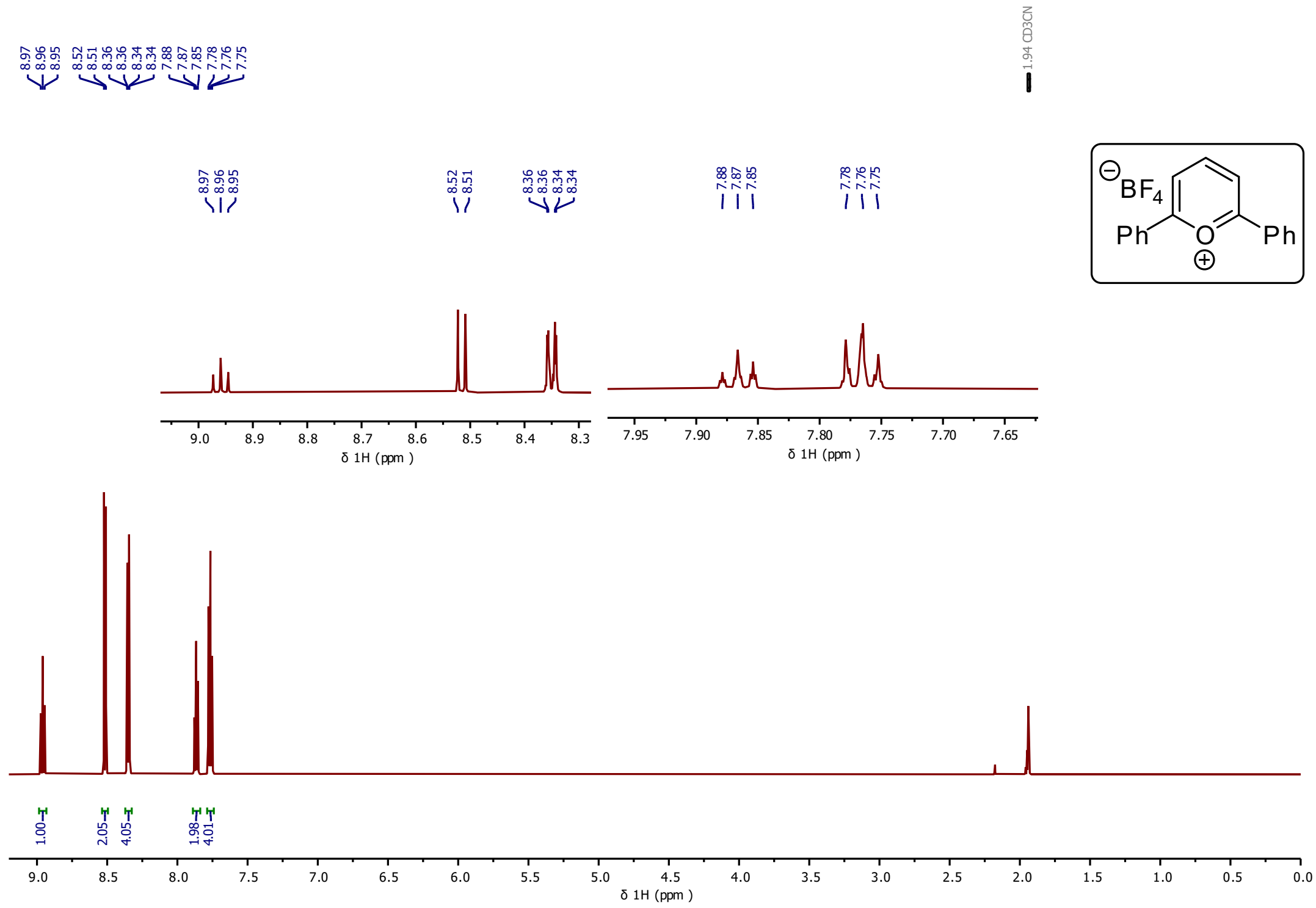

Fig. S43. ^{1}H NMR (600 MHz, CD_3CN, 298K) of **P1**.

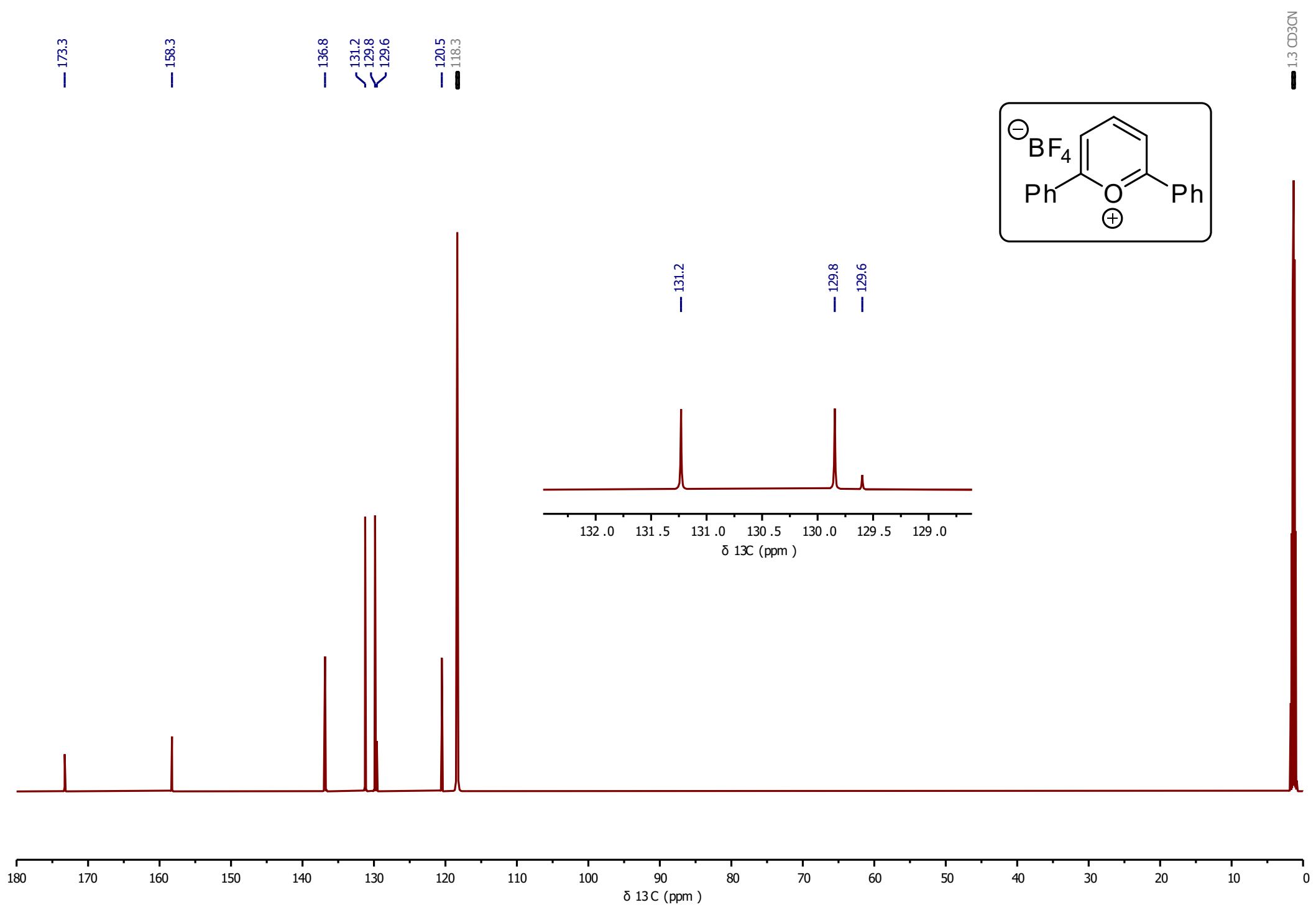

Fig. S44. $^{13}C\{^{1}H\}$ NMR (151 MHz, CD_3CN, 298K) of **P1**.

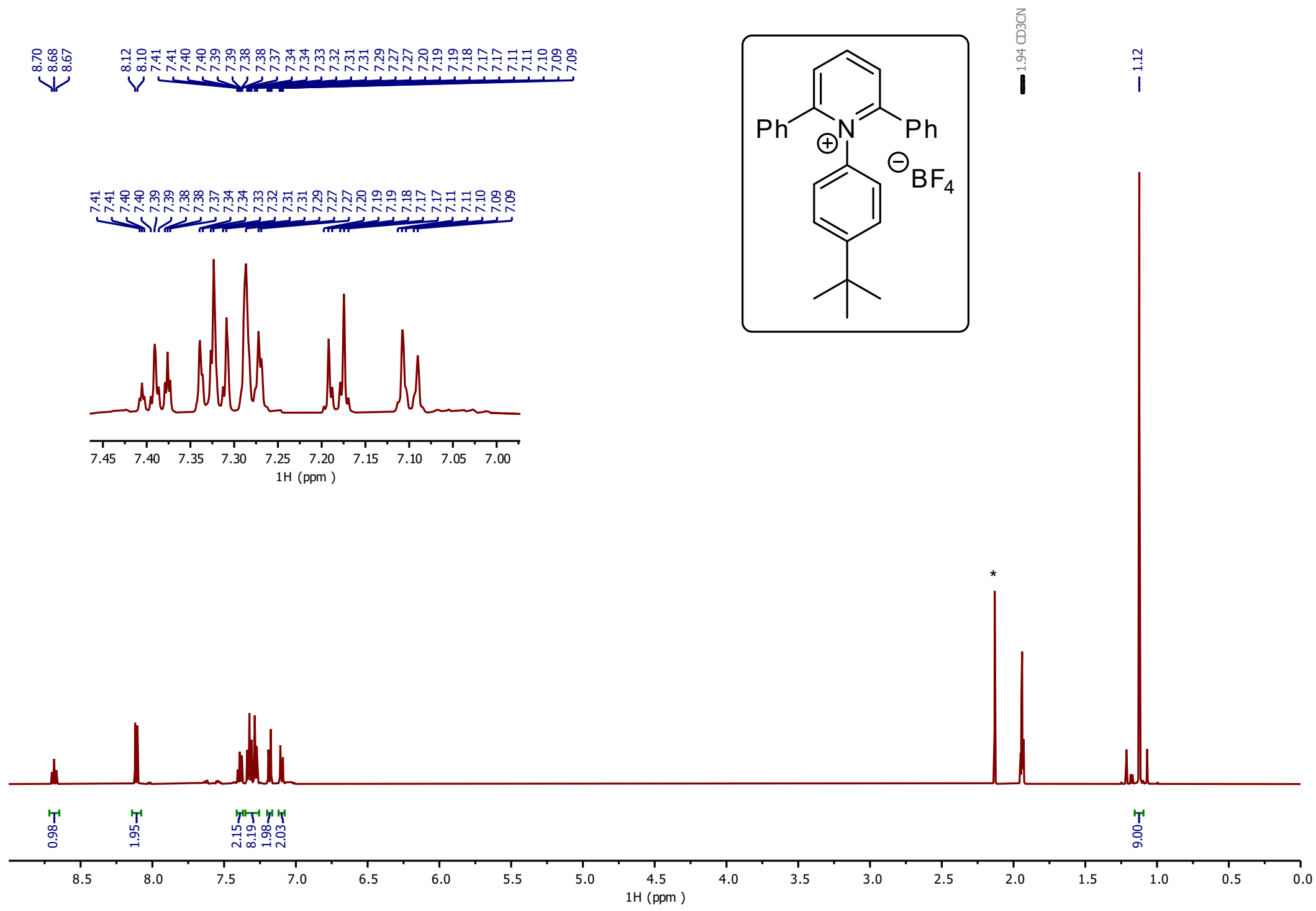

Fig. S45. ^{1}H NMR (500 MHz, CD_3CN, 298K) of **P2** (The water signal is marked with a *).

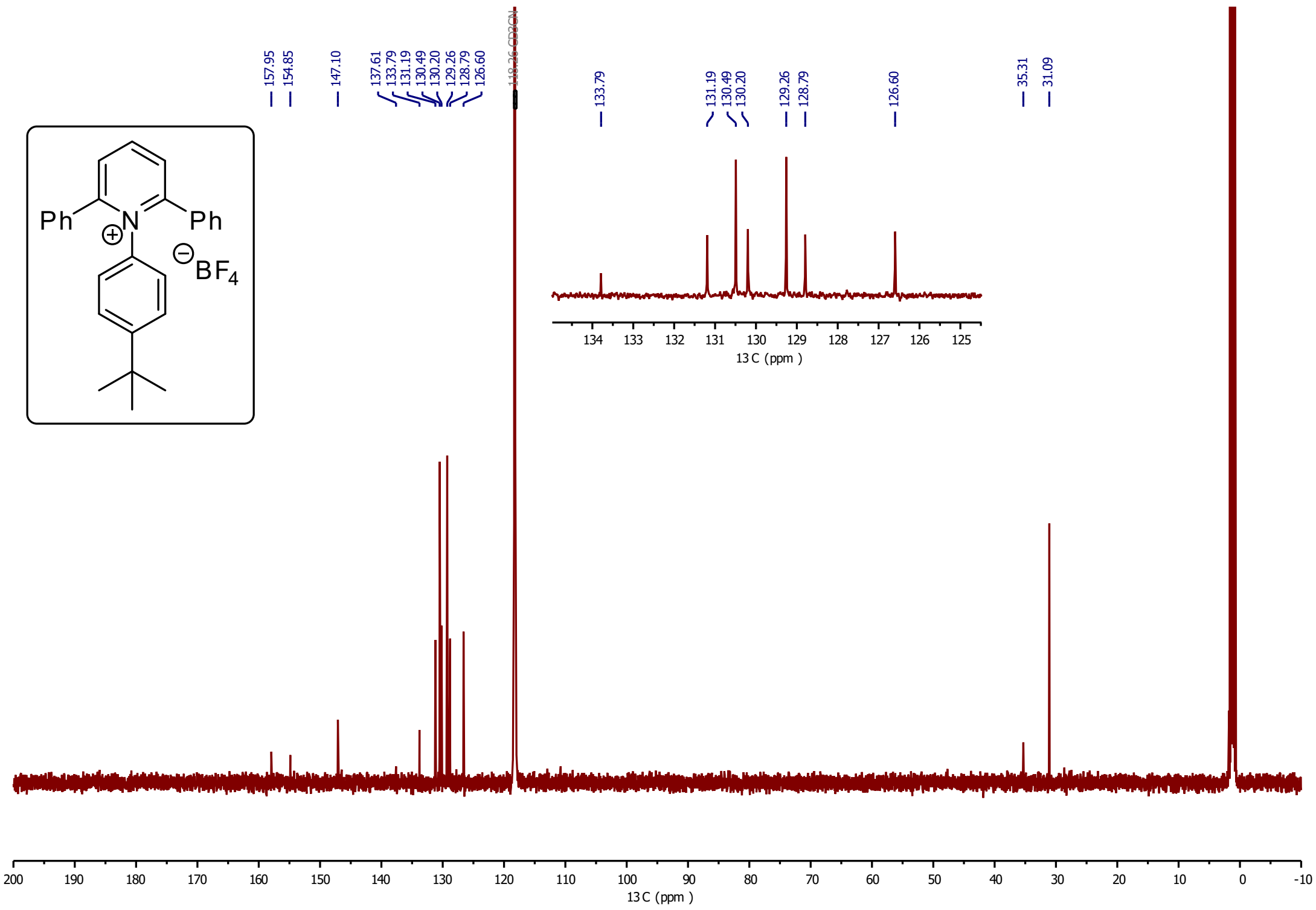

Fig. S46. $^{13}C\ \{^{1}H\}$ NMR (126 MHz, CD_3CN, 298K) of **P2**.

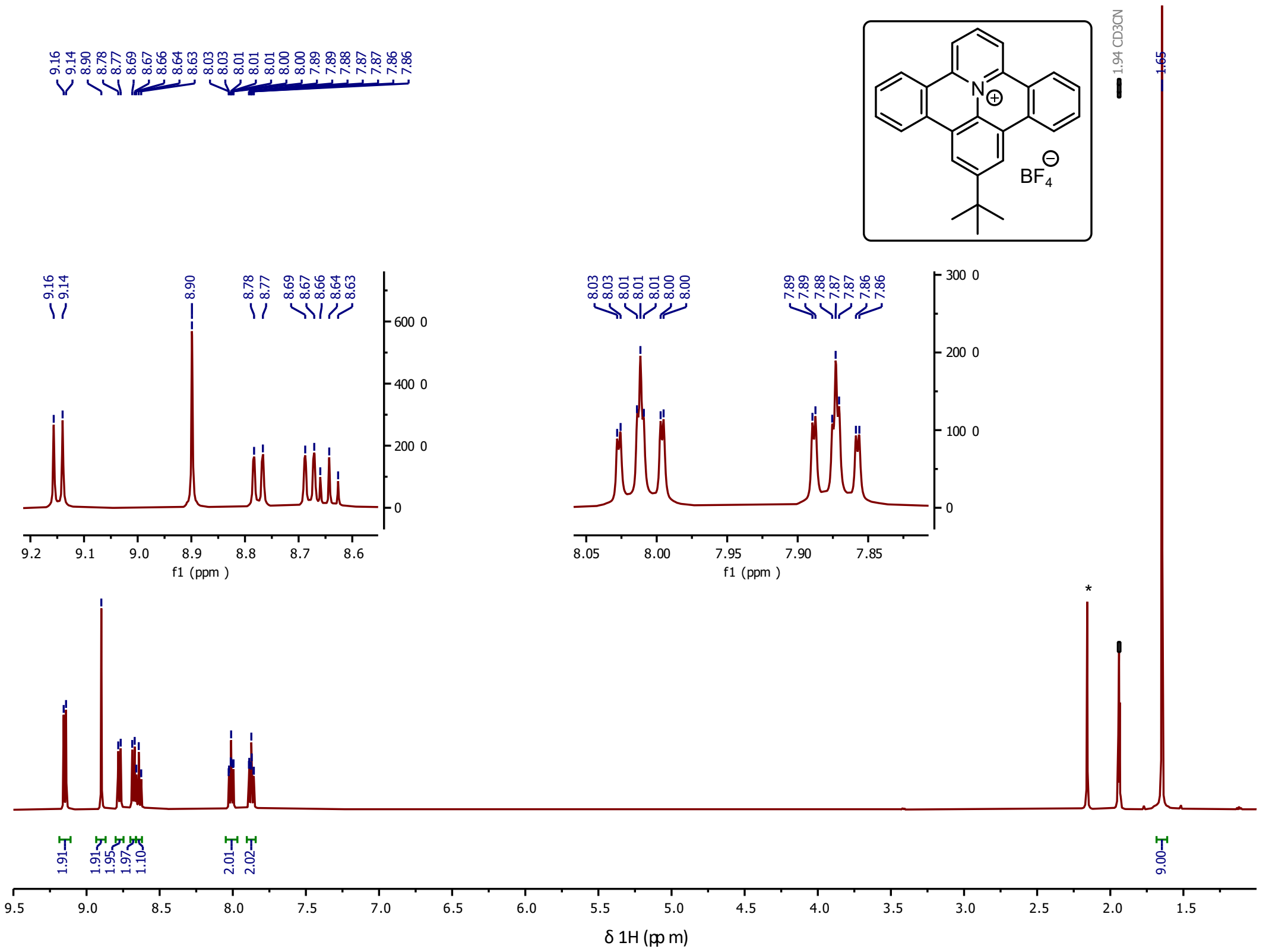

Fig. S47. ^{1}H NMR (500 MHz, CD_3CN, 298K) of **P3** (The water signal is marked with a *).

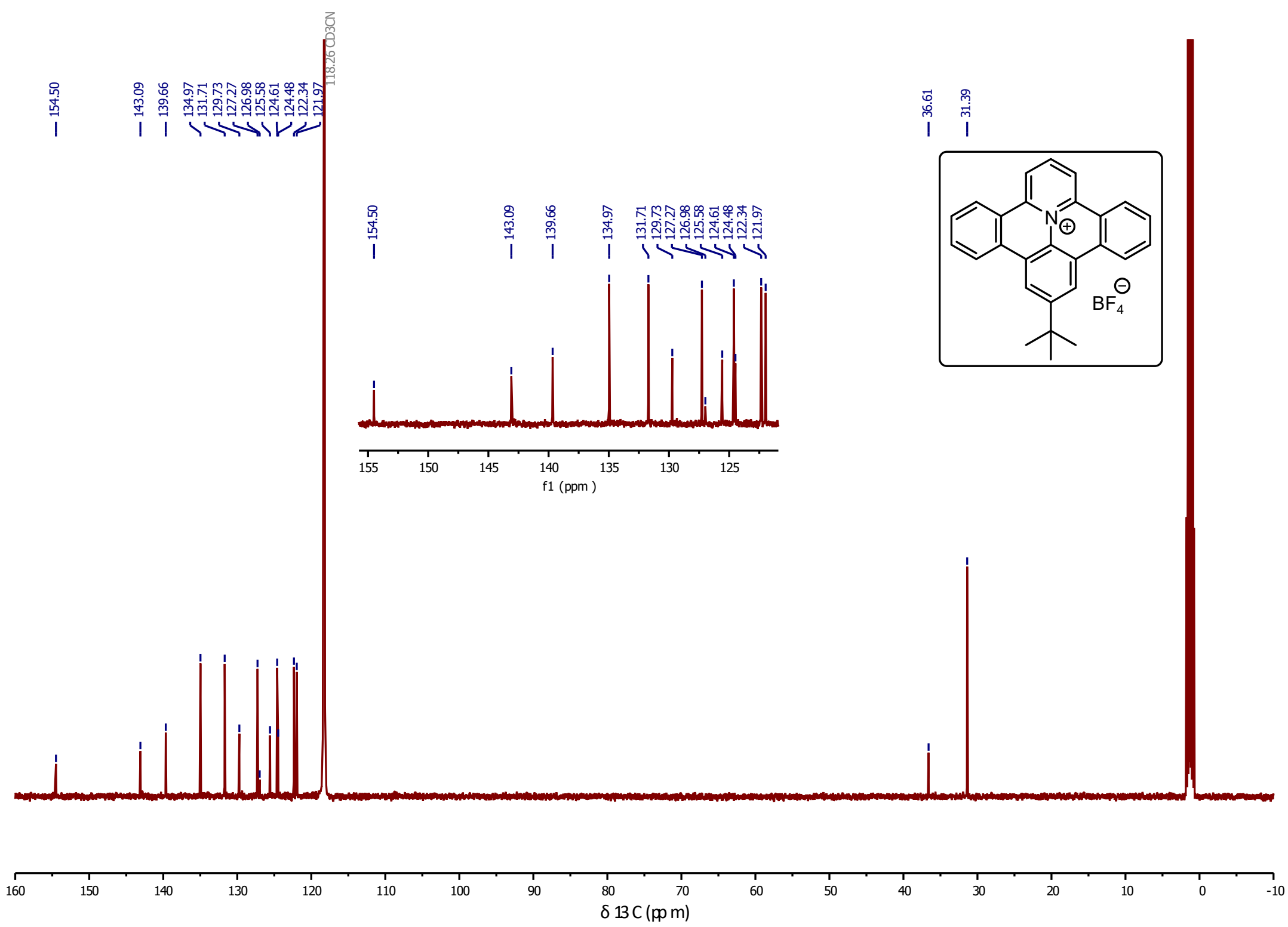

Fig. S48. $^{13}C\ \{^{1}H\}$ NMR (126 MHz, CD_3CN, 298 K) of **P3**.

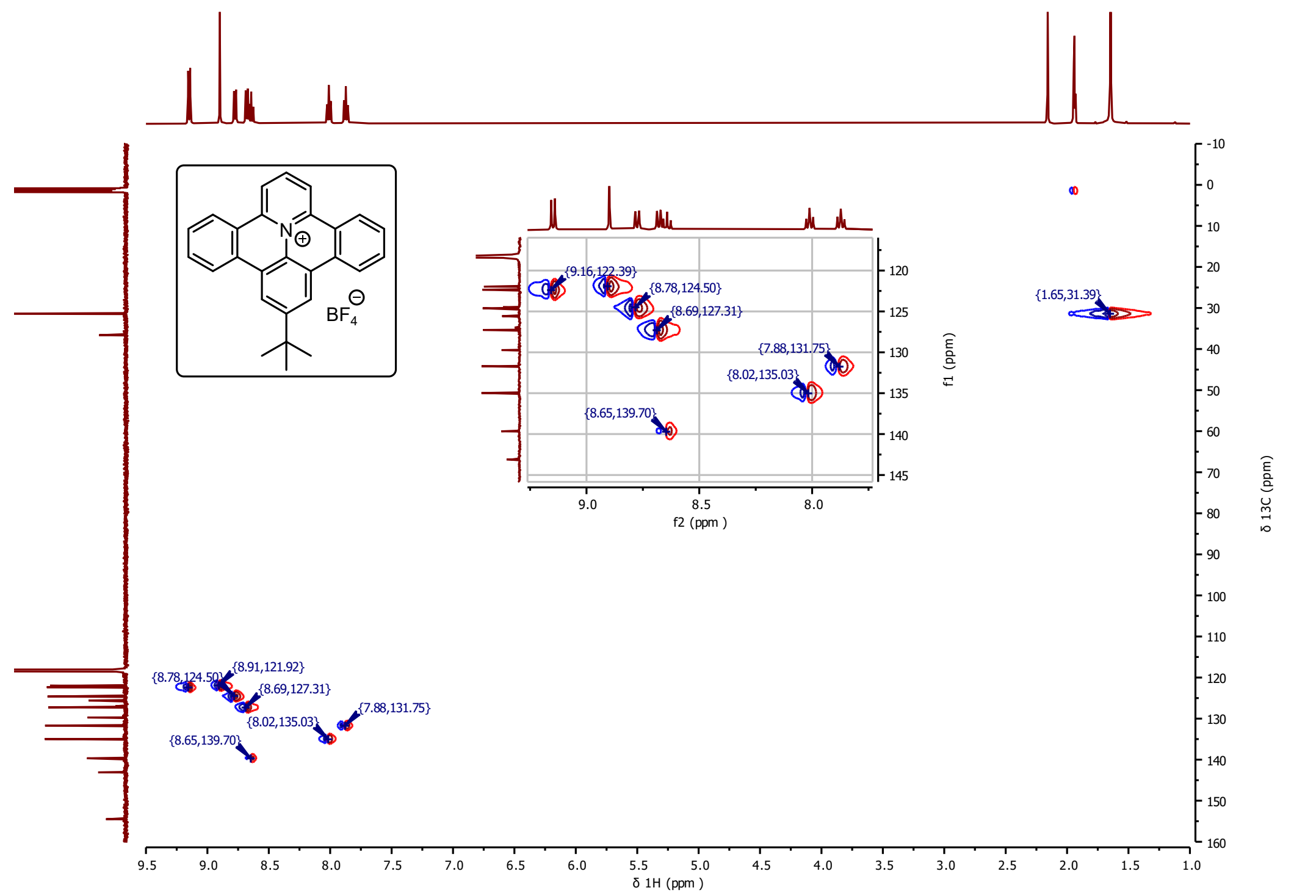

Fig. S49. $^{1}H/^{13}C$ HSQC (500/126 MHz, CD_3CN) of **P3**.

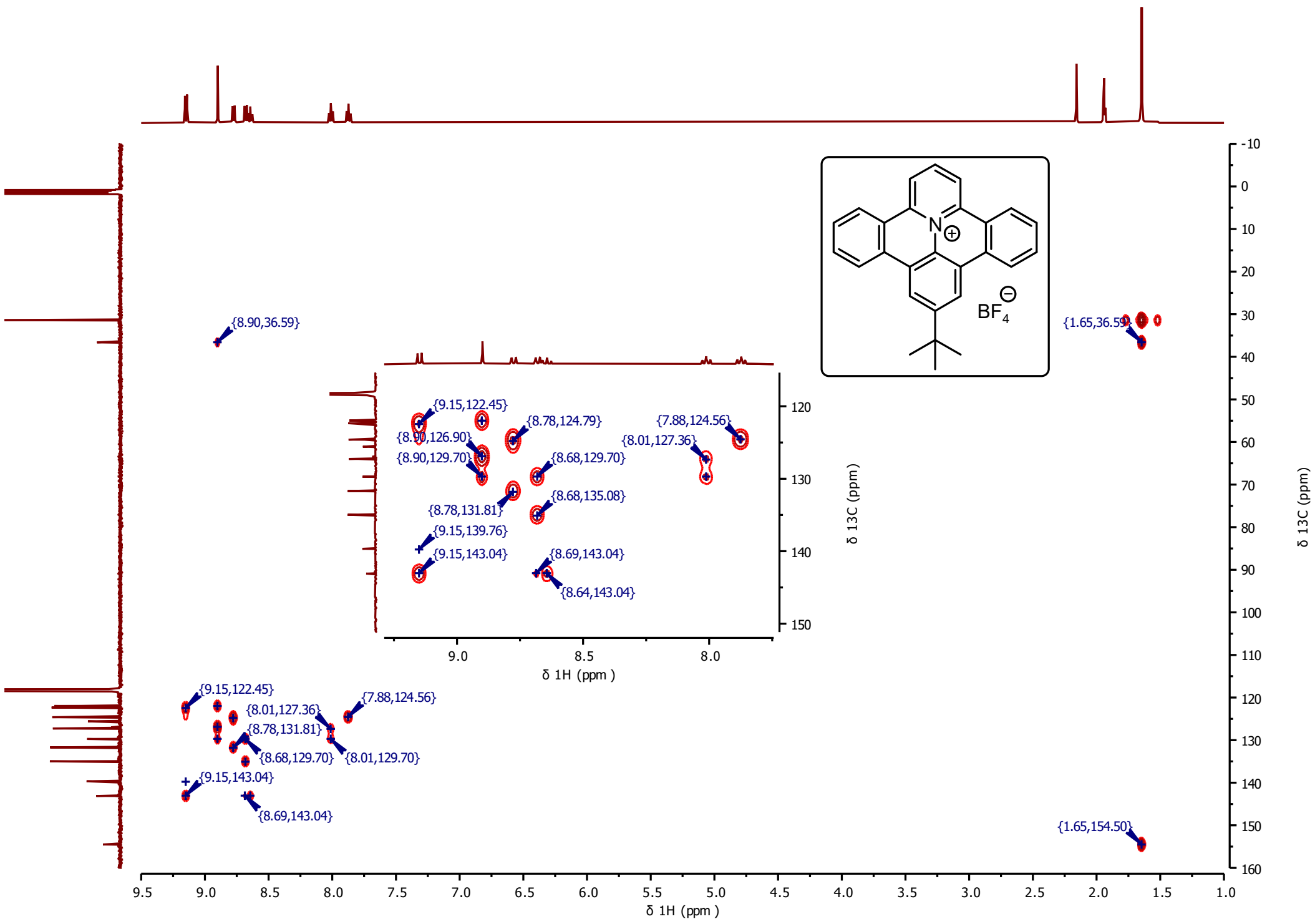

Fig. S50. $^{1}H/^{13}C$ HMBC (500/126 MHz, CD_3CN) of **P3**.

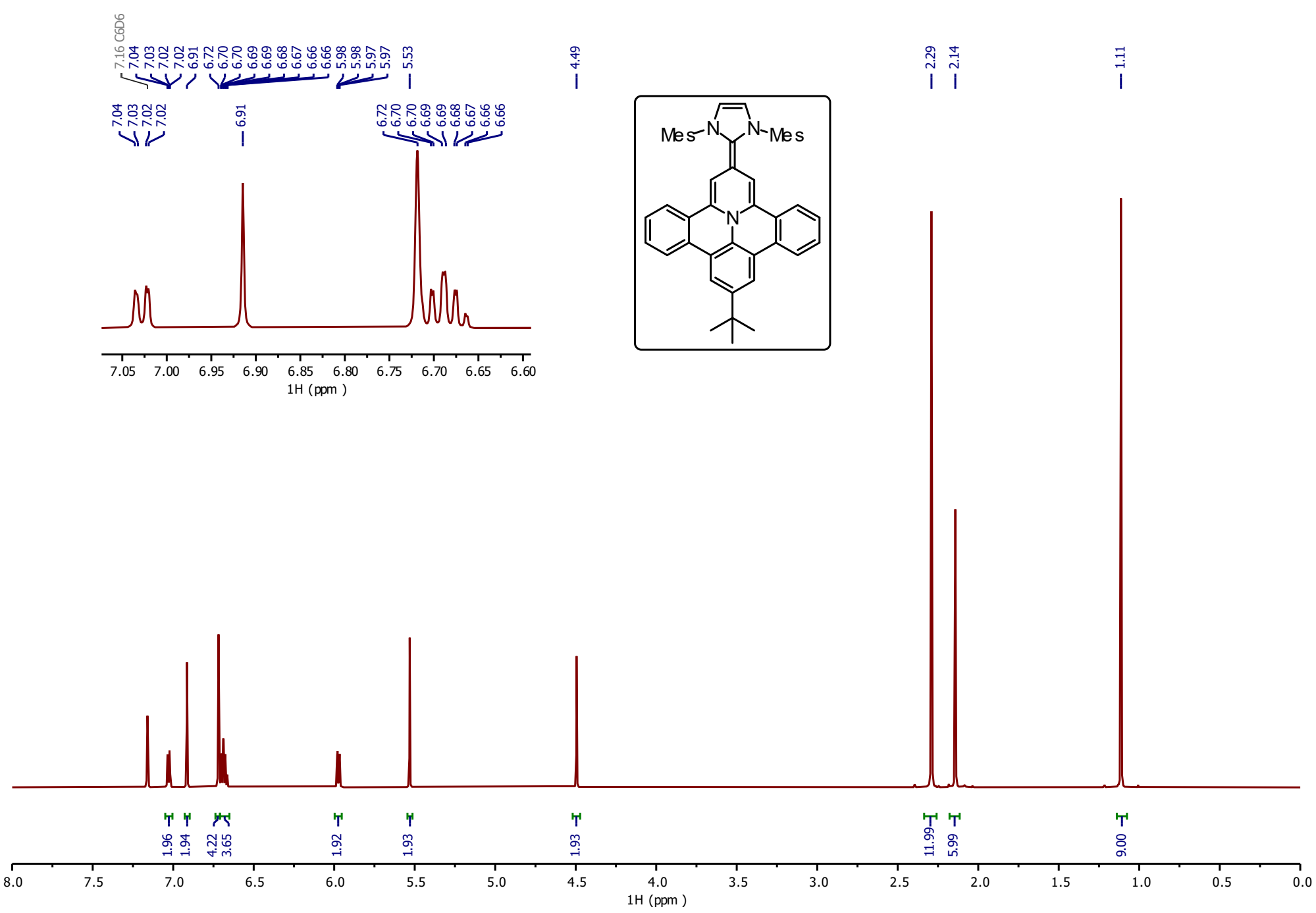

Fig. S51. ^{1}H NMR (600 MHz, C_6D_6, 298K) of **P4**.

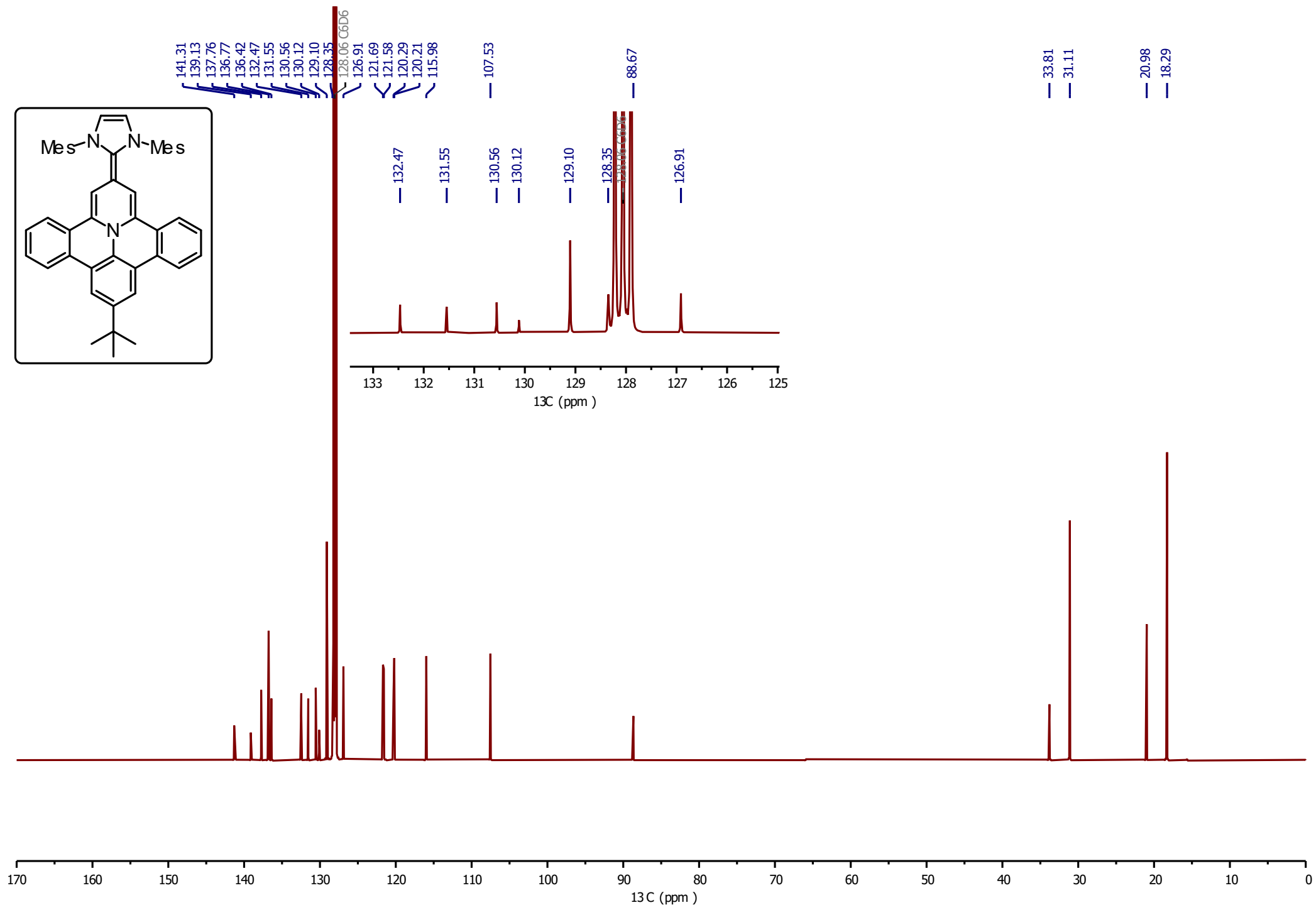

Fig. S52. $^{13}C\ \{^1H\}$ NMR (151 MHz, C_6D_6, 298K) of **P4**.

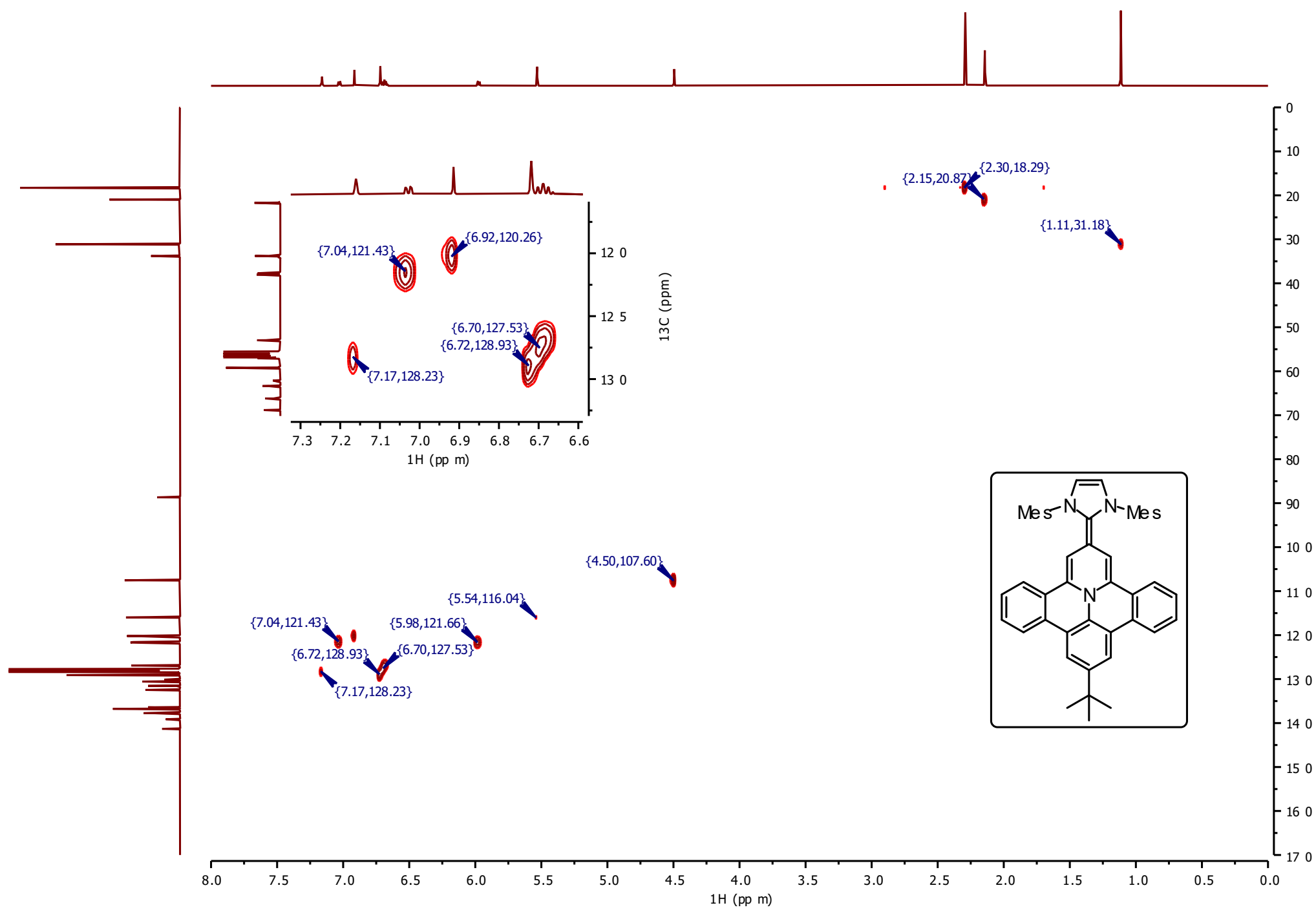

Fig. S53. $^1H/^{13}C$ HSQC (600/151 MHz, C_6D_6, 298K) of **P4**.

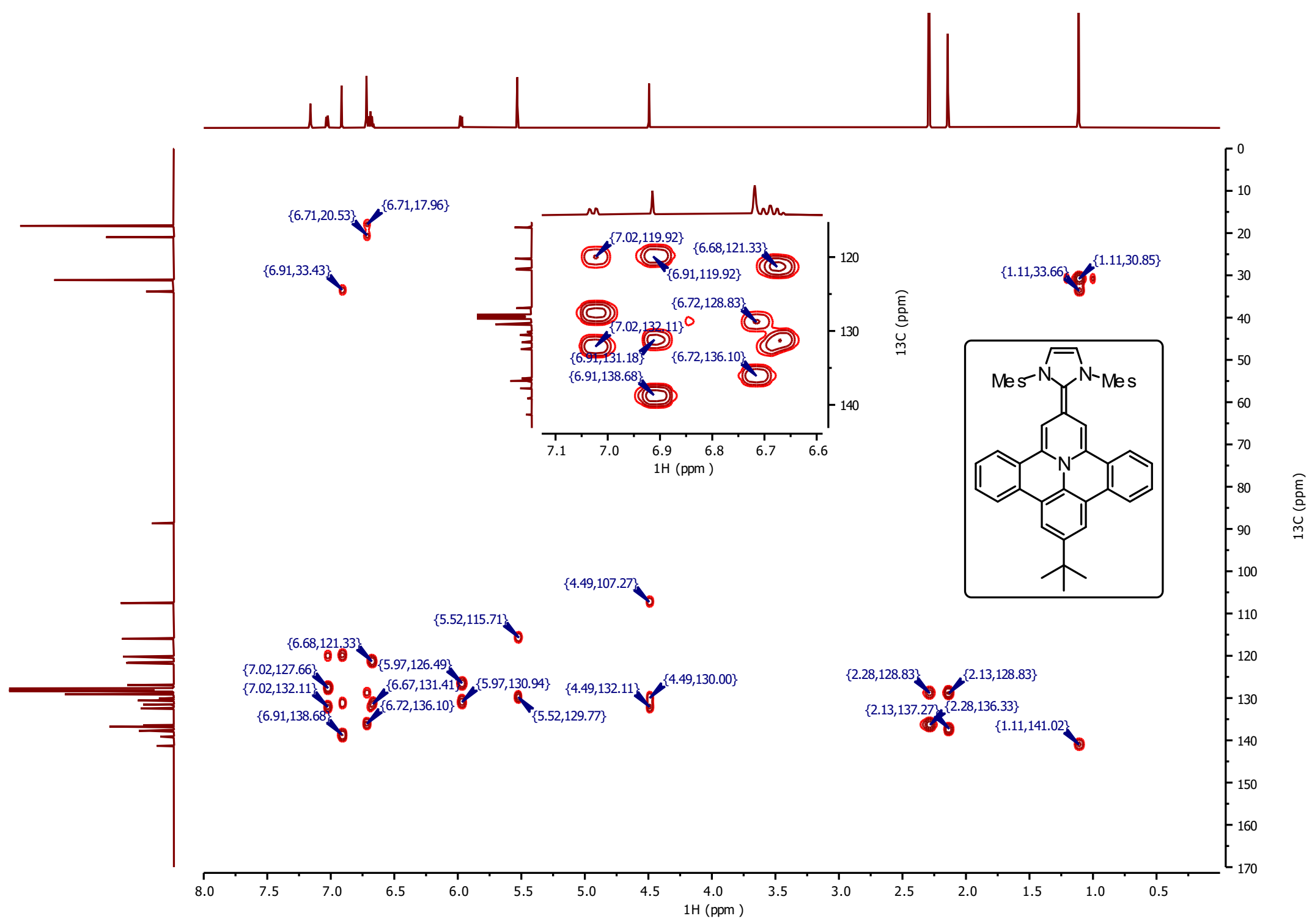

Fig. S54. ^{1}H/^{13}C HMBC (600/151 MHz, C_6D_6, 298K) of **P4**.

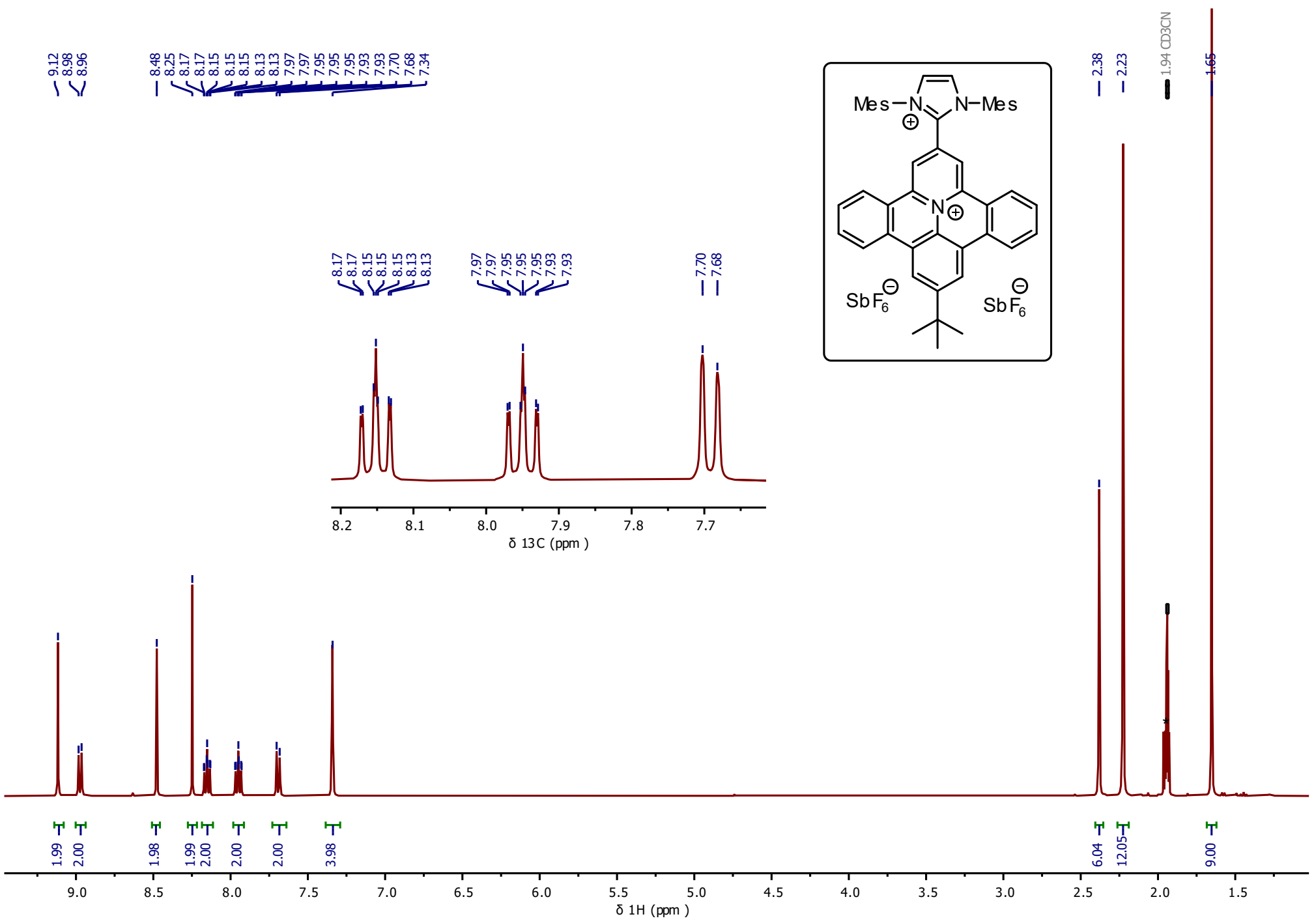

Fig. S55. ^{1}H NMR (400MHz, CD_3CN, 298K) of $\mathbf{PC^{2+}}$ (residual CH_3CN is marked with a *).

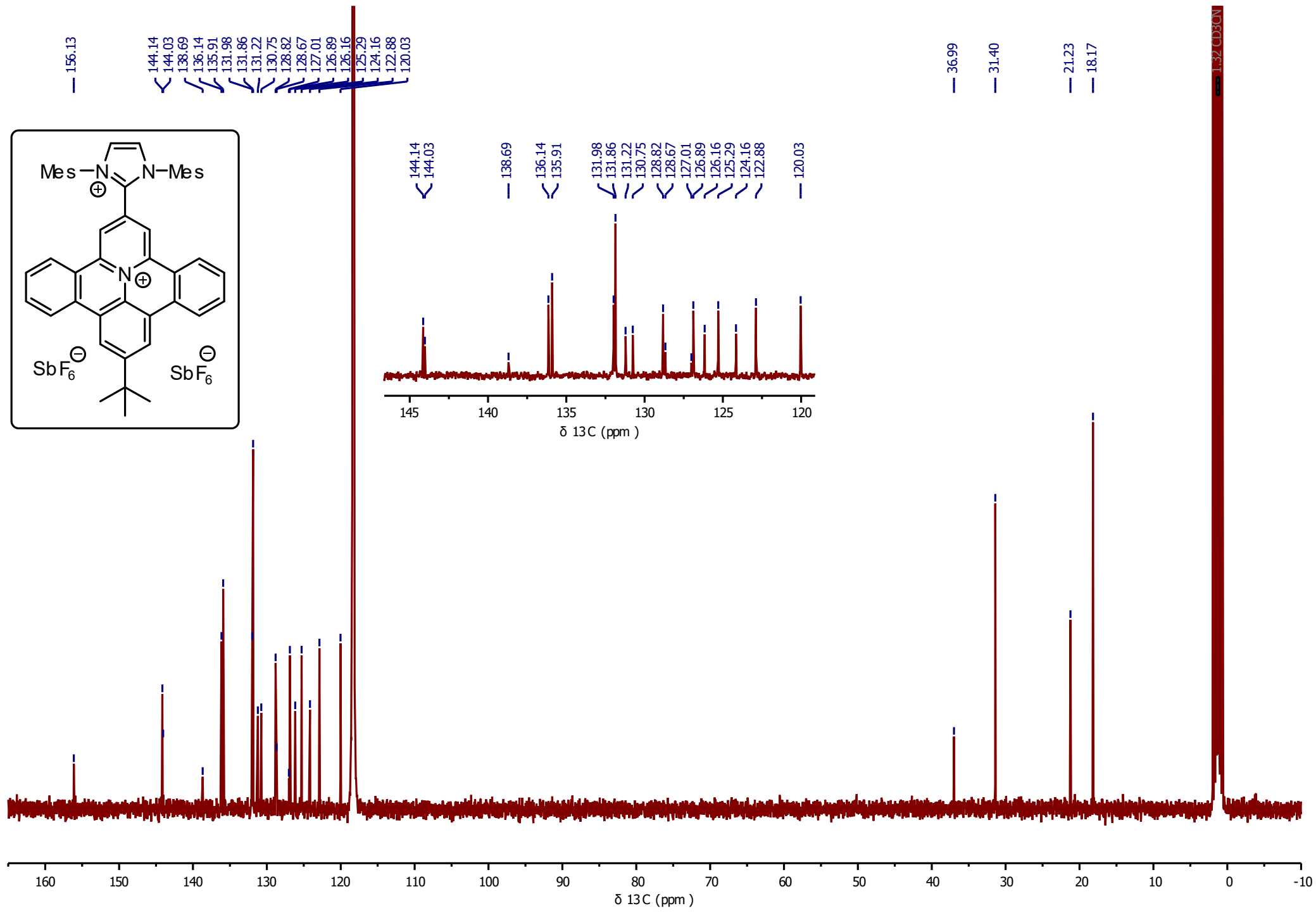

Fig. S56. ^{13}C {^{1}H} NMR (101 MHz, CD_3CN, 298K) of **PC^{2+}**.

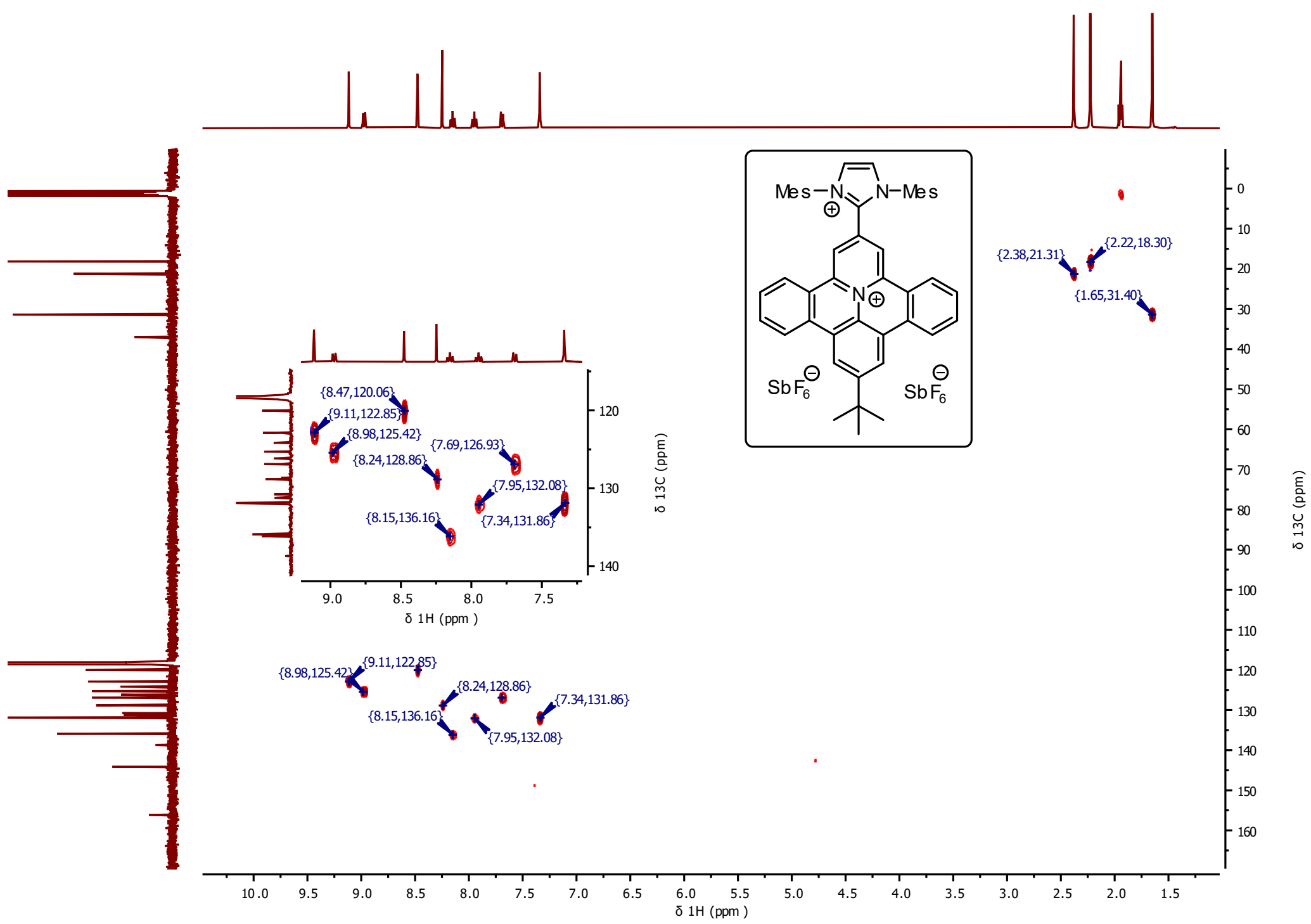

Fig. S57. ^{1}H/^{13}C HSQC (400/101 MHz, CD_3CN, 298K) of **PC^{2+}**.

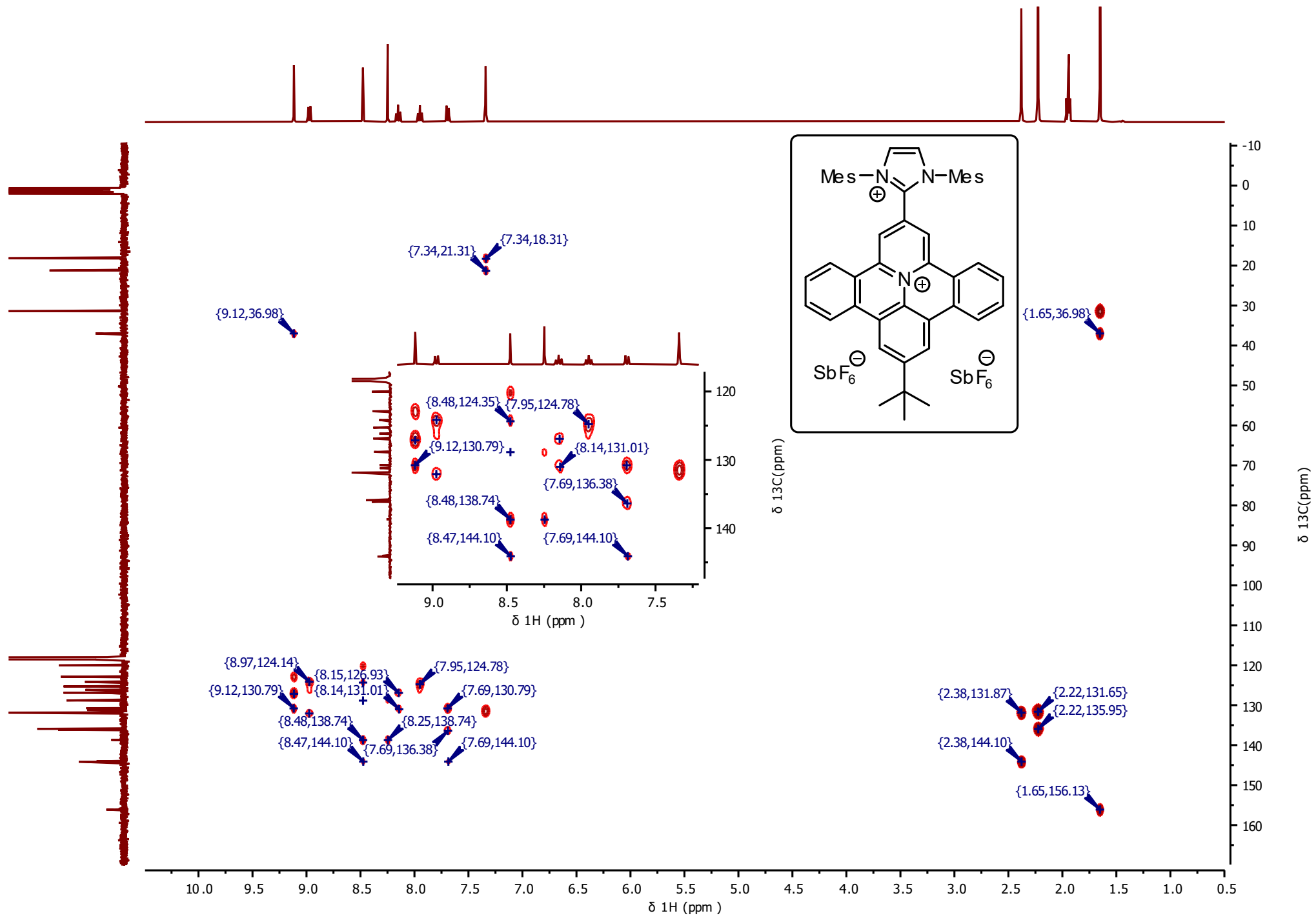

Fig. S58. $^{1}H/^{13}C$ HMBC (400/101 MHz, CD_3CN, 298K) of **PC^{2+}**.

References

1. T. J. B. Zähringer, J. A. Moghtader, M. Bertrams, B. Roy, M. Uji, N. Yanai and C. Kerzig, *Angew. Chem. Int. Ed.*, 2023, **62**, e202215340.
2. M.-S. Bertrams and C. Kerzig, *Chem. Commun.*, 2021, **57**, 6752–6755.
3. P. B. Merkel, P. Luo, J. P. Dinnocenzo and S. Farid, *J. Org. Chem.*, 2009, **74**, 5163–5173.
4. Ritu, M. Schmitz, C. Burdenski, P. W. Antoni, J. J. Holstein, C. Kerzig and M. M. Hansmann, *ACS Catal.*, 2025, **15**, 16718–16730.
5. P. Müller and K. Brettel, *Photochem. Photobiol. Sci.*, 2012, **11**, 632–636.
6. M. Goez, C. Kerzig and R. Naumann, *Angew. Chem. Int. Ed.*, 2014, **53**, 9914–9916.
7. G. Siano, S. Crespi and S. M. Bonesi, *J. Org. Chem.*, 2020, **85**, 14012–14025.
8. Y. Wu, J. Shi, H. Chen, J. Zhao and W. Dong, *Sci. Total Environ.*, 2016, **566–567**, 86–92.
9. C. Baleizão and M. N. Berberan-Santos, *J. Chem. Phys.*, 2007, **126**, 204510.
10. Y. Tsuchiya, S. Diesing, F. Bencheikh, Y. Wada, P. L. Dos Santos, H. Kaji, E. Zysman-Colman, I. D. W. Samuel and C. Adachi, *J. Phys. Chem. A*, 2021, **125**, 8074–8089.
11. C. Bellatreccia, F. Fina and P. Ceroni, *Photochem. Photobiol. Sci.*, 2026, **25**, 1049–1063.
12. M. Villa, A. Fermi, F. Calogero, X. Wu, A. Gualandi, P. G. Cozzi, A. Troisi, B. Ventura and P. Ceroni, *Chem. Sci.*, 2024, **15**, 14739–14745.
13. J. Holcman and K. Sehested, *J. Phys. Chem.*, 1977, **81**, 1963–1966.
14. R. Ishimatsu, S. Matsunami, K. Shizu, C. Adachi, K. Nakano and T. Imato, *J. Phys. Chem. A*, 2013, **117**, 5607–5612.
15. P. W. Antoni and M. M. Hansmann, *J. Am. Chem. Soc.*, 2018, **140**, 14823–14835.
16. P. W. Antoni, T. Bruckhoff and M. M. Hansmann, *J. Am. Chem. Soc.*, 2019, **141**, 9701–9711.
